# Supplementary material for: Talking in primary care (TIP): protocol for a cluster-randomised controlled trial in UK primary care to assess clinical and cost-effectiveness of communication skills e-learning for practitioners on patients’ musculoskeletal pain and enablement
Source: BMJ Open. 2024 Mar 19;14(3):e081932. doi: 10.1136/bmjopen-2023-081932 (PMC10953007; doi:10.1136/bmjopen-2023-081932)
Supplement: Supplementary data [file bmjopen-2023-081932supp003.pdf]

Online Supplementary File 2: PIS and Consent Forms

Contents

Participant Information Sheet for Patients 2
Patient Consent Form 6
Participant Information Sheet for Practitioners 7
Practitioner Consent Form 12

Bishop FL, *et al. BMJ Open* 2024; 14:e081932. doi: 10.1136/bmjopen-2023-081932

Participant Information Sheet for Patients

Version 2. Date 22.6.22.

Participant Information Sheet

Chief Investigators: Dr Felicity Bishop and Professor Hazel Everitt, University of Southampton

We invite you to take part in the TIP study

Please read on to find out why we are doing this study and what it involves. To help you decide whether to take part you may like to talk to others. If you want to talk to us, the researchers, or ask us some questions, please email <insert local researcher email address> or phone < local researcher number>. If you want to take part, you can tell us by answering the questions at the bottom of this page.

What if I need some help to take part?

We want lots of different people to take part in this study. And we know that different people will need different kinds of help.

We can help with things like understanding the documents, or if you have problems using the internet or if you would prefer paper copies of things posted to you.

If you need an interpreter, you are welcome to ask a family member or friend to help you with this study. Or you can ask us and we will do our best to get an interpreter for you.

If you would like some help to understand or take part in this study, please get in touch with us. You can contact us by phone < local researcher number >or email <<insert local researcher email address>>

A quick summary of the study

- This study will help us understand patients' experiences of appointments with GPs, Nurses, or Physiotherapists. We'll call these people "Primary Care Practitioners".
- We want to know what you think about how your Primary Care Practitioner talks to you during consultations.
- The study is being run by The Universities of Southampton, Bristol, Warwick, Oxford, and Keele University. It is funded by the National School for Primary Care Research (SPCR).
- The South Central-Hampshire B Research Ethics Committee has given a favourable opinion of the study. This means that a group of independent people have looked at our research and feel that it is ethically acceptable.

Why have I been asked to participate?

Because you are an adult and have an appointment with a Primary Care Practitioner who is already taking part in the TIP study

What will happen if I take part?

We will ask you to read some documents (like this one) and fill in some questionnaires.

Before your appointment

Read this information.
Answer a few questions to check you can take part in the study.
Answer a few questions to tell us if you want to take part in the study.

During your appointment

Your consultation at your GP surgery will happen as normal.
Please do not discuss this study with your Primary Care Practitioner.
It is important that your Primary Care Practitioner does not know whether you are taking part.

Within 1 week of your appointment

You will be sent a link to fill in some questionnaires. These questionnaires ask about your appointment, your health, and your quality of life. They take about 15 minutes to do.

1 month, 3 months, and 6 months after your first appointment

You will be sent a link to fill in some questionnaires. These questionnaires ask about your health and quality of life. They take about 15 minutes to do.

We might also invite you to take part in two meetings (interviews) with a researcher. If you are asked to do an interview, this would be in the first week after your appointment and again in 6 months' time. In the interview, the researcher will ask about your experiences of primary care appointments and your experiences of doing this study..

What are the possible pros and cons of taking part?

Taking part will help us understand the best ways for primary care practitioners to talk to patients during consultations. We do not think that taking part in this study poses any risks for you To thank you for taking part we will give you two £10 vouchers. We will send the first voucher after you complete the 1 month questionnaire. We will send the second voucher after you complete the 6 month questionnaire. If you take part in an interview as well as doing the questionnaires, then we will send you an extra £10 voucher for each interview.

Bishop FL, *et al. BMJ Open* 2024; 14:e081932. doi: 10.1136/bmjopen-2023-081932

Do I have to take part?

No, it is up to you to decide .

If you decide to take part now, you can still change your mind later. You can pull out from the study at any time by contacting the researcher by email or phone. You won't have to give a reason. Your routine health care won't be affected at all. If you pull out of the study, we will keep the information that you've already given us.

What information will be collected?

You will probably fill in our questionnaires on the internet. Although, if you would rather have a paper questionnaire please ask us and we can give you one.

Our questionnaires are on a secure service called Qualtrics. Qualtrics meets the highest standards for privacy and data security. We will download all the completed questionnaires. We will store this data on a University of Southampton computer server behind the University of Southampton firewall. At the end of the study, we will destroy our records of your personal contact details.

Your name will not appear on any questionnaires you fill in. Your questionnaire answers will be combined with other patients' answers and put in a secure data archive. Only suitably qualified researchers are allowed to ask for access this archive.

One of our questions asks if it's OK to use your questionnaire answers to help other ethically approved research and education activities in the future. If you say "no" you can still take part in the study. Personal data will be collected and stored on a secure server at University of Southampton in compliance with the requirements of the General Data Protection Regulations and the Data Protection Act 2018. We will securely store your name, contact details, and any other personal data you have given us in a separate list, so we know who has taken part. We will only use your contact details to contact you about this study. You do not need to but if you would like to read the full Data Protection Privacy Notice, click here.

Will my participation be confidential?

Your participation and the information we collect about you during the course of the research will be kept strictly confidential.

Nothing you say on the questionnaires or in the interviews will be shared with your Primary Care Practitioner or anyone else in the medical practice.

But, if you say something in an interview which makes the interviewer worried that you might be being abused or neglected then they will raise this with the appropriate people.

The research team may have to give certain other people access to your data. The only other people who might be given access to your data are responsible members of the University of Southampton and regulatory authorities (for example, the Health Research Authority). They need access to make sure the research is being done correctly and in line with regulations. All of these people must keep your information, strictly confidential.

What will happen to the results of the research?

We hope to publish our results in scientific journals, blogs and conferences. We plan to share our findings with a wide audience including health care professionals, scientists, patients and members of the public. If you would like, we can also send you a summary what we found out. You can ask for this summary when you fill in the questionnaires.

What happens if there is a problem?

If you have a concern about any aspect of this study, you should speak to Nadia Cross who will do her best to answer your questions.

If you remain unhappy or have a complaint about any aspect of this study, please contact the University of Southampton Research Integrity and Governance Manager (023 8059 5058, rgoinfo@soton.ac.uk).

You may also contact your local Patient Advice and Liaison Service (PALS). PALS has been introduced to ensure that the NHS listens to patients, their relatives, carers, and friends, and answers their questions and resolves their concerns as quickly as possible. Your local PALS service can be found at <<INSERT LOCAL DETAILS>>

Where can I get more information?

PLEASE DO NOT DISCUSS YOUR PARTICIPATION IN THE STUDY WITH YOUR GP, NURSE, PHYSIOTHERAPIST, OR ANY OTHER PRIMARY CARE PRACTITIONER.

If you have any questions about the study, you can contact the researcher, <<INSERT NAME>>

Email: <<INSERT>>; or Telephone: TBC>>

You can also contact the study manager, Nadia Cross at tip@soton.ac.uk

Thank you for reading this information and considering taking part in our study.

Patient Consent Form

Version 2. Date 22.6.22.

Patient Consent Form

Chief Investigators: Dr Felicity Bishop and Professor Hazel Everitt, University of Southampton

**Please indicate if you agree with the statement.**	**Yes/No**
I have read and understood the information sheet *(insert date /version no. of participant information sheet)* and have had the opportunity to ask questions about the study.	Yes/No
I agree to take part in this research project and agree for my data to be used for the purpose of this study.	Yes/No
I understand my participation is voluntary and I may withdraw at any time without giving a reason and without my routine health care being affected.	Yes/No
I understand that personal details I provide will be held securely at The University of Southampton in line with General Data Protection Regulation and Data Protection Act 2018.	Yes/No
I agree to take part in this study	Yes/No
Optional: You do not have to agree to this to take part in this research<br><br>I agree that the information collected about me may be used to support other ethically approved research and education activities in the future, and may be stored in a secure data archive and shared anonymously with other suitably-qualified researchers.	Yes/No

Participant Information Sheet for Practitioners

Version 1. Date 23.3.22.

Practitioner Information Sheet (main study)

Chief Investigators: Dr Felicity Bishop and Professor Hazel Everitt, University of Southampton

We invite you to take part in a research study

It is up to you to decide if you want to take part or not. This leaflet tells you why the study is being done and what it will involve. Please discuss this information with others if you wish. Please contact the research team if anything is unclear or you would like to ask any questions.

A quick summary of the study

- In this cluster randomised trial, your practice will be randomised into one of two groups: intervention arm or control arm.
- Practitioners working in intervention practices will complete communication skills e-learning training and implement the skills in subsequent consultations. Practitioners working in control practices will continue consulting as usual.
- Patients will be recruited at the intervention and control practices, and complete pre-consultation and post-consultation questionnaires.
- Practitioners in both arms will be asked to complete online questionnaires (about communication within consultations) at 3 time points: baseline, 8 weeks, and 34 weeks post-randomisation. Practitioners in the control group will have access to the communication skills e-learning training at the end of the study.
- The study is being run by the Universities of Southampton, Bristol, Keele, Oxford and Warwick, and is funded by the National School for Primary Care Research (SPCR).

What is the research about?

We have developed communication skills e-learning training for GPs, physiotherapists, and nurses to help enhance consultations with osteoarthritis patients. It is also likely that this training will be relevant to other conditions. The TIP (Talking in Primary Care) study aims to test the effectiveness and cost-effectiveness of communication skills e-learning training for primary care practitioners on patients' musculoskeletal pain and enablement.

Why have I been asked to participate?

You have been asked to take part because you are a GP, physiotherapist or nurse working in primary care, and have experience of treating patients with osteoarthritis. We hope to recruit a range of practitioners with different levels of experience and background.

Do I have to take part?

No, it is entirely up to you to decide whether or not to take part. If you decide that you would like to take part, we will ask you to complete an online consent form.

What will happen to me if I take part?

If you are interested in taking part:

- You will be provided with a link to a study website, provide online consent and complete an online questionnaire (approx. 10 minutes).
- Your practice will be randomised to one of two groups: an intervention arm and a control arm.
- In weeks 1-2, if you are in the intervention arm you will be asked to complete the training. This will take approximately 1-2 hours and can be done in short chunks. If you are in the control arm, you should continue to treat patients as usual and not undertake any training in communication skills.
- In weeks 3-8, we will be recruiting patients from your practice to take part in this study. You may be asked to help with this.
- You will be asked to complete a short online questionnaire about communication within consultations at 3 time points: baseline, 8 weeks, and 34 weeks post-randomisation.
- You will also be offered the opportunity to take part in a research interview to share your experiences of communication within consultations and the TIP study.
- If you are in the control arm, you will be offered access to the e-learning training at the end of the study.

What are the possible pros and cons of taking part?

Participating in the TIP study will give you the opportunity to learn and implement evidence-based communication skills within your consultations. This could improve patient outcomes and patient satisfaction with care and make best use of primary care appointments. There are no expected risks or disadvantages associated with taking part in this study.

GP practices will be paid service support costs/ excess treatment costs via their CRN for taking part in the TIP study. We will also provide research costs to reimburse practitioners for their time spent taking part in the study.

What happens to the data collected?

- Electronic questionnaires will be collected using a secure online data collection service which meets the highest industry standards for privacy and data security (Qualtrics).
- Data on patterns and amount of usage of the e-learning training will be collected by the LifeGuide platform on which the e-learning training is hosted.
- All data from Qualtrics and LifeGuide will be downloaded to University of Southampton servers, password-protected and stored securely behind the University of Southampton firewall.
- At the end of the study anonymous questionnaire data will be deposited in a secure data archive which will be made available on request to suitably qualified researchers for further data analysis on this topic.

We will securely store your name and contact details separately from your questionnaire data and will only use these details to contact you about this study. We will permanently delete this at the end of the project.

Will my participation be confidential?

Your participation and the information we collect about you during the course of the research will be kept strictly confidential.

Only members of the research team and responsible members of the University of Southampton may be given access to data about you for monitoring purposes and/or to carry out an audit of the study to ensure that the research is complying with applicable regulations. Individuals from regulatory authorities (people who check that we are carrying out the study correctly) may require access to your data. All of these people have a duty to keep your information, as a research participant, strictly confidential.

What happens if I change my mind?

You have the right to change your mind and withdraw at any time without giving a reason. If you wish to withdraw from the study, please contact Nadia Cross, Trial Manager (details below).

What will happen to the results of the research?

We hope to publish our results in scientific journals and other formats such as blogs and conferences. We plan to share our findings with a wide audience including health care professionals, scientists, patients, and members of the public. If you would like, we will also send you a summary of our findings.

Who is conducting the study?

Our research team includes GPs, health psychologists, academic researchers and patient representatives from the Universities of Southampton, Bristol, Keele, Oxford and Warwick. The research is funded by National School for Primary Care Research (SPCR) and has been approved by the Health Research Authority and the National Research Ethics Committee (reference number: <<xxxxxxxx>>). The research is being sponsored by University of Southampton.

What happens if there is a problem?

If you have a concern about any aspect of this study, you should speak to the researchers (contact details above) who will do their best to answer your questions.

If you remain unhappy or have a complaint about any aspect of this study, please contact the University of Southampton Research Integrity and Governance Manager (023 8059 5058, rgoinfo@soton.ac.uk).

Where can I get more information?

If you have any questions, please do not hesitate to get in touch with Nadia Cross, Trial Manager using the contact details below:

**Name**	**Nadia Cross**	
**Role:**	Trial Manager	
**Address:**	University of Southampton<br>Aldermoor Health Centre<br>Southampton, SO16 5ST	
**Contact:**	*[insert study team contact details@soton.ac.uk]*	

Thank you for taking the time to read the information sheet and considering taking part in the research

** Click out page in Qualtrics*

Data Protection Privacy Notice

The University of Southampton conducts research to the highest standards of research integrity. As a publicly-funded organisation, the University has to ensure that it is in the public interest when we use personally-identifiable information about people who have agreed to take part in research. This means that when you agree to take part in a research study, we will use information about you in the ways needed, and for the purposes specified, to conduct and complete the research project. Under data protection law, 'Personal data' means any information that relates to and is capable of identifying a living individual. The University's data protection policy governing the use of personal data by the University can be found on its website (https://www.southampton.ac.uk/legalservices/what-we-do/data-protection-and-foi.page).

This Participant Information Sheet tells you what data will be collected for this project and whether this includes any personal data. Please ask the research team if you have any questions or are unclear what data is being collected about you.

Bishop FL, *et al. BMJ Open* 2024; 14:e081932. doi: 10.1136/bmjopen-2023-081932

Our privacy notice for research participants provides more information on how the University of Southampton collects and uses your personal data when you take part in one of our research projects and can be found at http://www.southampton.ac.uk/assets/sharepoint/intranet/ls/Public/Research%20and%20Integrity%20Privacy%20Notice/Privacy%20Notice%20for%20Research%20Participants.pdf

Any personal data we collect in this study will be used only for the purposes of carrying out our research and will be handled according to the University's policies in line with data protection law. If any personal data is used from which you can be identified directly, it will not be disclosed to anyone else without your consent unless the University of Southampton is required by law to disclose it.

Data protection law requires us to have a valid legal reason ('lawful basis') to process and use your Personal data. The lawful basis for processing personal information in this research study is for the performance of a task carried out in the public interest. Personal data collected for research will not be used for any other purpose.

For the purposes of data protection law, the University of Southampton is the 'Data Controller' for this study, which means that we are responsible for looking after your information and using it properly. The University of Southampton will keep identifiable information about you for 10 years after the study has finished after which time any link between you and your information will be removed.

To safeguard your rights, we will use the minimum personal data necessary to achieve our research study objectives. Your data protection rights – such as to access, change, or transfer such information - may be limited, however, in order for the research output to be reliable and accurate. The University will not do anything with your personal data that you would not reasonably expect.

If you have any questions about how your personal data is used, or wish to exercise any of your rights, please consult the University's data protection webpage (https://www.southampton.ac.uk/legalservices/what-we-do/data-protection-and-foi.page) where you can make a request using our online form. If you need further assistance, please contact the University's Data Protection Officer (data.protection@soton.ac.uk).

Practitioner Consent Form

Version 2. Date 22.6.22.

Practitioner consent form (main study)

Chief Investigators: Dr Felicity Bishop and Professor Hazel Everitt, University of Southampton

ERGO number: 70489

Please indicate if you agree with the statements:

1. I have read and understood the practitioner information sheet (<<insert version and date>> and have had the opportunity to ask questions about the study.	Yes/No
2. I agree to take part in this research project and agree for my data to be used for the purpose of this study.	Yes/No
3. I understand my participation is voluntary and I may withdraw at any time for any reason.	Yes/No
4. I understand that personal details I provide will be held securely at The University of Southampton in line with General Data Protection Regulation and Data Protection Act 2018.	Yes/No
5. I agree to take part in the TIP study.	Yes/No
Optional: You do not have to agree to this item to take part in this research 6. I agree that my questionnaire data may be used to support other ethically approved research and education activities in the future and may be stored in a secure data archive and shared anonymously with other suitably qualified researchers.	Yes/No

Name of participant ………………………………………………………………………………

Date………………………………………

Bishop FL, *et al. BMJ Open* 2024; 14:e081932. doi: 10.1136/bmjopen-2023-081932
