# Supplementary material for: Talking in primary care (TIP): protocol for a cluster-randomised controlled trial in UK primary care to assess clinical and cost-effectiveness of communication skills e-learning for practitioners on patients’ musculoskeletal pain and enablement
Source: BMJ Open. 2024 Mar 19;14(3):e081932. doi: 10.1136/bmjopen-2023-081932 (PMC10953007; doi:10.1136/bmjopen-2023-081932)
Supplement: Supplementary data [file bmjopen-2023-081932supp004.pdf]

Online Supplementary File 3: Measures and Timings

Contents

Table 1. Patient-Reported Characteristics, Primary and Secondary Outcomes and Process Variables........... 2
Table 2. Practitioner-Reported Characteristics, Outcomes and Process Variables... 3
References.. 4

Bishop FL, *et al. BMJ Open* 2024; 14:e081932. doi: 10.1136/bmjopen-2023-081932

Table 1. Patient-Reported Characteristics, Primary and Secondary Outcomes and Process Variables

Variable	Measure	Items	Measurement Timings				
			<-7d	<7 d	+1 m	+3 m	+6 m
Primary Outcomes							
Pain intensity (pain sample)	Pain intensity subscale from the BPI[1]	4	x	x	x	x	x
Patient enablement	Modified PEI[2]	6		x	x	x	x
Secondary Outcomes							
Patient global impression of symptom severity	Single item[3]	1	x	x	x	x	x
Patient global impression of symptom change	Single item[3]	1		x	x	x	x
Pain interference	Pain interference subscale from the BPI[1]	7			x		X
Patient satisfaction	MISS for UK general practice[4]	21		x			
Adverse events	Bespoke self-report item	1			x	x	x
Health Economics							
Health-related quality of life	EQ-5D-5L and EQ-VAS[5]	6	x		x		x
Capability wellbeing	ICECAP-A[6 7]	5	x		x		x
Healthcare utilization	ModRUM core module[8]	12		x		x	x
Prescribed medications	ModRUM depth questions[8]	1				x	x
Personal expenses	Bespoke self-report item	3				x	x
Productivity	WPAI:GH	6				x	x
Process Measures							
Perceptions of practitioner empathy	CARE[9]	10		X			
Perceptions of practitioner optimism	Bespoke item	1		X			
Treatment expectations	Treatment expectation questionnaire TEX-Q[10]	15		X			
Anxiety	HADS[11 12]	7		X			
Continuity of care	Patient-Doctor Depth of Relationship Scale[13]	9		X			
Depression	HADS[11 12]	7		X			
Sociodemographic Characteristics							
Age, gender, ethnicity		3	x				
Index of Multiple Deprivation	Postcode	1	x				
Health Characteristics							
Reasons for consulting		1		x			
Comorbidities		1		x			
Index consultation modality		1		x			

Bishop FL, et al. BMJ Open 2024; 14:e081932. doi: 10.1136/bmjopen-2023-081932

Table 2. Practitioner-Reported Characteristics, Outcomes and Process Variables

Practitioners	Variable	Measure	Items	Measurement Timings			
				Baseline	+2wk	+8wk	+34wk
All	Characteristics (age, gender, ethnicity, years qualified, profession)	Bespoke	5	x			
All	Practitioner self-efficacy for conveying clinical empathy	Bespoke, from feasibility study	7	X		X	x
All	Practitioner self-efficacy for conveying realistic optimism	Bespoke, from feasibility study	5	x		X	x
Intervention arm only	Practitioner outcome expectancy for implementing goals set during EMPathicO training	Bespoke, from feasibility study	16	X		X	x
Intervention arm only	Practitioner intentions to implement goals set during EMPathicO training	Bespoke, from feasibility study	3	X		X	x
Intervention arm only	Practitioner intervention usage	LifeGuide data	N/A			X	X
All	Practitioner-reported other training	Bespoke	1			x	x

References

1. Keller S, Bann CM, Dodd SL, et al. Validity of the Brief Pain Inventory for use in documenting the outcomes of patients with noncancer pain. *Clin J Pain* 2004;20(5):309-18.
2. Howie JG, Heaney DJ, Maxwell M, et al. A comparison of a Patient Enablement Instrument (PEI) against two established satisfaction scales as an outcome measure of primary care consultations. *Fam Pract* 1998;15(2):165-71.
3. Fischer D, Stewart AL, Bloch DA, et al. Capturing the Patient's View of Change as a Clinical Outcome Measure. *JAMA* 1999;282(12):1157-62. doi: 10.1001/jama.282.12.1157
4. Meakin R, Weinman J. The 'Medical Interview Satisfaction Scale' (MISS-21) adapted for British general practice. *Fam Pract* 2002;19(3):257-63. doi: 10.1093/fampra/19.3.257
5. Herdman M, Gudex C, Lloyd A, et al. Development and preliminary testing of the new five-level version of EQ-5D (EQ-5D-5L). *Qual Life Res* 2011;20(10):1727-36. doi: 10.1007/s11136-011-9903-x [published Online First: 2011/04/12]
6. Al-Janabi H, Flynn TN, Coast J. Development of a self-report measure of capability wellbeing for adults: the ICECAP-A. *Qual Life Res* 2012;21(1):167-76. doi: 10.1007/s11136-011-9927-2 [published Online First: 2011/05/21]
7. Keeley T, Coast J, Nicholls E, et al. An analysis of the complementarity of ICECAP-A and EQ-5D-3 L in an adult population of patients with knee pain. *Health and quality of life outcomes* 2016;14:36. doi: 10.1186/s12955-016-0430-x [published Online First: 2016/03/05]
8. Garfield K, Husbands S, Thorn JC, et al. Development of a brief, generic, modular resource-use measure (ModRUM): cognitive interviews with patients. *BMC Health Serv Res* 2021;21(1):371. doi: 10.1186/s12913-021-06364-w
9. Mercer SW, Maxwell M, Heaney D, et al. The development and preliminary validation of the Consultation and Relational Empathy (CARE) measure: an empathy-based consultation process measure. *Fam Pract* 2004;21 699-705.
10. Alberts J, Löwe B, Glahn MA, et al. Development of the generic, multidimensional Treatment Expectation Questionnaire (TEX-Q) through systematic literature review, expert surveys and qualitative interviews. *BMJ Open* 2020;10(8):e036169. doi: 10.1136/bmjopen-2019-036169
11. Bjelland I, Dahl AA, Haug TT, et al. The validity of the Hospital Anxiety and Depression Scale: An updated literature review. *J Psychosom Res* 2002;52(2):69-77.
12. Zigmond AS, Snaith RP. The Hospital Anxiety and Depression Scale. *Acta Psychiatr Scand* 1983;67(6):361-70.
13. Ridd MJ, Lewis G, Peters TJ, et al. Patient-Doctor Depth-of-Relationship Scale: Development and Validation. *The Annals of Family Medicine* 2011;9(6):538. doi: 10.1370/afm.1322
