# Supplementary material for: Talking in primary care (TIP): protocol for a cluster-randomised controlled trial in UK primary care to assess clinical and cost-effectiveness of communication skills e-learning for practitioners on patients’ musculoskeletal pain and enablement
Source: BMJ Open. 2024 Mar 19;14(3):e081932. doi: 10.1136/bmjopen-2023-081932 (PMC10953007; doi:10.1136/bmjopen-2023-081932)
Supplement: Supplementary data [file bmjopen-2023-081932supp001.pdf]

Talking in Primary Care: A cluster-randomized controlled trial in primary care to test the effectiveness and cost-effectiveness of communication skills e-learning for practitioners on patients' musculoskeletal pain and enablement

Trial Steering Committee Charter

Version 1 22 April 2022

Authorised by:

Name:	Professor Joanne Reeve	Role:	Chairperson
Signature:	Joanne Reeve	Date:	22 April 2022

Prepared by

Name:	Nadia Cross	Role:	Trial Manager
Signature:		Date:	22 April 2022

CONTENT	DETAILS OF TSC
**1. Introduction**	
Name (& Sponsor's ID) of trial	Talking in Primary Care: A cluster-randomized controlled trial in primary care to test the effectiveness and cost-effectiveness of communication skills e-learning for practitioners on patients' musculoskeletal pain and enablement.<br>**UoS ERGO:** 70489<br>**IRAS:** 312208
Objectives of trial, including interventions being investigated	The primary aim is to determine the clinical and cost-effectiveness of EMPathicO training in Clinical Empathy and conveying realistic Positive Messages for practitioners in patients presenting with MSK pain.<br><br>The secondary aim is to maximize EMPathicO's potential for wide-spread adoption, implementation, and maintenance of effects. We will do this by assessing effects of EMPathicO training on patients presenting with any symptoms other than MSK pain since the impact of EMPathicO will potentially be in all consultations not just MSK consultations; testing how and in what circumstances EMPathicO changes practitioner communication behaviours and patient outcomes for in-person, telephone, and video consultations; and analysing a diverse range of patients' and practitioners' experiences of adoption and longer-term implementation.
Outline of scope of Charter	The purpose of this document is to describe the membership, terms of reference, roles, responsibilities, authority, decision-making and relationships of the Trial Steering Committee (TSC) and the Data Monitoring Committee (DMC) for this trial, including the timing of meetings, methods of providing information to and from the TSC, frequency and format of meetings and relationships with other trial committees.
Facilitation	A member of the TIP team will be nominated as a Facilitator for the trial. The Facilitator will be responsible for the organisation of meetings and should be copied into all communications with and between the TSC.
**2. Roles and responsibilities**	
A broad statement of the aims of the TSC	TSC - To act as the oversight body for the TIP study on behalf of the Sponsor/Funder.<br><br>DMC - To monitor and review on a 6 monthly basis the main outcomes measures overall conduct in order to safeguard the interests of patients
Terms of reference	The role of the TSC is to provide oversight for the TIP study. It should also provide advice through its independent Chairperson to the Trial Management Group (TMG) and the funder (NIHR-SPCR) on all aspects of the trial.<br><br>The TSC will also assume responsibilities of the Data Monitoring Committee (DMC) and review information on the progress and accruing data of this trial and provide advice on the conduct of the trial.

CONTENT	DETAILS OF TSC
Specific roles of TSC	• provide expert oversight of the trial<br>• maintain confidentiality of all trial information that is not already in the public domain<br>• make decisions as to the future continuation (or otherwise) of the trial/s<br>• monitor recruitment rates and encourage the TMG to develop strategies to deal with any recruitment problems<br>• comment on the protocol<br>• assess the impact and relevance of any accumulating external evidence<br>• review completion of CRFs and comment on strategies from TMG to encourage satisfactory completion in the future<br>• monitor follow-up rates and review strategies from TMG to deal with problems<br>• censure sites that are deviating from the protocol<br>• comment on any amendments to the protocol, where appropriate<br>• approve any proposals by the TMG concerning any change to the design of the trial, including additional sub-studies<br>• oversee the timely reporting of trial results<br>• comment on the statistical analysis plan<br>• comment on the publication policy<br>• comment on the main trial manuscript<br>• comment on any abstracts and presentations of any results during the running of the trial
Specific roles of DMC delegated to the TSC	Interim review of the trial's progress including updated figures on recruitment, data quality, adherence to protocol treatment and follow-up, and main outcomes and safety data. Specifically, these roles include to:<br>• monitor evidence for treatment harm (e.g. SAEs and deaths)<br>• assess the impact and relevance of external evidence<br>• decide whether to recommend that the trial continues to recruit participants or whether recruitment should be terminated either for everyone or for some treatment groups and/or some participant subgroups<br>• decide whether trial follow-up should be stopped earlier<br>• assess data quality, including completeness (and by so doing encourage collection of high quality data)<br>• maintain confidentiality of all trial information that is not in the public domain<br>• monitor recruitment figures and losses to follow-up<br>• monitor compliance with the protocol by participants and investigators<br>• monitor planned sample size assumptions.<br>• suggest additional data analyses if necessary<br>• advise on protocol modifications proposed by investigators or sponsors (e.g. to inclusion criteria, trial endpoints, or sample size)<br>• monitor continuing appropriateness of patient information

CONTENT	DETAILS OF TSC
**3. Before or early in the trial**	
Whether the TSC will have input into the protocol	All potential TSC members should have sight of the protocol as early as possible. Before recruitment begins the trial will have undergone review by the Sponsor/Funder (e.g. peer review for public sector trials), scrutiny by other trial committees and a research ethics committee. Therefore, if a potential TSC member has major reservations about the trial (e.g. the protocol, the logistics, ethical concerns) they should report these to TMG. TSC members should be constructively critical of the ongoing trial, but also supportive of aims and methods of the trial.
Whether members of the TSC will have a contract	TSC members will not be asked to formally sign a contract but should formally register their agreement to join the group by confirming (1) that they agree to be a member and (2) that they agree with the contents of this Charter. Any potential competing interests should be declared at the same time. Members should complete and return the form in Annexes 1 or 2. Any observers (attendees who are not members) will sign a confidentiality agreement on the first occasion they attend a meeting (Annexe 3).
**4. Composition**	
Membership and size of the TSC	The majority of members of the TSC, including the Chair, should be independent [1]of the trial (see section 5). Non-independent members will also be part of the TSC.<br>The members of the TSC for this trial are:<br>Professor Joanne Reeve (chair) – Independent member<br>Dr Philip Pallmann – Independent member<br>Dr Ines Rombach – Independent member<br>Mr Ian Dickerson – PPI contributor<br>Dr Felicity Bishop – Co-Chief Investigator<br>Professor Hazel Everitt – Co-Chief Investigator
Tenure	Until 30/06/2024.
The Chair, how they are chosen and the Chair's role.	The Chair should have previous experience of serving on trial committees and experience of Chairing meetings, and should be able to facilitate and summarise discussions; knowledge of the disease area would be beneficial.
The responsibilities of the Facilitator	The Facilitator will be a member of staff in Southampton Primary Care Research Centre, University of Southampton. The Facilitator will be responsible for arranging meetings of the TSC, coordinating reports, producing and circulating minutes and action points. The Facilitator will be the central point for all TSC communications between the TSC and other bodies, will be copied into all correspondence between TSC members and will be kept aware of trial issues as they arise.
The responsibilities of the TIP team	The TIP team will produce a short report on the trial before each meeting of the TSC.

[1] Independence is defined in Table 1 of Annexe 1

CONTENT	DETAILS OF TSC
The responsibilities of the CI and other members of the TMG	The CI (and, if appropriate, other TMG members) is an important member of the TSC and no major decisions should be made without their involvement.
The responsibilities of the observers	Additional observers may be in attendance through (parts of) the TSC meetings in order to provide input on behalf of the TIP team, the trial's Sponsor/Funder or to provide specific relevant expertise.
**5. Relationships**	
Advisory and executive bodies	The TSC is the oversight body and is delegated the roles in Section 2 by the Sponsor. All substantial issues regarding the trial must go to the TSC for consideration.
Payments to TSC members	Members will be reimbursed for reasonable travel costs and other expenses incurred. No other payments or rewards would be given professional members. Honoraria will be paid to lay members according to the INVOLVE guidelines.
The need for TSC members to disclose information about any real or potential competing interests	Any competing interests, both real or potential, should be disclosed. These are not restricted to financial matters – involvement in other trials or intellectual investment could be relevant. Although members may well be able to act objectively despite such connections, complete disclosure enhances credibility. (See Annex 1)<br>TSC members should not use any trial data to inform trading in pharmaceutical shares, and careful consideration should be given to trading in stock of companies with competing products. Changes in declarations of real or potential competing interests should be minuted at the start of each meeting.
**6. Organisation of meetings**	
Expected frequency of TSC meetings	The TSC will meet in person at least yearly if possible. At the request of the TSC, interim meetings, in person or by teleconference, will be organised. Major trial issues may need to be dealt with between meetings, by phone or by email. TSC members should be prepared for such instances.
Attendance of TSC members at meetings	Effort will be made to ensure that all members can attend. The Facilitator will work for a date that enables this. The CI must try to attend all meetings, especially if major actions are expected. Members who cannot attend in person should be encouraged to participate by teleconference. If, at short notice, any TSC members cannot attend then the TSC may still meet if at least two independent members, including the Chair (unless otherwise agreed), will be present, plus also a member of the trial team. If the TSC is considering a major action after such a meeting the TSC Chair should communicate with the absent members, including the CI, as soon after the meeting as possible to check they agree. If they do not, a further teleconference should be arranged with the full TSC.
How TSC meetings will be organised, especially regarding open and closed sessions,	Presence will be usually limited to the TSC members, observers from the Sponsor/Funder, TIP team and the Facilitator. Other attendees may be invited for all or part of the meeting by the TSC including

CONTENT	DETAILS OF TSC
including who will be present in each session	the trial statistician and trial manager. The observers are not members of the TSC but may be invited to provide expert input or to represent the funding bodies involved; other observers will be at the discretion of the TSC and the Facilitator but may include members of the TMG other than the CI.
Can TSC members who cannot attend the meeting input	If the report is circulated before the meeting, TSC members who will not be able to attend the meeting may pass comments to the TSC Chair, Facilitator or TIP team for consideration during the discussions.
What happens to independent members who do not attend meetings	If an independent member does not attend a meeting or provide comments when requested between meetings, it should be ensured that the independent member is available for the next meeting. If an independent member does not attend the next meeting or provide comments when next requested, they should be asked if they wish to remain part of the TSC.
**7. Trial documentation and procedures to ensure confidentiality and proper communication**	
Intended content of material to be considered during meetings	A short report will be prepared by the TIP team. This will report on accrual and any matters affecting the trial. Additionally, the material may include requests *from* the TMG or draft publications. Where relevant, accrual, compliance with follow-up and adherence to treatment may be presented by centre.
Whether reports to the TSC be available before the meeting or only at/during the meeting	It is usually helpful for the TSC to receive the report at least 1 week and preferably at least 2 weeks before any meetings. Different procedures may apply to teleconference meetings.
Responsibility for identifying and circulating external evidence (e.g. from other trials/ systematic reviews)	Identification and circulation of external evidence (e.g. from other trials/ systematic reviews) is not the responsibility of the TSC members; it is a responsibility of the TMG. However, the TSC should continue to be made aware of other data that may impact on a trial.
What will happen to the papers after the meeting	TSC members would be expected to delete, destroy or store securely copies of the reports to and from the TSC, agenda and minutes, as well as copies of communications between meetings. All documentation should be considered confidential. The Facilitator will keep a central record of all minutes, reports and correspondence by the TSC.
**8. Decision making**	
What decisions will be open to the TSC	Possible decisions include:-<br>• No action needed, trial continues as planned<br>• Early stopping due, for example, to clear benefit or harm of a treatment, futility or external evidence.<br>• Stopping recruitment within a subgroup.<br>• Modifying target recruitment, or pre-analysis follow-up, based on any change to the assumptions underlying the original trial sample size calculation (but not on any emerging differences)<br>• Sanctioning and/or proposing protocol changes
How decisions or	Every effort should be made to achieve consensus. The role of the

CONTENT	DETAILS OF TSC
recommendations will be reached within the TSC	Chair is to summarise discussions and encourage consensus; therefore, it is usually best for the Chair to give their own opinion last.<br>It is important that the implications (e.g. ethical, statistical, practical, financial) for the trial be considered before any decision is made.
When the TSC is quorate for decision-making	At least two independent members of the TSC should be present including the Chair, plus the CI if a major action is to be considered.
**9. Reporting**	
To whom will the TSC report their recommendations/decisions, and in what form	The TSC will report their decisions (via the Facilitator) to the TMG who will be responsible for implementing any actions resulting. The TSC may also provide feedback to the Sponsor/Funder. Copies of communications will pass through the Facilitator.
Whether minutes of the meeting be made and, if so, by whom and where they will be kept	Notes of key points and actions will be made by the Facilitator. This will include details of whether potential competing interests have changed for any attendees since the previous meeting. The draft minutes will be initially circulated for comment to those TSC members who were present at the meeting. The TSC Chair will sign off the final version of minutes or notes.
**10. After the trial**	
Publication of results	The TSC will oversee the timely analysis, writing up and publication of the main trial results. The independent members of the TSC will have the opportunity to read and comment on the proposed main publications of trial data prior to submission and abstracts and presentations during the trial.
The information about the TSC that will be included in published trial reports	TSC members will be named and their affiliations listed in the main report, unless they explicitly request otherwise.

Abbreviations and glossary

AE	Adverse event
CF	Consent form
CI	Chief Investigator
CRF	Case Report Form
CTA	Clinical Trials Authorisation
CTU	Clinical Trials Unit
DMC	Data Monitoring Committee
HE	Health Economics
ISRCTN	International standard randomised controlled trial number
MRC	Medical Research Council
NHS	National Health Service
PI	Principal Investigator
PIS	Patient information Sheet
QL	Quality of life
SAE	Serious adverse event
SOP	Standard operating procedures
SSA	Site specific assessment
TMG	Trial Management Group
TSC	Trial Steering Committee

Annexe 1: Agreement and competing interests form for independent members

TIP Trial Steering Committee: Agreement to join the Trial Steering Committee as an independent member and disclosure of potential competing interests

Please complete the following document and return to the TSC Facilitator.

(please initial box to agree)

☐ I have read and understood the TSC Charter version 1.0, dated 22 April 2022

☐ I agree to join the Trial Steering Committee for this trial as an independent member

☐ I agree to treat all sensitive trial data and discussions confidentially

The avoidance of any perception that independent members of a TSC may be biased in some fashion is important for the credibility of the decisions made by the TSC and for the integrity of the trial.

Potential competing interests should be disclosed via the via the Primary Care Research Centre. In many cases simple disclosure up front should be sufficient. Otherwise, the (potential) independent TSC member should remove the conflict or stop participating in the TSC. **Table 1** lists potential competing interests.

☐ **No,** I have no potential competing interests to declare
☐ **Yes,** I have potential competing interests to declare (please detail below)

Please provide details of any potential competing interests:

____

____

____

Name: ________________________

Signed: ________________________ Date: ____________

Table 1: Potential competing interests for independent members

- Stock ownership in any commercial companies involved
- Stock transaction in any commercial company involved (if previously holding stock)
- Consulting arrangements with the Sponsor/Funder
- Ongoing advisory role to a company providing drugs to the trial
- Frequent speaking engagements on behalf of the intervention
- Career tied up in a product or technique assessed by trial
- Hands-on participation in the trial
- Involvement in the running of the trial
- Emotional involvement in the trial
- Intellectual conflict e.g. strong prior belief in the trial's experimental arm
- Involvement in regulatory issues relevant to the trial procedures
- Investment (financial or intellectual) or career tied up in competing products
- Involvement in the writing up of the main trial results in the form of authorship

Note: This TSC charter was developed using MRC CTU template TSC Charter version 1.02, 13-Mar-2006

Annexe 2: Agreement and competing interests form for non-independent members

TIP Trial Steering Committee: Agreement to join the Trial Steering Committee as an independent member and disclosure of potential competing interests

Please complete the following document and return to the Facilitator.

(please initial box to agree)

☐	I have read and understood the TSC Charter version 1.0, dated 22 April 2022
☐	I agree to join the Trial Steering Committee for this trial as an non-independent member
☐	I agree to treat all sensitive trial data and discussions confidentially

The avoidance of any perception that members of a TSC may be biased in some undisclosed fashion is important for the credibility of the decisions made by the TSC and for the integrity of the trial.

Possible competing interests should be disclosed via the Primary Care Research Centre. In many cases simple disclosure up front should be sufficient. Otherwise, the (potential) independent TSC member should remove the conflict or stop participating in the TSC. Table 1 lists potential competing interests.

☐ **No,** I have no competing interests to declare other than involvement in the trial
☐ **Yes,** I have competing interests to declare (please detail below)

Please provide details of any competing interests:

____
____
____

Name: ____________________________

Signed: ___________________________ Date: ______________

Table 1: Potential competing interests for non-independent members

- Stock ownership in any commercial companies involved
- Stock transaction in any commercial company involved (if previously holding stock)
- Consulting arrangements with the Sponsor/Funder
- Ongoing advisory role to a company providing drugs to the trial
- Frequent speaking engagements on behalf of the intervention
- Intellectual conflict e.g. strong prior belief in the trial's experimental arm
- Involvement in regulatory issues relevant to the trial procedures
- Investment (financial or intellectual) in competing products

Note: This TSC charter was developed using MRC CTU template TSC Charter version 1.02, 13-Mar-2006

Annexe 3: Agreement and confidentiality agreement for observers

TIP Trial Steering Committee: Agreement to attend the Trial Steering Committee and treat all information confidentially

Please complete the following document and return to the Facilitator.

(please initial box to agree)

☐	I have received a copy of the TSC Charter version 1.0 22 April 2022
☐	I agree to attend the Trial Steering Committee meeting on ___/____/______
☐	I agree to treat as confidential any sensitive information gained during this meeting unless explicitly permitted

Name: ____________________________

Signed: ___________________________ Date: ______________

Note: This TSC charter was developed using MRC CTU template TSC Charter version 1.02, 13-Mar-2006

Bishop FL, *et al. BMJ Open* 2024; 14:e081932. doi: 10.1136/bmjopen-2023-081932

Annexe 4: Summarise changes from previous version

Version 1.0

This is version 1.0 of the TSC charter for this trial. There are no changes to be reported.
