# Supplementary material for: Talking in primary care (TIP): protocol for a cluster-randomised controlled trial in UK primary care to assess clinical and cost-effectiveness of communication skills e-learning for practitioners on patients’ musculoskeletal pain and enablement
Source: BMJ Open. 2024 Mar 19;14(3):e081932. doi: 10.1136/bmjopen-2023-081932 (PMC10953007; doi:10.1136/bmjopen-2023-081932)
Supplement: Supplementary data [file bmjopen-2023-081932supp005.pdf]

Online Supplementary File 5

Stop-Go Progression Criteria

Progression criteria are based on recruitment rates 6 months after commencing patient recruitment:

• GREEN: Recruited 21 practices and 420 patients, with a good pipeline. Continue as planned.

• AMBER: Recruited 15-20 practices and at least 150 patients, with a good pipeline. Discuss with TSC and funder possible mitigating actions, e.g., increase staff time on recruitment activities, expand to other CRNs, shorten patient follow-up period.

• RED: Recruit <15 practices and <150 patients. Discuss with TSC and funder to explore all possible avenues to save the trial. If none deemed feasible, then stop.
