# Supplementary material for: Talking in primary care (TIP): protocol for a cluster-randomised controlled trial in UK primary care to assess clinical and cost-effectiveness of communication skills e-learning for practitioners on patients’ musculoskeletal pain and enablement
Source: BMJ Open. 2024 Mar 19;14(3):e081932. doi: 10.1136/bmjopen-2023-081932 (PMC10953007; doi:10.1136/bmjopen-2023-081932)
Supplement: Supplementary data [file bmjopen-2023-081932supp006.pdf]

Online Supplementary File 6

Plans to give access to the full protocol, participant level-data and statistical code

The protocol will be published in an open access journal. We will seek patient and practitioner consent to deposit data in a data archive e.g., for secondary analysis. For participants who consent for their data to be deposited in a data archive, we will take the necessary steps to pseudonymize the data prior to deposit. Data will be deposited in Pure, the University of Southampton's online data repository, where access will be restricted through gatekeepers (the chief investigators) to suitably qualified individuals with appropriate protocols in place. Statistical code will not be deposited as the pseudonymisation process alters the dataset in a way that impacts the applicability of the statistical code.
