# Supplementary material for: Talking in primary care (TIP): protocol for a cluster-randomised controlled trial in UK primary care to assess clinical and cost-effectiveness of communication skills e-learning for practitioners on patients’ musculoskeletal pain and enablement
Source: BMJ Open. 2024 Mar 19;14(3):e081932. doi: 10.1136/bmjopen-2023-081932 (PMC10953007; doi:10.1136/bmjopen-2023-081932)
Supplement: Author's manuscript [file bmjopen-2023-081932.draft_revisions.pdf]

BMJ Open

BMJ Open is committed to open peer review. As part of this commitment we make the peer review history of every article we publish publicly available.

When an article is published we post the peer reviewers' comments and the authors' responses online. We also post the versions of the paper that were used during peer review. These are the versions that the peer review comments apply to.

The versions of the paper that follow are the versions that were submitted during the peer review process. They are not the versions of record or the final published versions. They should not be cited or distributed as the published version of this manuscript.

BMJ Open is an open access journal and the full, final, typeset and author-corrected version of record of the manuscript is available on our site with no access controls, subscription charges or pay-per-view fees (http://bmjopen.bmj.com).

If you have any questions on BMJ Open's open peer review process please email info.bmjopen@bmj.com

BMJ Open

Talking in Primary Care (TIP): A cluster-randomised controlled trial in UK primary care to assess clinical and cost effectiveness of communication skills e-learning for practitioners on patients' musculoskeletal pain and enablement

Journal:	*BMJ Open*
Manuscript ID	bmjopen-2023-081932
Article Type:	Protocol
Date Submitted by the Author:	09-Nov-2023

Complete List of Authors:	Bishop, Felicity; University of Southampton, Psychology<br>Cross, Nadia; University of Southampton, Primary Care Research Centre, School of Primary Care, Population Science, and Medical Education<br>Dewar-Haggart, Rachel; University of Southampton, School of Psychology and Primary Care Research Centre, School of Primary Care, Population Science, and Medical Education<br>Teasdale, Emma; University of Southampton, School of Psychology and Primary Care Research Centre, School of Primary Care, Population Science, and Medical Education<br>Herbert, Amy; University of Bristol, Centre of Academic Primary Care, Bristol Medical School<br>Robinson, Michelle; Keele University, School of Primary, Community and Social Care<br>Ridd, Matthew; University of Bristol Faculty of Health Sciences, Population Health Sciences<br>Mallen, Christian; Keele University, Keele School of Medicine<br>Clarson, Lorna; Keele University, Keele School of Medicine<br>Bostock, Jennifer; University of Southampton, Primary Care Research Centre, School of Primary Care, Population Science, and Medical Education<br>Becque, Taeko; University of Southampton, Primary Care Research Centre, School of Primary Care, Population Science, and Medical Education<br>Stuart, Beth; Queen Mary University of London, Wolfson Institute of Population Health<br>Garfield, Kirsty; University of Bristol, Bristol Randomised Trials Collaboration<br>Morrison, Leanne; University of Southampton School of Psychology, Primary Care Research Centre, School of Primary Care, Population Science, and Medical Education,<br>Pollet, Sebastien; University of Southampton, School of Psychology and Primary Care Research Centre, School of Primary Care, Population Science, and Medical Education<br>Vennik, Jane; University of Southampton, Primary Care Research Centre, School of Primary Care, Population Science, and Medical Education<br>Atherton, Helen; University of Warwick<br>Howick, Jeremy; University of Leicester, Leicester Medical School;

University of Oxford, Faculty of Philosophy
Leydon, Geraldine; University of Southampton, Primary Care Research Centre, School of Primary Care, Population Science, and Medical Education
Nuttall, Jacqui; University of Southampton, Southampton Clinical Trials Unit
Islam, Nazrul; University of Southampton, Primary Care Research Centre, School of Primary Care, Population Science, and Medical Education
Lee, Paul; University of Southampton, Southampton Clinical Trials Unit; University Hospital Southampton NHS Foundation Trust, Southampton Clinical Trials Unit
Little, Paul; University of Southampton, Primary Care Research Centre, School of Primary Care, Population Science, and Medical Education
Everitt, Hazel; University of Southampton, Primary Care Research Centre, School of Primary Care, Population Science, and Medical Education

Keywords: Primary Health Care, eHealth, MEDICAL EDUCATION & TRAINING, Musculoskeletal disorders < ORTHOPAEDIC & TRAUMA SURGERY, Patient-Centered Care

SCHOLARONE™ Manuscripts

Talking in Primary Care (TIP): A cluster-randomised controlled trial in UK primary care to assess clinical and cost effectiveness of communication skills e-learning for practitioners on patients' musculoskeletal pain and enablement

For peer review only

Authors

Felicity L Bishop*, Nadia Cross, Rachel Dewar-Haggart, Emma Teasdale, Amy Herbert, Michelle Robinson, Matthew J Ridd, Christian Mallen, Lorna Clarson, Jennifer Bostock, Taeko Becque, Beth Stuart, Kirsty Garfield, Leanne Morrison, Sebastien Pollet, Jane Vennik, Helen Atherton, Jeremy Howick, Geraldine M Leydon, Jacqui Nuttall, Nazrul Islam, Paul H Lee, Paul Little, Hazel Everitt.

*Corresponding Author

Professor Felicity L Bishop, School of Psychology, University of Southampton, Highfield Campus, Southampton, UK, SO17 1BJ. Email F.L.Bishop@soton.ac.uk. Phone +44 (0)23 80599020.

Authors' Contributions

Allocated using CRediT categories. Study Conceptualisation and Funding Acquisition: HE, FB, JH, PL, BS, GL, LM, JV, JB, CM, LC, MRi, KG, HA. Methodology: All authors. Investigation: NC, RDH, ET, AH, MRo, SP. Project Administration: FB, HE, NC. Software: SP. Supervision: FB, HE. Writing – original draft: HE, FB, JH, BS, TB, MRi, KG, HA, JB. Writing – review and editing: All authors.

For peer review only - http://bmjopen.bmj.com/site/about/guidelines.xhtml

Author Details and Affiliations

Name	Email	ORCID	Job Title	Institution Affiliation
Felicity L Bishop	F.L.Bishop@soton.ac.uk	0000-0002-8737-6662	Professor of Health Psychology	School of Psychology, University of Southampton
Hazel A Everitt	H.A.Everitt@soton.ac.uk	0000-0001-7362-8403	Professor of Primary Care Research	Primary Care Research Centre, School of Primary Care, Population Science, and Medical Education, University of Southampton
Paul Little	P.Little@soton.ac.uk	0000-0003-3664-1873	Professor of Primary Care Research	Primary Care Research Centre, School of Primary Care, Population Science, and Medical Education, University of Southampton
Geraldine M Leydon	G.M.Leydon@soton.ac.uk	0000-0001-5986-3300	Professor in Primary Care	Primary Care Research Centre, School of Primary Care, Population Science, and Medical Education, University of Southampton
Beth Stuart	b.l.stuart@qmul.ac.uk	0000-0001-5432-7437	Professor of Medical Statistics	Wolfson Institute of Population Health, Queen Mary University of London
Leanne Morrison	L.Morrison@soton.ac.uk	0000-0002-9961-551X	Associate Professor in Health Psychology	School of Psychology and Primary Care Research Centre, School of Primary Care, Population Science, and Medical Education, University of Southampton
Jane Vennik	J.Vennik@soton.ac.uk	0000-0003-4602-9805	Senior Research Fellow	Primary Care Research Centre, School of Primary Care, Population Science, and Medical Education, University of Southampton
Christian Mallen	c.d.mallen@keele.ac.uk	0000-0002-2677-1028	Head, Keele School of Medicine	Keele School of Medicine, Keele University
Lorna Clarson	l.clarson@keele.ac.uk	0000-0003-0828-9649	Senior Lecturer in General Practice Research	Keele School of Medicine, Keele University
Matthew Ridd	m.ridd@bristol.ac.uk	0000-0002-7954-8823	Professor of Primary Healthcare	Centre of Academic Primary Care, Bristol Medical School, University of Bristol
Kirsty Garfield	kirsty.garfield@bristol.ac.uk	0000-0002-8301-3602	Research Fellow in Health Economic Evaluation	Health Economics Bristol, Population Health Sciences, Bristol Medical School, University of Bristol
Jeremy Howick	jh815@leicester.ac.uk	0000-0003-0280-7206	Professor of Empathic Healthcare and Director of the Stoneygate Centre for Empathic Healthcare; Impact Fellow	Leicester Medical School, University of Leicester; Faculty of Philosophy, University of Oxford
Helen Atherton	h.atherton@warwick.ac.uk	0000-0002-7072-1925	Professor of Primary Care Research	Unit of Academic Primary Care, Warwick Medical School
Jennifer Bostock	jenniferlbostock@icloud.com	0000-0001-9261-9350	Lay PPIE Lead	N/A
Nadia Cross	n.p.cross@soton.ac.uk	0000-0002-4148-7180	Trial Manager	Primary Care Research Centre, School of Primary Care, Population Science, and Medical Education, University of Southampton
Emma Teasdale	e.j.teasdale@soton.ac.uk	0000-0001-9147-193X	Qualitative Research Fellow	School of Psychology and Primary Care Research Centre, School of Primary Care, Population Science, and Medical Education, University of Southampton
Sebastien Pollet	sebastien.pollet@soton.ac.uk	0000-0001-9924-9225	Research Fellow	School of Psychology and Primary Care Research Centre, School of Primary Care,

For peer review only

				Population Science, and Medical Education, University of Southampton
Rachel Dewar-Haggart	r.v.dewar-haggart@soton.ac.uk	0000-0002-3757-1152	Qualitative Research Fellow	School of Psychology and Primary Care Research Centre, School of Primary Care, Population Science, and Medical Education, University of Southampton
Jacqui Nuttall	j.nuttall@soton.ac.uk	0000-0002-5826-2594	Head of Trial Management (Non Cancer)	Southampton Clinical Trials Unit, University of Southampton and University Hospital Southampton NHS Foundation Trust
Nazrul Islam	nazrul.islam@soton.ac.uk	0000-0003-3982-4325	Associate Professor of Epidemiology and Medical Statistics	Primary Care Research Centre, School of Primary Care, Population Science, and Medical Education, University of Southampton
Paul H Lee	paul.h.lee@soton.ac.uk	0000-0002-5729-6450	Associate Professor Medical Statistics	Southampton Clinical Trials Unit, University of Southampton and University Hospital Southampton NHS Foundation Trust
Taeko Becque	t.f.becque@soton.ac.uk	0000-0002-0362-3794	Senior Clinical Trials Statistician	Primary Care Research Centre, School of Primary Care, Population Science, and Medical Education, University of Southampton
Amy Herbert	amy.herbert@bristol.ac.uk	0009-0008-6109-6006	Clinical Studies Officer	Centre of Academic Primary Care, Bristol Medical School, University of Bristol
Michelle Robinson	m.e.robinson@keele.ac.uk	0000-0002-2266-8250	Research Assistant	Keele School of Medicine, Keele University

Word Count

Word count = 4029

Acknowledgements and Funding

Funding: National Institute for Health Research (NIHR) School for Primary Care Research grant (project number 563). The Primary Care Research Centre, University of Southampton is a member of the NIHR School for Primary Care Research and supported by NIHR Research funds. Service support costs will be paid by the CRN. CDM is funded by the National Institute for Health Research (NIHR) Collaborations for Leadership in Applied Health Research and Care West Midlands and the NIHR School for Primary Care Research. The development of the EMPathicO e-learning tool was funded by NIHR School for Primary Care Research grant (project number 389). The EMPathicO e-learning tool was developed using LifeGuide software, which was partly funded by the National Institute for Health Research Southampton Biomedical Research Centre (BRC). The views expressed are those of the authors and not necessarily those of the NIHR or the Department of Health and Social Care.

The study sponsor (University of Southampton) and funders (NIHR SPCR) have no role in study design; collection, management, analysis, and interpretation of data; writing of the report; or the decision to submit the report for publication.

Competing Interests

All authors have completed the ICMJE uniform disclosure form at http://www.icmje.org/disclosure-of-interest/ and declare: financial support for the submitted work from the NIHR; CDM is Director of the NIHR

For peer review only

School for Primary Care Research; HA has received research grants from NIHR and Research Council of Norway, payment for delivering lecture to GPs in training about remote consultations, travel expenses to attend Scientific Foundation Board meeting; HA is chair of a steering committee at University of Leeds, member of advisory boards at Imperial College London and University of Manchester, and vice-chair of the Scientific Foundation Board Royal College of General Practitioners; HA is Officer at Prof Andrew Beggs Ltd. No other relationships or activities that could appear to have influenced the submitted work.

For peer review only

For peer review only - http://bmjopen.bmj.com/site/about/guidelines.xhtml

Abstract

Introduction

Effective communication can help to optimise healthcare interactions and patient outcomes. However, few interventions have been tested clinically or subjected to cost-effectiveness analysis or are sufficiently brief and well-described for implementation in primary care. This paper presents the protocol for determining the effectiveness and cost-effectiveness of a rigorously developed brief eLearning tool, EMPathicO, among patients with and without musculoskeletal pain.

Methods and Analysis

A cluster randomised controlled trial in GP surgeries in England and Wales serving patients from diverse geographic, socio-economic, and ethnic backgrounds. GP surgeries randomised (1:1) to receive EMPathicO e-learning immediately, or at trial end. Eligible practitioners (e.g., GPs, physiotherapists, nurse practitioners) are involved in managing primary care patients with musculoskeletal pain. Patient recruitment managed by practice staff and researchers. Target recruitment is 840 adults with and 840 without musculoskeletal pain consulting face-to-face, by telephone or video. Patients complete web-based questionnaires at pre-consultation baseline, 1-week and 1-, 3- and 6-months later. Two patient-reported primary outcomes – pain intensity and patient enablement. Cost-effectiveness considered from NHS and societal perspectives. Secondary and process measures include practitioner patterns of use of EMPathicO, practitioner-reported self-efficacy/intentions, and patient-reported: symptom severity, quality of life, satisfaction, perceptions of practitioner empathy and optimism, treatment expectancies, anxiety, depression, continuity of care. Purposive sub-samples of patients, practitioners, and practice staff take part in up to two qualitative semi-structured interviews.

Ethics Approval and Dissemination

Approved by South Central – Hampshire B Research Ethics Committee on 1.7.22 and Heath Research Authority and Health and Care Research Wales on 6.7.22 (REC reference 22/SC/0145; IRAS project ID 312208). Results will be disseminated via peer-reviewed academic publications, conference presentations and patient and practitioner outlets. If successful, EMPathicO could quickly be made available at low cost to primary care practices across the country.

Registration

ISRCTN18010240 registered 15 September 2022.

Keywords

Primary Health Care; Empathy; Optimism; Health Communication; Digital Technology; Clinical Trial Protocol

Article Summary

- Assessment of a brief online learning package which is evidence and theory-based and was rigorously developed with primary care clinicians.

For peer review only - http://bmjopen.bmj.com/site/about/guidelines.xhtml

- Practitioners (e.g., GPs, Physios, Nurses) consult as usual without needing to identify or consent patients within the consultation, as patient recruitment is done by administrative staff.
- Focussed on patients with musculoskeletal pain but including other patients as 'all-comers' enables efficient test of relevance to all primary care consultations.
- Feasibility work showed it is not practicable to record consultations in this trial, so there is no direct assessment of changes in practitioner communication behaviours after engaging with the e-learning package.
- 'All-comers' is a large and varied group of patients which enhances generalisability but is not suitably powered to plan sub-group analyses.

For peer review only

Introduction

Approximately 1.7 billion people worldwide have musculoskeletal conditions, which are typically painful, limit peoples' daily lives, and impair quality of life.[1] Musculoskeletal conditions including back, hip, knee and neck pain are commonly managed in primary care,[2-4] where patient-centred care, including excellent practitioner-patient communication, is an international priority.[5-7] In the UK, people with musculoskeletal conditions may be seen in primary care by GPs, practice nurses, physiotherapists, and other allied healthcare professionals.

Regardless of which treatment, therapy, or other intervention a patient receives, effective practitioner-patient communication can reduce symptoms and enhance quality of life, adherence to and satisfaction with care, producing benefits comparable to many pharmaceutical interventions.[8-10] Sub-optimal communication can lead to missed opportunities for benefit, worse quality of life and symptom management, unwanted prescriptions and non-adherence;[11 12] unnecessary economic costs;[12] deviation from guideline-recommended treatment;[13] and increased complaints and litigation.[14 15] Despite communication skills being taught in medical and allied health professional training, patients still report dissatisfaction with practitioner-patient communication,[16 17] the extent to which patients rate their practitioners as being empathic varies widely,[18] and medical students appear to exhibit broadly stable or declining levels of empathy during their degrees.[19 20] The need to enhance and expand communication skills is particularly pertinent since the COVID pandemic forced rapid introduction of remote consultations, bringing new opportunities and challenges for patients and staff not specifically trained to consult in this way.[21]

We focus on the communication of clinical empathy and positive messages within primary care consultations. Clinical empathy and positive messages are not routinely reliably optimised in clinical care but can have statistically and likely clinically significant effects on pain, patient satisfaction, and other outcomes with no evidence of adverse effects.[22] Our intervention planning determined that enhancing practitioners' communication of clinical empathy and realistic optimism was feasible, measurable, and likely to have significant impact.[23 24] Even brief interventions can improve communication skills, including interventions concentrating on empathy skills such as active listening and expressing warmth at appropriate times[25-27] which take no additional time in the consultation.[27 28] However, few interventions have been tested clinically for effects on patients' health,[29] have been subjected to formal cost-effectiveness evaluations,[30] or are sufficiently brief and well-described to facilitate implementation in the current primary care climate. Our work aims to address these limitations. We are evaluating the effects on patients' health of brief, evidence-based, online training to enhance practitioners' communication of clinical empathy and realistic optimism within everyday clinical consultations ("EMPathicO").

Aims and Objectives

The primary objective is to determine EMPathicO's effects on (a) patient-reported pain and (b) patient enablement via repeated measures over 6 months following the index consultation, in patients presenting with musculoskeletal pain, compared to usual care control.

Secondary objectives are:

- To estimate EMPathicO's cost-effectiveness and effects on patient-reported quality of life and other secondary outcomes, over 6 months from index consultation, in patients with musculoskeletal pain.
- To test hypothesised mechanisms of action.

For peer review only - http://bmjopen.bmj.com/site/about/guidelines.xhtml

- To explore EMPathicO's potential for implementation, by:
    - Determining EMPathicO's effects on patient enablement, patient-reported quality of life and other secondary outcomes over 6 months from index consultation, in patients ineligible for the musculoskeletal pain group (i.e., presenting with other symptoms and/or very low levels of musculoskeletal pain, hereafter referred to as 'all-comers').
    - Identifying opportunities, barriers, and solutions for widespread implementation and impact, using the RE-AIM framework to explore EMPathicO's Reach, Effectiveness, Adoption, Implementation, and Maintenance. [31 32]

Methods and Analysis

This protocol reported in accordance with the Standard Protocol Items: Recommendations for Interventional Trials (SPIRIT) checklist (online supplementary material file 1).[33]

Patient and Public Involvement and Engagement (PPIE)

To ensure our work engages and is relevant to patients, we have worked with patients and members of the public throughout developing EMPathicO and this protocol. We continue working closely with our PPIE lead (JB, member of trial management group) and panel of public contributors from diverse backgrounds. Our panel meet bimonthly and contribute to specific activities including refining patient-facing documents and procedures, training qualitative interviewers, and interpreting data. The PPIE lead and group members have lived experience of musculoskeletal conditions as patients or carers.

Design

A cluster-randomised controlled parallel group superiority trial in primary care, with embedded qualitative and mixed methods process and implementation analyses.

Cluster randomisation was chosen because randomising individual practitioners risks cross-contamination within practices where practitioners share knowledge and patients; randomising individual patients risks contamination because practitioners cannot switch on/off communication skills in different consultations.

General practices constitute the clusters; they are randomised 1:1 EMPathicO: control. Randomisation is stratified (see below). All eligible practitioners within clusters are encouraged to undertake EMPathicO training (intervention) or consult patients as usual (control). The control was chosen to enable pragmatic assessment of benefits and costs of adding EMPathicO training to usual care.

Two groups of patients are recruited. The musculoskeletal group comprises patients consulting participating practitioners about musculoskeletal pain. The 'all-comer' group comprises patients consulting about symptoms other than musculoskeletal pain (or reporting very low levels of musculoskeletal pain). At pre-consultation baseline and repeatedly up to 6 months later patients complete questionnaires assessing pain, enablement, and secondary outcomes.

For peer review only - http://bmjopen.bmj.com/site/about/guidelines.xhtml

Setting

General practices in England and Wales, recruited and supported by three recruitment hubs – Southampton, Keele, and Bristol.

Target population

GP Practice Eligibility Criteria

Eligible: NHS general practices in England and Wales.

Excluded: Practices involved in intervention development/feasibility work (18 from Wessex, 5 from West Midlands), practices where clinical members of the Trial Management Group/Trial Steering Committee see patients.

Practitioner Eligibility Criteria

Eligible: practitioners working within participating GP surgeries and seeing patients with musculoskeletal pain (e.g., GPs, Practice Nurses, Physiotherapists).

Excluded: Practitioners unwilling to undertake the intervention/trial procedures.

Patients with Musculoskeletal Pain Eligibility Criteria

For the musculoskeletal pain group, eligible patients are adults (18+); verbally consulting a participating practitioner about new, recurrent, or ongoing musculoskeletal pain (e.g. back, hip, knee, neck pain - consistent with ICD-11's diseases of the musculoskeletal system[34]); reporting average pain in the last week as 4 or more on numerical rating scale at baseline (0 = no pain; 10 = pain as bad as you can imagine); consulting face-to-face , telephone, or videoconference; able to give informed consent. The first consultation is the 'index' consultation, an initial triage interaction does not constitute an 'index' consultation. People without English as a first language are eligible, interpreters are available to support access to trial paperwork and patient-reported measures; informal interpreters (e.g., family) may also support.

Excluded: patients consulting solely in written forms (e.g., e-consult/email); pain caused by malignancy; unable to consent or to complete questionnaires (e.g., severe mental illness or distress, terminal illness); already enrolled in the trial (i.e., from a previous consultation).

All-Comer Patients Eligibility Criteria

For the all-comers group, eligible patients are adults (18+); verbally consulting a participating practitioner about something other than musculoskeletal pain or consulting for musculoskeletal pain and rating average pain in last week as less than 4 at baseline; able to give informed consent.

Excluded: As for patients with musculoskeletal pain.

Interventions

EMPathicO e-Learning Package

EMPathicO is an evidence-based theoretically-grounded digital e-learning package for practitioners routinely seeing patients frontline in primary medical care, including GPs, nurse practitioners and first-

For peer review only - http://bmjopen.bmj.com/site/about/guidelines.xhtml

contact physiotherapists.[24] EMPathicO helps practitioners enhance their communication of clinical empathy and realistic optimism, is consistent with major consultation models including 'ICE' (Ideas, Concerns and Expectations),[35] and incorporates behaviour change techniques. The brief interactive e-learning modules can be completed separately or together in less than 75 minutes and cover clinical empathy, realistic optimism, tailoring empathy and optimism for patients with osteoarthritis (a common cause of musculoskeletal pain), evaluating one's own consultations, and goal-setting (Figure 1). EMPathicO was developed using LifeGuide open-source software for creating online interventions for health care, health promotion and training.[36]

---Insert Figure 1 Here---

The systematic process of developing EMPathicO using the person-based approach[37] involved multiple literature reviews, behavioural analysis, and extensive iterative qualitative research.[38-44] This work all contributed to the underpinning logic model (Figure 2).[24]

---Insert Figure 2 Here---

Control: Usual Care

Practitioners in practices randomised to usual care control do not receive training and are asked to consult as usual. They are offered access to EMPathicO after all patient recruitment and follow-up is completed.

Concomitant Interventions

All practitioners are discouraged from undertaking additional communication skills training during the study and must self-report any that does occur.

Recruitment

GP Practice Recruitment

Practices are recruited with local Clinical Research Network (CRN) support, seeking practices of different sizes (small-large) and locations (urban, rural) and those serving populations in areas of higher deprivation and greater ethnic diversity.

Practitioner Recruitment

Practitioners within participating practices are recruited by that practice's lead for this study (the local PI) with support from the trial team and materials including an infographic and one-minute video explaining the study.

Patient Recruitment

Patient recruitment methods are tailored to suit individual practices' appointment booking systems. For patients with prebooked or same-day appointments, practices text, email, or post a brief invitation and link to the patient-facing study website up to 1 week before their consultation. Practices screen potential

For peer review only - http://bmjopen.bmj.com/site/about/guidelines.xhtml

invitees for initial eligibility before sending invitations. Practices may display a poster in practice and/or on their website. Reception staff may introduce the study to patients attending in-person. Patients email or phone the patient-facing research team with questions.

Practices follow their usual procedures for contacting non-English speakers to invite them to take part e.g., contacting a designated friend, relative or support worker, arranging an interpreter, or adding a sentence in the patient's own language on the initial study invitation.

The patient-facing study website is hosted on Qualtrics and shows the full study invitation and patient information sheet (PIS) (in languages requested by practices). After reading the PIS, patients complete a brief screening questionnaire, online consent and baseline measures. Online supplementary file 2 contains PIS and consent forms.

Sample size

Patients with Musculoskeletal Pain Sample Size

The minimum clinically important difference in the pain primary outcome is approximately one point,[45] standard deviation 3.3, consistent with a standardised effect size of 0.3. For 90% power, alpha of 0.025 to allow for two primary outcomes, and a correlation between the 4 repeated measures of 0.7, a sample size of 214 per group is required. We assume a conservative ICC of 0.03, at the upper 75% of what has been observed in previous primary care trials.[46] Assuming 20 patients per practice gives a design effect of 1.57. Allowing for 20% loss to follow up gives a total sample size of (214*2*1.57)/0.8=840 participants to be recruited from 42 practices.

'All-Comer' Patients Sample Size

Recruiting 840 all-comers will give 90% power (based on alpha and ICC as per the musculoskeletal group above) to detect a standardised effect size of 0.3 in the enablement primary outcome, equivalent to a difference of 0.36 points (assuming SD=1.2[47]).

Updated sample size calculation

Participants are being recruited from 53 practices rather than 42 practices as originally planned, which reduces the average cluster size. Assuming 14 patients per practice gives a design effect of 1.39. Under the same assumptions as above, the total sample size is (214*2*1.39)/0.8=744 participants.

Outcomes

Questionnaires, Data Collection and Participant Retention

Online supplementary file 3 summarises outcome and process variables, measurement timings, and questionnaire measures. We considered core outcome sets, questionnaire properties (e.g., validity, reliability, length), and acceptability to participants when choosing specific measures.

Patient-reported measures are completed on web-based questionnaires hosted on Qualtrics (Qualtrics, Provo, UT); to support inclusive access patients may request an interpreter and/or paper versions. £10 vouchers are sent at 1-month and 6-month follow-ups to incentivize completion.

For peer review only - http://bmjopen.bmj.com/site/about/guidelines.xhtml

Practitioner-reported measures are completed on LifeGuide[36] (measures completed by intervention group only) and Qualtrics (measures completed by all practitioners).

For practitioners and patients, automated follow-up emails are sent to non-responders at all timepoints. Researchers personally contact persistent non-responders who haven't withdrawn and offer to resend questionnaires or complete primary outcomes by telephone.

Primary Outcomes

For the musculoskeletal pain group, the two primary outcomes are pain intensity and patient enablement, each analysed over 6 months using a repeated measures approach. Pain intensity is the severity of pain sensation and is included in core outcome sets for chronic pain,[48-50] OA,[51] and low back pain.[52 53] Patient enablement refers to patients' feelings, after a consultation, of confidence and empowerment to cope with their symptoms, to keep healthy and to help themselves. Our PPIE work highlighted enablement as at least as important as pain. Two primary outcomes help capture more holistic effects on patients' health. The outcomes will be reported separately and our PPIE and embedded qualitative work will help explore, interpret and explain how they relate to each other.

For the all-comers group, patient enablement is the single primary outcome. Pain intensity is measured as a secondary outcome if pain is present.

Pain Intensity

Pain intensity is measured as average pain in the last week using the 4-item pain intensity subscale from the Brief Pain Inventory (BPI).[54]

Patient Enablement

The 6-item Patient Enablement Index (PEI) captures patients' feelings, after a consultation, of confidence and empowerment to cope with their symptoms, to keep healthy and to help themselves.[55] To increase sensitivity, versions with more response options than the original four (much better/never/same or less/not applicable) have been reported.[56-58] Following our feasibility study we use a modified 7-point agree-disagree Likert response scale with a Not Applicable option.

Secondary Outcomes

Symptom Severity and Global Impression of Change

Overall perceptions of symptom severity and change are important for musculoskeletal patients given the high prevalence of multi-morbid conditions and for all-comers because they apply to any condition and provide a symptom-focused pre-consultation baseline. Two single item 7-point[59] measures of Patient Global Impression of Symptom Severity and Patient Global Impression of Change are collected.[60]

Patient Satisfaction

The version of the 21-item Medical Interview Satisfaction Scale[61] (MISS) adapted and revalidated for UK primary care[62] is used to measure patient satisfaction with the consultation.

Pain Interference

Pain interference is measured with the 7-item pain interference scale from the BPI[54].

For peer review only - http://bmjopen.bmj.com/site/about/guidelines.xhtml

Health-Related Quality of Life

Health status is measured using the 5-item EQ-5D-5L and the EQ-VAS.[63]

Health Economics Outcomes

Cost effectiveness will be assessed from NHS and societal perspectives including personal expenses and productivity over 6 months. Utility values will be estimated from EQ-5D-5L scores using the NICE-recommended approach at the time of analysis. Quality-adjusted life-years will be estimated by combining utility values, with length of time in each health state, using the area under the curve approach.[63-65] The 5-item ICECAP-A, which was designed to capture broader aspects of quality-of-life and has been found to complement the EQ-5D in economic evaluations, is also collected.[66 67]

Practitioner time spent on EMPathicO training is captured by LifeGuide. Resource-use data is collected using ModRUM[68] (patient self-reported healthcare utilization) and bespoke questions (costs outside the healthcare sector e.g., personal expenses). The Work Productivity and Activity Impairment Questionnaire: General Health is used to collect information on productivity, including time off work.[69] NHS resources include primary, community and secondary care, and prescribed medications; they will be valued using the national unit costs.[70-72] Personal expenses will be presented as reported. Sick leave from employment will be valued using Annual Survey of Hours and Earnings.[73]

Process Variables and Covariates

Potential mediators and moderators of intervention effects on pain, specified in the logic model, are included as process variables. Practitioner-reported self-efficacy, outcome expectancy, and intentions for conveying empathy and optimism in consultations are assessed using bespoke items developed in our feasibility work based on standard item stems, relevant guidelines and theory.[74-77] They demonstrated acceptable internal consistency (Cronbach's alphas ranged 0.69-0.98) and were fully completed by practitioners (n=11).

Intervention usage data captured on LifeGuide includes, for each practitioner-participant, time spent on (different sections of) the intervention and patterns of access.

Patient perceptions of practitioner clinical empathy are assessed using the 10-item CARE[78] used extensively in UK primary care settings to assess patient perceptions of clinical empathy. Patient perceptions of practitioner response expectancies are assessed using a bespoke single item tested in our feasibility study. Patient treatment outcome expectancies are measured using the 15-item 6-subscale, Treatment Expectation Questionnaire (TEX-Q).[79] Patient anxiety and depression are assessed using the 7-item subscales from the Hospital Anxiety and Depression Scale (HADS).[80 81] Continuity of care is assessed using the 9-item Patient-Doctor Depth of Relationship Scale,[82] modified for non-doctor practitioners.

Practitioner characteristics collected are age, gender, ethnicity, years qualified, profession. Practice-level data collected from the practice and supplemented with data from national general practice profiles (National General Practice Profiles - Data – OHID, phe.org.uk) are: list size, deprivation score, staffing.

Patient characteristics collected are age, gender, ethnicity, postcode (for calculating index of multiple deprivation, IMD), reason(s) for consulting (coded using the ICPC-2), comorbidities, and index consultation modality.

For peer review only - http://bmjopen.bmj.com/site/about/guidelines.xhtml

Qualitative Interviews

A subsample of patients (up to n=45 with musculoskeletal pain and n=45 all-comers) and practitioners (up to n=40) take part in qualitative semi-structured telephone interviews. Participants are purposively sampled to capture diversity in index-consultation mode (telephone/video/face-to-face), ethnicity, age, gender, baseline pain severity. Participants are interviewed twice each, to explore short-term and longer-term implementation of EMPathicO skills (practitioners) and experiences of the index and subsequent consultations (patients). Practitioners are interviewed after (1) patient recruitment and (2) follow-up is completed at their practice. Patients are interviewed within approximately 7-14 days of their index consultation and again approximately 6 months later. Topic guides comprising open-ended questions and prompts are used flexibly and modified iteratively as necessary to explore emerging avenues of inquiry within scope of the trial. Field notes are taken, interviews are transcribed verbatim, identifying details are replaced (e.g., using pseudonyms), and transcripts are checked and imported to NVivo (Lumivero, Denver, CO) for analysis.

Timelines

Tables 1 and 2 show practitioner and patient timelines for enrolment, questionnaires, and interviews.

---Insert Tables 1 and 2 Here ---

Assignment of Interventions

Sequence Generation, Allocation Concealment and Implementation

A computer-generated allocation sequence is used with random block sizes of 4 and 6. Blocks are stratified by practice-level high/low deprivation (IMD 1-5 / IMD 6-10) and large/small practice size (list size>7900 / <7900; 7900 = median practice list size in England). The allocation sequence is implemented using the randomisation function in LifeGuide and is not visible to users. The trial manager (or their delegate) inputs each eligible practice to the randomisation function on LifeGuide which then displays the allocation. Practitioners and patients can withdraw from the study without giving a reason, but they cannot request modification to their allocated intervention.

Blinding

Patients and the trial statistician are masked to intervention allocation. Efforts are made to mask researchers supporting patient data collection to intervention allocation. Efforts are made to mask practitioners to which patients are taking part. In the unlikely event that patient unblinding is deemed necessary for patient care this will be done by the general practice and notified to the research team.

Data Analysis

Data Management

Web-based questionnaire data stored securely on Qualtrics servers (see https://www.qualtrics.com/security-statement/). Questionnaire data collected by telephone or paper entered into Qualtrics by one researcher and checked for accuracy by a second researcher.

For peer review only - http://bmjopen.bmj.com/site/about/guidelines.xhtml

Personal data stored on a secure server at University of Southampton in compliance with General Data Protection Regulations and the Data Protection Act 2018.

Statistical Methods

Musculoskeletal and all-comers groups will be analysed separately. For the two primary outcomes, a linear mixed model will use all the observed data, and implicitly assumes that missing outcome scores are missing at random given the observed data. The primary analyses for the BPI and PEI scores will be performed using a generalized linear mixed model (GLMM) framework with observations at 3 days, 1-. 3-, and 6-months (level 1) nested in participants (level 2) and participants nested in practices (level 3). Unadjusted results will be reported as well as results adjusting for baseline values, stratification variables and other covariates as appropriate. As there may not be a constant treatment effect over time, a treatment/time interaction will be modelled and included if significant, with time treated as a random effect. An unstructured covariance matrix will be used. For secondary outcomes, the analyses will use a similar modelling approach, with mixed logistic/linear regression models as appropriate, a random effect for practice, controlling for baseline values, stratification variables and potential confounders. No formal pre-planned subgroup analyses.

Intention to treat analysis (as randomised) will be undertaken regardless of any practice-level non-adherence to the intervention. All available data will be used, with a sensitivity analysis using multiple imputation if appropriate. Linear mixed models and multiple imputation both assume the data are missing at random, therefore sensitivity analyses to data missing not at random will also be explored. A full and detailed statistical analysis plan will be developed prior to final trial analysis and approved by Trial Steering Committee.

Interim analyses of outcomes are deemed unnecessary in this low-risk trial.

Health Economic Analysis

An NHS perspective will be taken in the primary analysis; a wider perspective is taken in secondary analyses including impacts on patients and productivity. Analysis will be intention to treat. Relevant covariates, including baseline EQ-5D-5L, potentially skewed data and the cluster design will be accounted for using appropriate regression models.[65] Cost-consequences will tabulate costs from each perspective to a range of outcomes. Cost-effectiveness will be estimated in a cost-utility analysis combining QALYs and NHS costs. The incremental net monetary benefit statistic will be presented at standard NICE thresholds and if appropriate, incremental cost-effectiveness ratios will be estimated. Uncertainty will be addressed by bootstrapping, plotting cost-effectiveness acceptability curves and in sensitivity analyses.

Process Analysis

A process analysis will focus on mechanisms of impact and test hypotheses derived from the logic model about relationships among variables, including mediators and moderators.[110] Intervention usage data, captured by LifeGuide, will be incorporated using the AMUsED framework for Analyzing and Measuring Usage and Engagement Data.[111]

Qualitative and Mixed Methods Analysis

EMPathicO's potential impact post-trial will be evaluated by using the RE-AIM framework to explore Reach, Effectiveness, Adoption, Implementation, and Maintenance. [31 32] Drawing on data from the main trial, the all-comers group and the qualitative interviews we will assess EMPathicO against the RE-AIM components using the approaches described in Table 3.

For peer review only - http://bmjopen.bmj.com/site/about/guidelines.xhtml

---Insert Table 3 Here---

Ethics and Dissemination

Safety, Adverse Events, and Insurance

This trial is classed as low risk following a risk assessment and there are no provisions for post-trial care. The team do not expect any adverse events (untoward medical occurrence in a trial participant) or Serious Adverse Events (that results in death, is life-threatening, requires hospitalisation or prolongation of existing hospitalisation, results in persistent or significant disability or incapacity, consists of a congenital anomaly or birth defect, or other medically important condition). However, adverse events are being collected (primarily via self-report), recorded and reported where necessary in accordance with good clinical practice and the requirements of the research ethics committee, sponsor, and trial steering committee.

Individual practitioners are responsible for maintaining appropriate cover with a medical defence organisation. University of Southampton insurance may also apply where the cause of harm was not due to clinical negligence.

Approvals, Oversight and Monitoring

The sponsor is the University of Southampton. Approval was received from South Central – Hampshire B Research Ethics Committee on 1.7.22 and the Heath Research Authority and Health and Care Research Wales on 6.7.22 (REC reference 22/SC/0145; IRAS project ID 312208). Protocol amendments are submitted for approval as required to the study sponsor and ethics committee and notified where necessary to all those concerned.

The Trial Steering Committee (TSC) provides trial oversight and advice through its independent Chairperson to the Trial Management Group and the funder on all aspects of the trial. The TSC assumes responsibilities of the Data Monitoring Committee and reviews information on the progress and accruing data; online supplementary file 4 presents the TSC Charter; online supplementary file 5 presents stopping criteria). Annual and interim progress reports submitted to the funder.

Dissemination

Patient recruitment commenced on 16.11.2022 and is ongoing at the time of manuscript submission. Results will be communicated to participants and disseminated to academic, practitioner, and public audiences via peer-review journal articles, conferences, and other appropriate formats e.g. blogs. Our public collaborators will co-lead dissemination activities. Results will be reported in accordance with CONSORT guidelines extensions for cluster-randomised trials[83] and trials of non-pharmacological interventions,[84] and the American Psychological Association Journal Article Reporting Standards for qualitative (JARS-QUAL) and mixed methods (JARS MMARS) research.[85] We will adhere to the ICMJE (https://www.icmje.org/) criteria for authorship and use the CRediT taxonomy (https://credit.niso.org/). Online supplementary file 6 summarises data access plans.

For peer review only - http://bmjopen.bmj.com/site/about/guidelines.xhtml

References

1. Cieza A, Causey K, Kamenov K, et al. Global estimates of the need for rehabilitation based on the Global Burden of Disease study 2019: a systematic analysis for the Global Burden of Disease Study 2019. *The Lancet* 2020;396(10267):2006-17. doi: 10.1016/S0140-6736(20)32340-0
2. Jordan KP, Kadam UT, Hayward R, et al. Annual consultation prevalence of regional musculoskeletal problems in primary care: an observational study. *BMC Musculoskeletal Disorders* 2010;11(144)
3. Ruairi K. The prevalence of musculoskeletal presentations in general practice: an epidemiological study. *Br J Gen Pract* 2020;70(suppl 1):bjgp20X711497. doi: 10.3399/bjgp20X711497
4. Yu D, Missen M, Jordan KP, et al. Trends in the Annual Consultation Incidence and Prevalence of Low Back Pain and Osteoarthritis in England from 2000 to 2019: Comparative Estimates from Two Clinical Practice Databases. *Clinical Epidemiology* 2022;14(null):179-89. doi: 10.2147/CLEP.S337323
5. Van Lerberghe W. The world health report 2008: primary health care: now more than ever: World Health Organization 2008.
6. NICE NIfHaCE. Musculoskeletal Conditions Overview [Available from: https://pathways.nice.org.uk/pathways/musculoskeletal-conditions accessed 02.06.2021.
7. Lin I, Wiles L, Waller R, et al. What does best practice care for musculoskeletal pain look like? Eleven consistent recommendations from high-quality clinical practice guidelines: systematic review. *Br J Sports Med* 2020;54(2):79. doi: 10.1136/bjsports-2018-099878
8. Suarez-Almazor ME, Looney C, Liu Y, et al. A randomized controlled trial of acupuncture for osteoarthritis of the knee: Effects of patient-provider communication. *Arthritis Care Res* 2010;62(9):1229-36.
9. Haskard Zolnierek KB, DiMatteo MR. Physician communication and patient adherence to treatment: A meta-analysis. *Med Care* 2009;47(8)
10. Dambha-Miller H, Cooper AJM, Kinmonth AL, et al. Effect on cardiovascular disease risk factors of interventions to alter consultations between practitioners and patients with type 2 diabetes: A systematic review and meta-analysis of trials in primary care. *Health Expect* 2017;20(6):1218-27. doi: 10.1111/hex.12546 [published Online First: 2017/02/28]
11. Barry CA, Bradley CP, Britten N, et al. Patients' unvoiced agendas in general practice consultations: qualitative study. *Br Med J* 2000;320:1246-50.
12. Thorne SE, Bultz BD, Baile WF. Is there a cost to poor communication in cancer care?: a critical review of the literature. *Psychooncology* 2005;14(10):875-84. doi: 10.1002/pon.947
13. Moffat M, Cleland J, van der Molen T, et al. Poor communication may impair optimal asthma care: a qualitative study. *Fam Pract* 2007;24(1):65-70. doi: 10.1093/fampra/cml062
14. Stelfox HT, Gandhi TK, Orav EJ, et al. The relation of patient satisfaction with complaints against physicians and malpractice lawsuits. *The American Journal of Medicine* 2005;118(10):1126-33. doi: http://dx.doi.org/10.1016/j.amjmed.2005.01.060
15. Pincock S. Poor communication lies at heart of NHS complaints, says ombudsman. *BMJ* 2004;328(7430):10.
16. Nielsen M, Foster M, Henman P, et al. ‘Talk to us like we’re people, not an X-ray’: the experience of receiving care for chronic pain. *Australian Journal of Primary Health* 2013;19(2):138-43. doi: http://dx.doi.org/10.1071/PY11154
17. Teh CF, Karp JF, Kleinman A, et al. Older People's Experiences of Patient-Centered Treatment for Chronic Pain: A Qualitative Study. *Pain Medicine* 2009;10(3):521-30.
18. Howick J, Steinkopf L, Ulyte A, et al. How empathic is your healthcare practitioner? A systematic review and meta-analysis of patient surveys. *BMC Med Educ* 2017;17(1):136. doi: 10.1186/s12909-017-0967-3 [published Online First: 2017/08/22]

For peer review only - http://bmjopen.bmj.com/site/about/guidelines.xhtml

19. Quince TA, Parker RA, Wood DF, et al. Stability of empathy among undergraduate medical students: a longitudinal study at one UK medical school. *BMC Med Educ* 2011;11:90. doi: 10.1186/1472-6920-11-90 [published Online First: 2011/10/27]
20. Costa-Drolon E, Verneuil L, Manolios E, et al. Medical Students' Perspectives on Empathy: A Systematic Review and Metasynthesis. *Acad Med* 2021;96(1):142-54. doi: 10.1097/acm.0000000000003655 [published Online First: 2020/08/10]
21. Murphy M, Scott LJ, Salisbury C, et al. Implementation of remote consulting in UK primary care following the COVID-19 pandemic: a mixed-methods longitudinal study. *Br J Gen Pract* 2021;71(704):e166. doi: 10.3399/BJGP.2020.0948
22. Howick J, Moscrop A, Mebius A, et al. Effects of empathic and positive communication in healthcare consultations: a systematic review and meta-analysis. *J R Soc Med* 2018;111(7):240-52. doi: 10.1177/0141076818769477 [published Online First: 2018/04/20]
23. Michie S, van Stralen MM, West R. The behaviour change wheel: A new method for characterising and designing behaviour change interventions. *Implementation Science* 2011;6(1):42. doi: 10.1186/1748-5908-6-42
24. Smith KA, Vennik J, Morrison L, et al. Harnessing placebo effects in primary care: Using the person-based approach to develop an online intervention to enhance practitioners' communication of clinical empathy and realistic optimism during consultations. *Frontiers in Pain Research* 2021;2 doi: 10.3389/fpain.2021.721222
25. Little P, Everitt H, Williamson I, et al. Observational study of effect of patient centredness and positive approach on outcomes of general practice consultations. *Br Med J* 2001;323 908-11.
26. Little P, White P, Kelly J, et al. Verbal and non-verbal behaviour and patient perception of communication in primary care: an observational study. *Br J Gen Pract* 2015;65(635):e357-e65. doi: 10.3399/bjgp15X685249
27. Little P, White P, Kelly J, et al. Randomised controlled trial of a brief intervention targeting predominantly non-verbal communication in general practice consultations. *Br J Gen Pract* 2015;65(635):e351-e56. doi: 10.3399/bjgp15X685237
28. Griffin SJ, Kinmonth AL, Veltman MWM, et al. Effect on Health-Related Outcomes of Interventions to Alter the Interaction Between Patients and Practitioners: A Systematic Review of Trials. *Annals of family medicine* 2004;2(6):595-608.
29. Dwamena F, Holmes-Rovner M, Gaulden CM, et al. Interventions for providers to promote a patient-centred approach in clinical consultations. *Cochrane Database of Systematic Reviews* 2012;12:Art. No.: CD003267. DOI: 10.1002/14651858.CD003267.pub2.
30. Howick J, Mittoo S, Abel L, et al. A price tag on clinical empathy? Factors influencing its cost-effectiveness. *J R Soc Med* 2020;113(10):389-93. doi: 10.1177/0141076820945272
31. Glasgow RE, Vogt TM, Boles SM. Evaluating the public health impact of health promotion interventions: the RE-AIM framework. *Am J Public Health* 1999;89(9):1322-7. doi: 10.2105/ajph.89.9.1322 [published Online First: 1999/09/04]
32. Glasgow RE, Harden SM, Gaglio B, et al. RE-AIM Planning and Evaluation Framework: Adapting to New

Science and Practice With a 20-Year Review. *Frontiers in Public Health* 2019;7:64. doi: 10.3389/fpubh.2019.00064
33. Chan A-W, Tetzlaff JM, Altman DG, et al. SPIRIT 2013 Statement: Defining Standard Protocol Items for Clinical Trials. *Ann Intern Med* 2013;158(3):200-07. doi: 10.7326/0003-4819-158-3-201302050-00583
34. Organization WH. International Statistical Classification of Diseases and Related Health Problems (ICD). https://www.who.int/standards/classifications/classification-of-diseases, 2021.
35. Whitaker P. Ticking the ICE box: the future of doctor-patient communication in a post-covid world. *BMJ* 2021;373:n870. doi: 10.1136/bmj.n870
36. Yardley L, Osmond A, Hare J, et al. Introduction to the LifeGuide: software facilitating the development of interactive behaviour change internet interventions. . In Adaptive and Emergent Behaviour and

For peer review only - http://bmjopen.bmj.com/site/about/guidelines.xhtml

Complex Systems-Proceedings of the 23rd Convention of the Society for the Study of Artificial Intelligence and Simulation of Behaviour AISB2009.
37. Yardley L, Morrison L, Bradbury K, et al. The person-based approach to intervention development: Application to digital health-related behavior change interventions. *J Med Internet Res* 2015;17(1)
38. Smith KA, Bishop FL, Dambha-Miller H, et al. Improving Empathy in Healthcare Consultations-a Secondary Analysis of Interventions. *J Gen Intern Med* 2020;35(10):3007-14. doi: 10.1007/s11606-020-05994-w [published Online First: 2020/07/14]
39. Howick J, Lyness E, Albury C, et al. Anatomy of positive messages in healthcare consultations: component analysis of messages within 22 randomised trials. *Eur J Pers Cent Healthc* 2019;17:656-64.
40. Budd G, Griffiths D, Howick J, et al. Empathy in patient-clinician interactions when using telecommunication: A rapid review of the evidence. *PEC Innovation* 2022;1:100065. doi: https://doi.org/10.1016/j.pecinn.2022.100065
41. Lyness E, Vennik JL, Bishop FL, et al. Exploring patient views of empathic optimistic communication for osteoarthritis in primary care: a qualitative interview study using vignettes. *BJGP Open* 2021:BJGPO.2021.0014. doi: 10.3399/BJGPO.2021.0014
42. Vennik J, Hughes S, Smith KA, et al. Patient and practitioner priorities and concerns about primary healthcare interactions for osteoarthritis: A meta-ethnography. *Patient Educ Couns* 2022 doi: https://doi.org/10.1016/j.pec.2022.01.009
43. Hughes S, Vennik JL, Smith KA, et al. Clinician views on optimism and empathy in primary care consultations: a qualitative interview study. *BJGP Open* 2022;6(3):BJGPO.2021.0221. doi: 10.3399/BJGPO.2021.0221
44. Vennik J, Hughes S, Lyness E, et al. Patient perceptions of empathy in primary care telephone consultations: A mixed methods study. *Patient Educ Couns* 2023;113:107748. doi: https://doi.org/10.1016/j.pec.2023.107748
45. Dworkin RH, Turk DC, Wyrwich KW, et al. Interpreting the clinical importance of treatment outcomes in chronic pain clinical trials: IMMPACT recommendations. *J Pain* 2008;9(2):105-21. doi: 10.1016/j.jpain.2007.09.005 [published Online First: 2007/12/07]
46. Adams G, Gulliford MC, Ukoumunne OC, et al. Patterns of intra-cluster correlation from primary care research to inform study design and analysis. *J Clin Epidemiol* 2004;57(8):785-94. doi: 10.1016/j.jclinepi.2003.12.013 [published Online First: 2004/10/16]
47. Bishop FL, Smith KA, Vennik J, et al. Feasibility Study of a Novel Online Intervention to Enhance Practitioners' Communication of Clinical Empathy and Realistic Optimism During Primary Care Consultations, 2022, in preparation.
48. Dworkin RH, Turk DC, Farrar JT, et al. Core outcome measures for chronic pain clinical trials: IMMPACT recommendations. *Pain* 2005;113(1-2):9-19.
49. Kroenke K, Krebs EE, Turk D, et al. Core Outcome Measures for Chronic Musculoskeletal Pain Research: Recommendations from a Veterans Health Administration Work Group. *Pain Med* 2019;20(8):1500-08. doi: 10.1093/pm/pny279 [published Online First: 2019/01/08]
50. Dworkin RH, Turk DC, Farrar JT, et al. Core outcome measures for chronic pain clinical trials: IMMPACT recommendations. *PAIN* 2005;113(1):9-19. doi: 10.1016/j.pain.2004.09.012
51. Smith TO, Hawker GA, Hunter DJ, et al. The OMERACT-OARSI Core Domain Set for Measurement in Clinical Trials of Hip and/or Knee Osteoarthritis. *The Journal of Rheumatology* 2019:jrheum.181194. doi: 10.3899/jrheum.181194
52. Chiarotto A, Deyo RA, Terwee CB, et al. Core outcome domains for clinical trials in non-specific low back pain. *Eur Spine J* 2015;24(6):1127-42. doi: 10.1007/s00586-015-3892-3
53. Chiarotto A, Boers M, Deyo RA, et al. Core outcome measurement instruments for clinical trials in nonspecific low back pain. *Pain* 2018;159(3)
54. Keller S, Bann CM, Dodd SL, et al. Validity of the Brief Pain Inventory for use in documenting the outcomes of patients with noncancer pain. *Clin J Pain* 2004;20(5):309-18.

For peer review only - http://bmjopen.bmj.com/site/about/guidelines.xhtml

55. Howie JG, Heaney DJ, Maxwell M, et al. A comparison of a Patient Enablement Instrument (PEI) against two established satisfaction scales as an outcome measure of primary care consultations. *Fam Pract* 1998;15(2):165-71.
56. Morrison LG, Geraghty AWA, Lloyd S, et al. Comparing usage of a web and app stress management intervention: An observational study. *Internet Interventions* 2018;12:74-82. doi: https://doi.org/10.1016/j.invent.2018.03.006
57. Molgaard Nielsen A, Hartvigsen J, Kongsted A, et al. The patient enablement instrument for back pain: reliability, content validity, construct validity and responsiveness. *Health and quality of life outcomes* 2021;19(1):116. doi: 10.1186/s12955-021-01758-0 [published Online First: 2021/04/11]
58. Bedford LE, Yeung MHY, Au CH, et al. The validity, reliability, sensitivity and responsiveness of a modified Patient Enablement Instrument (PEI-2) as a tool for serial measurements of health enablement. *Fam Pract* 2020 doi: 10.1093/fampra/cmaa102
59. Preston CC, Colman AM. Optimal number of response categories in rating scales: reliability, validity, discriminating power, and respondent preferences. *Acta Psychol (Amst)* 2000;104(1):1-15. doi: https://doi.org/10.1016/S0001-6918(99)00050-5
60. Fischer D, Stewart AL, Bloch DA, et al. Capturing the Patient's View of Change as a Clinical Outcome Measure. *JAMA* 1999;282(12):1157-62. doi: 10.1001/jama.282.12.1157
61. Wolf MH, Putnam SM, James SA, et al. The Medical Interview Satisfaction Scale: development of a scale to measure patient perceptions of physician behavior. *J Behav Med* 1978;1(4):391-401. [published Online First: 1978/12/01]
62. Meakin R, Weinman J. The 'Medical Interview Satisfaction Scale' (MISS-21) adapted for British general practice. *Fam Pract* 2002;19(3):257-63. doi: 10.1093/fampra/19.3.257
63. Herdman M, Gudex C, Lloyd A, et al. Development and preliminary testing of the new five-level version of EQ-5D (EQ-5D-5L). *Qual Life Res* 2011;20(10):1727-36. doi: 10.1007/s11136-011-9903-x [published Online First: 2011/04/12]
64. National Institute for Health and Care Excellence. Developing NICE guidelines: the manual. London, UK: National Institute for Health and Care Excellence (NICE) 2014.
65. Manca A, Hawkins N, Sculpher MJ. Estimating mean QALYs in trial-based cost-effectiveness analysis: the importance of controlling for baseline utility. *Health Econ* 2005;14(5):487-96. doi: 10.1002/hec.944 [published Online First: 2004/10/22]
66. Al-Janabi H, Flynn TN, Coast J. Development of a self-report measure of capability wellbeing for adults: the ICECAP-A. *Qual Life Res* 2012;21(1):167-76. doi: 10.1007/s11136-011-9927-2 [published Online First: 2011/05/21]
67. Keeley T, Coast J, Nicholls E, et al. An analysis of the complementarity of ICECAP-A and EQ-5D-3 L in an adult population of patients with knee pain. *Health and quality of life outcomes* 2016;14:36. doi: 10.1186/s12955-016-0430-x [published Online First: 2016/03/05]
68. Garfield K, Husbands S, Thorn JC, et al. Development of a brief, generic, modular resource-use measure (ModRUM): cognitive interviews with patients. *BMC Health Serv Res* 2021;21(1):371. doi: 10.1186/s12913-021-06364-w
69. Reilly MC, Zbrozek AS, Dukes EM. The validity and reproducibility of a work productivity and activity impairment instrument. *Pharmacoeconomics* 1993;4(5):353-65. doi: 10.2165/00019053-199304050-00006 [published Online First: 1993/10/05]
70. NHS England and NHS Improvement. National Schedule of NHS Costs, 2020.
71. Joint Formulary Committee. British National Formulary (online). London: BMJ Group and Pharmaceutical Press.
72. Curtis L, Burns A. Unit Costs of Health & Social Care 2020. University of Kent: PSSRU 2020.
73. Office for National Statistics. Annual Survey of Hours and Earnings 2020. UK: Office for National Statistics, 2020.
74. Bandura A. Guide for constructing self-efficacy scales. In: Urdan T, Pajares F, eds. Self-Efficacy Beliefs of Adolescents: Information Age Publishing 2006:307-37.

For peer review only - http://bmjopen.bmj.com/site/about/guidelines.xhtml

75. Ajzen I. Constructing a theory of planned behavior questionnaire. Online publication. http://people.umass.edu/aizen/pdf/tpb.measurement.pdf. Last accessed 08/11/2023.
76. Renner B, Schwarzer R. Risk and Health Behaviors. Documentation of the Scales of the Research Project: "Risk Appraisal Consequences in Korea" (RACK). http://www.gesundheitsrisiko.de/docs/RACKEnglish.pdf: International University Bremen & Freie Universität Berlin, 2007.
77. Francis JJ, Eccles MP, Johnston M, et al. Constructing questionnaires based on the theory of planned behaviour. A manual for health services researchers. University of Newcastle: Centre for Health Services Research, 2004.
78. Mercer SW, Maxwell M, Heaney D, et al. The development and preliminary validation of the Consultation and Relational Empathy (CARE) measure: an empathy-based consultation process measure. *Fam Pract* 2004;21 699-705.
79. Alberts J, Löwe B, Glahn MA, et al. Development of the generic, multidimensional Treatment Expectation Questionnaire (TEX-Q) through systematic literature review, expert surveys and qualitative interviews. *BMJ Open* 2020;10(8):e036169. doi: 10.1136/bmjopen-2019-036169
80. Bjelland I, Dahl AA, Haug TT, et al. The validity of the Hospital Anxiety and Depression Scale: An updated literature review. *J Psychosom Res* 2002;52(2):69-77.
81. Zigmond AS, Snaith RP. The Hospital Anxiety and Depression Scale. *Acta Psychiatr Scand* 1983;67(6):361-70.
82. Ridd MJ, Lewis G, Peters TJ, et al. Patient-Doctor Depth-of-Relationship Scale: Development and Validation. *The Annals of Family Medicine* 2011;9(6):538. doi: 10.1370/afm.1322
83. Campbell MK, Piaggio G, Elbourne DR, et al. Consort 2010 statement: extension to cluster randomised trials. *BMJ* 2012;345:e5661. doi: 10.1136/bmj.e5661 [published Online First: 2012/09/07]
84. Boutron I, Altman DG, Moher D, et al. CONSORT Statement for Randomized Trials of Nonpharmacologic Treatments: A 2017 Update and a CONSORT Extension for Nonpharmacologic Trial Abstracts. *Ann Intern Med* 2017;167(1):40-47. doi: 10.7326/m17-0046 [published Online First: 2017/06/21]
85. Levitt HM, Bamberg M, Creswell JW, et al. Journal article reporting standards for qualitative primary, qualitative meta-analytic, and mixed methods research in psychology: The APA Publications and Communications Board task force report. *Am Psychol* 2018;73(1):26-46. doi: 10.1037/amp0000151 [published Online First: 2018/01/19]
86. Murray E, Treweek S, Pope C, et al. Normalisation process theory: a framework for developing, evaluating and implementing complex interventions. *BMC Medicine* 2010;8(1):63.

For peer review only

For peer review only - http://bmjopen.bmj.com/site/about/guidelines.xhtml

Tables and Figures

Table 1. Practitioner Timelines

	Allocation		Post-allocation (wk)				On completing patient recruitment	On completing patient follow-up
**TIMEPOINT**	**0**	**+1d**	**1**	**2**	**3-8**	**8**		
**ENROLMENT:**								
Eligibility screen	X							
Informed consent	X							
Site initiation visit	X							
Allocation		X						
**INTERVENTIONS:**								
EMPathicO training								
No training (control)								
**ASSESSMENTS:**								
Demographic and professional characteristics	X							
Self-efficacy for empathy and optimism	X					X		X
Expectations, intentions for EMPathicO skills [1]				x		x		X
Practitioner-reported other training						x		X
Qualitative interview							X	X[1]
**PATIENT RECRUITMENT**								
Prepare invitations								
Recruit patients								

[1] Intervention-arm practitioners only

For peer review only

For peer review only - http://bmjopen.bmj.com/site/about/guidelines.xhtml

Table 2. Patient Timelines

	Enrol	Consultation	Post-consultation			
**TIMEPOINT**	***<-7d***	**0**	***<7d***	***+1m***	***+3m***	***+6m***
**ENROLMENT:**						
Eligibility screen	X					
Informed consent	X					
**ASSESSMENTS:**						
Primary Outcomes						
Pain intensity	X		X	X	X	X
Patient enablement			X	X	X	X
Secondary Outcomes						
Global impression of symptom severity	X		X	X	X	X
Global impression of symptom change			X	X	X	X
Pain interference				X		X
Patient satisfaction			X			
Health economics: EQ-5D & ICECAP-A	X			X		X
Adverse events				X	X	X
Healthcare utilization	X				X	X
Prescribed medications, personal expenses, productivity					X	X
Process Measures						
Perceptions of empathy			X			
Perceptions of optimism			X			
Treatment expectations			X			
Anxiety			X			
Continuity of care			X			
Depression			X			
Sociodemographic characteristics	X					
Health characteristics			X			
Qualitative interview			X			X

For peer review only

Table 3: Qualitative and Mixed Methods Data Analysis to Evaluate Intervention

RE-AIM	Data source	Analysis
**Reach**	Management data	Proportion and characteristics of practitioners and patients taking part. Reasons for declining.
**Effectiveness**	All-comers group	Apply analysis plan from main trial to test intervention effectiveness in all-comers group.
	Qualitative data (patients and practitioners)	Compare experiences of EMPathicO across in-person, telephone and video consultations, and for musculoskeletal pain vs other conditions (framework analysis).
**Adoption**	Management data	Proportion and characteristics of invited practices taking part. Reasons for declining.
**Implementation**	LifeGuide usage & qualitative data	Assess patterns of usage and 'effective engagement' with EMPathicO. Explore barriers and facilitators to implementation in practice, drawing on Normalization Process Theory[86] (framework analysis).
**Maintenance**	Qualitative data (patients and practitioners)	Explore opportunities to embed EMPathicO in existing training structures. Examine longer term maintenance of practitioner behaviour change and effects on patients (reflexive thematic analysis).

For peer review only

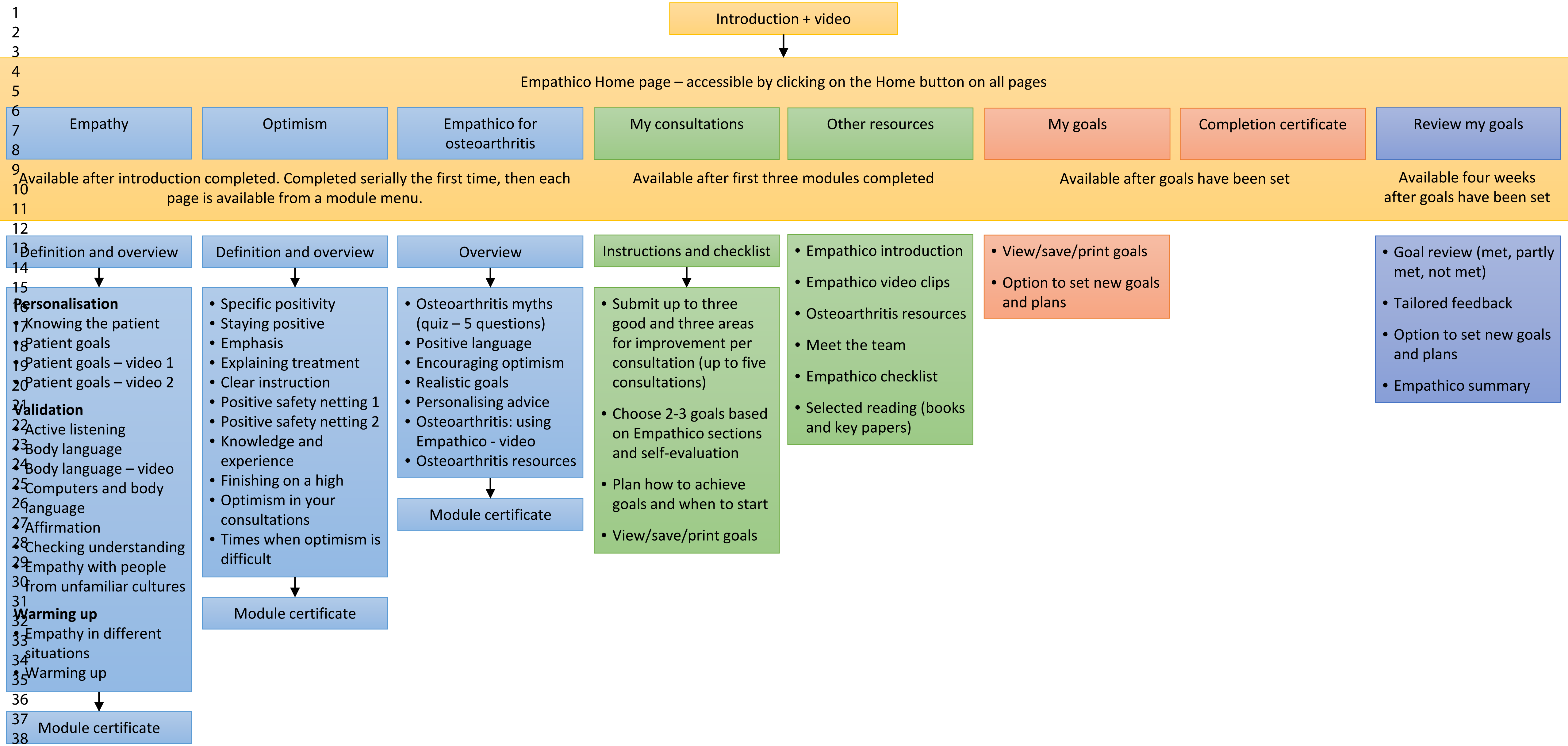
Introduction + video
Empathico Home page – accessible by clicking on the Home button on all pages
Empathy
Optimism
Empathico for osteoarthritis
My consultations
Other resources
My goals
Completion certificate
Review my goals
Available after introduction completed. Completed serially the first time, then each page is available from a module menu.
Available after first three modules completed
Available after goals have been set
Available four weeks after goals have been set
Definition and overview
Personalisation
• Knowing the patient
• Patient goals
• Patient goals – video 1
• Patient goals – video 2
Validation
• Active listening
• Body language
• Body language – video
• Computers and body language
• Affirmation
• Checking understanding
• Empathy with people from unfamiliar cultures
Warming up
• Empathy in different situations
• Warming up
Module certificate
Definition and overview
• Specific positivity
• Staying positive
• Emphasis
• Explaining treatment
• Clear instruction
• Positive safety netting 1
• Positive safety netting 2
• Knowledge and experience
• Finishing on a high
• Optimism in your consultations
• Times when optimism is difficult
Module certificate
Overview
• Osteoarthritis myths (quiz – 5 questions)
• Positive language
• Encouraging optimism
• Realistic goals
• Personalising advice
• Osteoarthritis: using Empathico - video
• Osteoarthritis resources
Module certificate
Instructions and checklist
• Submit up to three good and three areas for improvement per consultation (up to five consultations)
• Choose 2-3 goals based on Empathico sections and self-evaluation
• Plan how to achieve goals and when to start
• View/save/print goals
• Empathico introduction
• Empathico video clips
• Osteoarthritis resources
• Meet the team
• Empathico checklist
• Selected reading (books and key papers)
• View/save/print goals
• Option to set new goals and plans
• Goal review (met, partly met, not met)
• Tailored feedback
• Option to set new goals and plans
• Empathico summary

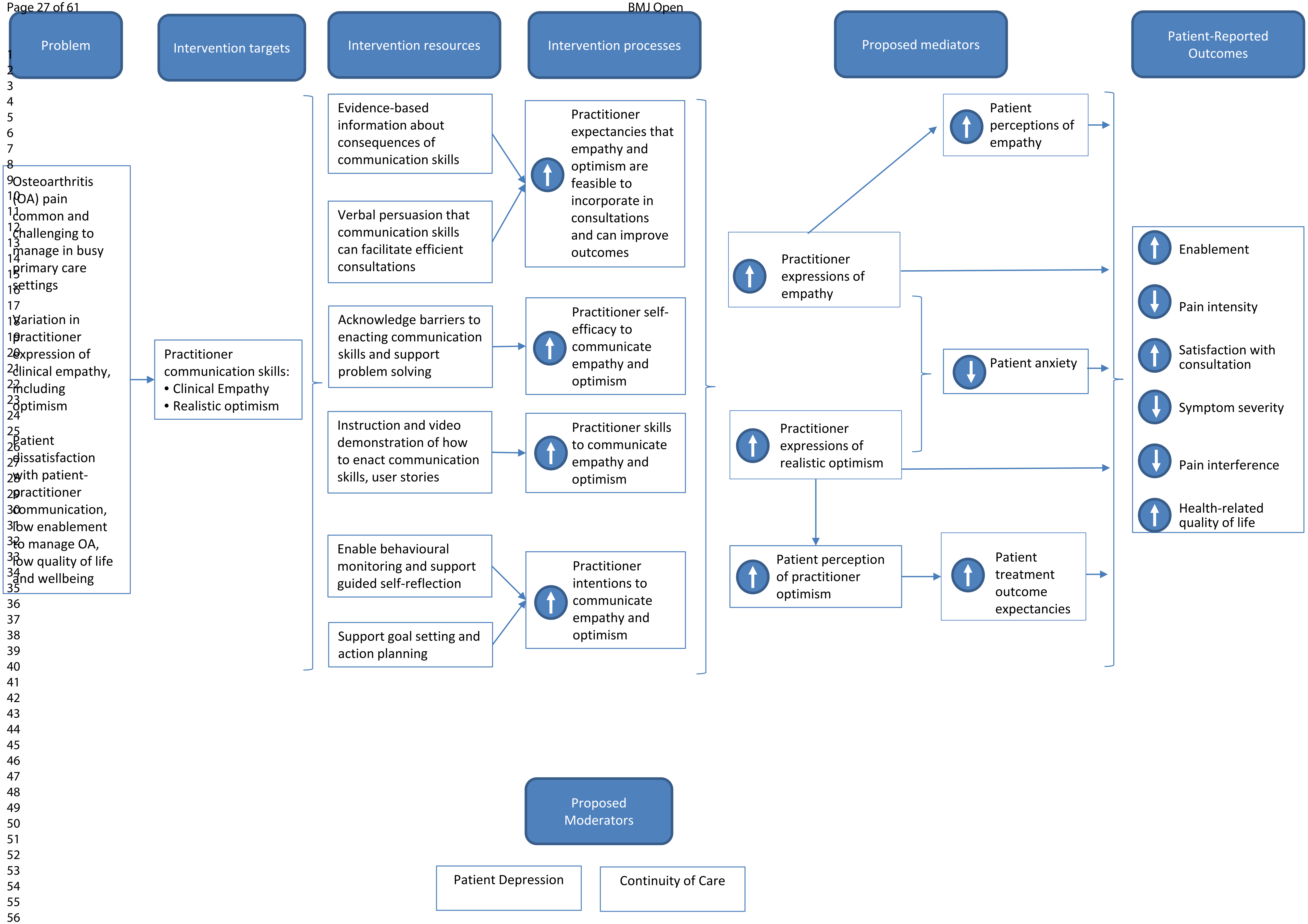
Problem
Intervention targets
Intervention resources
Intervention processes
Proposed mediators
Patient-Reported Outcomes
Osteoarthritis (OA) pain common and challenging to manage in busy primary care settings
Variation in practitioner expression of clinical empathy, including optimism
Patient dissatisfaction with patient-practitioner communication, low enablement to manage OA, low quality of life and wellbeing
Practitioner communication skills:
• Clinical Empathy
• Realistic optimism
Evidence-based information about consequences of communication skills
Verbal persuasion that communication skills can facilitate efficient consultations
Acknowledge barriers to enacting communication skills and support problem solving
Instruction and video demonstration of how to enact communication skills, user stories
Enable behavioural monitoring and support guided self-reflection
Support goal setting and action planning
Practitioner expectancies that empathy and optimism are feasible to incorporate in consultations and can improve outcomes
Practitioner self-efficacy to communicate empathy and optimism
Practitioner skills to communicate empathy and optimism
Practitioner intentions to communicate empathy and optimism
Practitioner expressions of empathy
Practitioner expressions of realistic optimism
Patient perception of practitioner optimism
Patient perceptions of empathy
Patient anxiety
Patient treatment outcome expectancies
Enablement
Pain intensity
Satisfaction with consultation
Symptom severity
Pain interference
Health-related quality of life
Proposed Moderators
Patient Depression
Continuity of Care

SPIRIT
STANDARD PROTOCOL ITEMS: RECOMMENDATIONS FOR INTERVENTIONAL TRIALS

SPIRIT 2013 Checklist: Recommended items to address in a clinical trial protocol and related documents*

Section/item	Item No	Description	
**Administrative information**			
Title	1	Descriptive title identifying the study design, population, interventions, and, if applicable, trial acronym	✓
Trial registration	2a	Trial identifier and registry name. If not yet registered, name of intended registry	✓
	2b	All items from the World Health Organization Trial Registration Data Set	✓
Protocol version	3	Date and version identifier	
Funding	4	Sources and types of financial, material, and other support	✓
Roles and responsibilities	5a	Names, affiliations, and roles of protocol contributors	✓
	5b	Name and contact information for the trial sponsor	✓
	5c	Role of study sponsor and funders, if any, in study design; collection, management, analysis, and interpretation of data; writing of the report; and the decision to submit the report for publication, including whether they will have ultimate authority over any of these activities	✓
	5d	Composition, roles, and responsibilities of the coordinating centre, steering committee, endpoint adjudication committee, data management team, and other individuals or groups overseeing the trial, if applicable (see Item 21a for data monitoring committee)	✓
**Introduction**			
Background and rationale	6a	Description of research question and justification for undertaking the trial, including summary of relevant studies (published and unpublished) examining benefits and harms for each intervention	✓
	6b	Explanation for choice of comparators	✓
Objectives	7	Specific objectives or hypotheses	✓
Trial design	8	Description of trial design including type of trial (eg, parallel group, crossover, factorial, single group), allocation ratio, and framework (eg, superiority, equivalence, noninferiority, exploratory)	✓

For peer review only

Methods: Participants, interventions, and outcomes

Study setting	9	Description of study settings (eg, community clinic, academic hospital) and list of countries where data will be collected. Reference to where list of study sites can be obtained	✓
Eligibility criteria	10	Inclusion and exclusion criteria for participants. If applicable, eligibility criteria for study centres and individuals who will perform the interventions (eg, surgeons, psychotherapists)	✓
Interventions	11a	Interventions for each group with sufficient detail to allow replication, including how and when they will be administered	✓
	11b	Criteria for discontinuing or modifying allocated interventions for a given trial participant (eg, drug dose change in response to harms, participant request, or improving/worsening disease)	✓
	11c	Strategies to improve adherence to intervention protocols, and any procedures for monitoring adherence (eg, drug tablet return, laboratory tests)	✓
	11d	Relevant concomitant care and interventions that are permitted or prohibited during the trial	✓
Outcomes	12	Primary, secondary, and other outcomes, including the specific measurement variable (eg, systolic blood pressure), analysis metric (eg, change from baseline, final value, time to event), method of aggregation (eg, median, proportion), and time point for each outcome. Explanation of the clinical relevance of chosen efficacy and harm outcomes is strongly recommended	✓
Participant timeline	13	Time schedule of enrolment, interventions (including any run-ins and washouts), assessments, and visits for participants. A schematic diagram is highly recommended (see Figure)	✓
Sample size	14	Estimated number of participants needed to achieve study objectives and how it was determined, including clinical and statistical assumptions supporting any sample size calculations	✓
Recruitment	15	Strategies for achieving adequate participant enrolment to reach target sample size	✓

Methods: Assignment of interventions (for controlled trials)

Allocation:

Sequence generation	16a	Method of generating the allocation sequence (eg, computer-generated random numbers), and list of any factors for stratification. To reduce predictability of a random sequence, details of any planned restriction (eg, blocking) should be provided in a separate document that is unavailable to those who enrol participants or assign interventions	✓

For peer review only - http://bmjopen.bmj.com/site/about/guidelines.xhtml

Allocation concealment mechanism	16b	Mechanism of implementing the allocation sequence (eg, central telephone; sequentially numbered, opaque, sealed envelopes), describing any steps to conceal the sequence until interventions are assigned	✓
Implementation	16c	Who will generate the allocation sequence, who will enrol participants, and who will assign participants to interventions	✓
Blinding (masking)	17a	Who will be blinded after assignment to interventions (eg, trial participants, care providers, outcome assessors, data analysts), and how	✓
	17b	If blinded, circumstances under which unblinding is permissible, and procedure for revealing a participant's allocated intervention during the trial	✓
**Methods: Data collection, management, and analysis**			
Data collection methods	18a	Plans for assessment and collection of outcome, baseline, and other trial data, including any related processes to promote data quality (eg, duplicate measurements, training of assessors) and a description of study instruments (eg, questionnaires, laboratory tests) along with their reliability and validity, if known. Reference to where data collection forms can be found, if not in the protocol	✓
	18b	Plans to promote participant retention and complete follow-up, including list of any outcome data to be collected for participants who discontinue or deviate from intervention protocols	✓
Data management	19	Plans for data entry, coding, security, and storage, including any related processes to promote data quality (eg, double data entry; range checks for data values). Reference to where details of data management procedures can be found, if not in the protocol	✓
Statistical methods	20a	Statistical methods for analysing primary and secondary outcomes. Reference to where other details of the statistical analysis plan can be found, if not in the protocol	✓
	20b	Methods for any additional analyses (eg, subgroup and adjusted analyses)	✓
	20c	Definition of analysis population relating to protocol non-adherence (eg, as randomised analysis), and any statistical methods to handle missing data (eg, multiple imputation)	✓
**Methods: Monitoring**			
Data monitoring	21a	Composition of data monitoring committee (DMC); summary of its role and reporting structure; statement of whether it is independent from the sponsor and competing interests; and reference to where further details about its charter can be found, if not in the protocol. Alternatively, an explanation of why a DMC is not needed	✓

For peer review only - http://bmjopen.bmj.com/site/about/guidelines.xhtml

	21b	Description of any interim analyses and stopping guidelines, including who will have access to these interim results and make the final decision to terminate the trial	✓
Harms	22	Plans for collecting, assessing, reporting, and managing solicited and spontaneously reported adverse events and other unintended effects of trial interventions or trial conduct	✓
Auditing	23	Frequency and procedures for auditing trial conduct, if any, and whether the process will be independent from investigators and the sponsor	✓
**Ethics and dissemination**			
Research ethics approval	24	Plans for seeking research ethics committee/institutional review board (REC/IRB) approval	✓
Protocol amendments	25	Plans for communicating important protocol modifications (eg, changes to eligibility criteria, outcomes, analyses) to relevant parties (eg, investigators, REC/IRBs, trial participants, trial registries, journals, regulators)	✓
Consent or assent	26a	Who will obtain informed consent or assent from potential trial participants or authorised surrogates, and how (see Item 32)	✓
	26b	Additional consent provisions for collection and use of participant data and biological specimens in ancillary studies, if applicable	N/A
Confidentiality	27	How personal information about potential and enrolled participants will be collected, shared, and maintained in order to protect confidentiality before, during, and after the trial	✓
Declaration of interests	28	Financial and other competing interests for principal investigators for the overall trial and each study site	✓
Access to data	29	Statement of who will have access to the final trial dataset, and disclosure of contractual agreements that limit such access for investigators	✓
Ancillary and post-trial care	30	Provisions, if any, for ancillary and post-trial care, and for compensation to those who suffer harm from trial participation	✓
Dissemination policy	31a	Plans for investigators and sponsor to communicate trial results to participants, healthcare professionals, the public, and other relevant groups (eg, via publication, reporting in results databases, or other data sharing arrangements), including any publication restrictions	✓
	31b	Authorship eligibility guidelines and any intended use of professional writers	✓
	31c	Plans, if any, for granting public access to the full protocol, participant-level dataset, and statistical code	✓

For peer review only

Appendices

Informed consent materials	32	Model consent form and other related documentation given to participants and authorised surrogates	✓
Biological specimens	33	Plans for collection, laboratory evaluation, and storage of biological specimens for genetic or molecular analysis in the current trial and for future use in ancillary studies, if applicable	N/A

*It is strongly recommended that this checklist be read in conjunction with the SPIRIT 2013 Explanation & Elaboration for important clarification on the items. Amendments to the protocol should be tracked and dated. The SPIRIT checklist is copyrighted by the SPIRIT Group under the Creative Commons "Attribution-NonCommercial-NoDerivs 3.0 Unported" license.

For peer review only

Online Supplementary File 2: PIS and Consent Forms

Contents

Participant Information Sheet for Patients....2
Patient Consent Form....6
Participant Information Sheet for Practitioners....7
Practitioner Consent Form....8

For peer review only

Participant Information Sheet for Patients

Version 2. Date 22.6.22.

Participant Information Sheet

Chief Investigators: Dr Felicity Bishop and Professor Hazel Everitt, University of Southampton

We invite you to take part in the TIP study

Please read on to find out why we are doing this study and what it involves. To help you decide whether to take part you may like to talk to others. If you want to talk to us, the researchers, or ask us some questions, please email <insert local researcher email address> or phone < local researcher number>. If you want to take part, you can tell us by answering the questions at the bottom of this page.

What if I need some help to take part?

We want lots of different people to take part in this study. And we know that different people will need different kinds of help.

We can help with things like understanding the documents, or if you have problems using the internet or if you would prefer paper copies of things posted to you.

If you need an interpreter, you are welcome to ask a family member or friend to help you with this study. Or you can ask us and we will do our best to get an interpreter for you.

If you would like some help to understand or take part in this study, please get in touch with us. You can contact us by phone < local researcher number >or email <<insert local researcher email address>>

A quick summary of the study

- This study will help us understand patients' experiences of appointments with GPs, Nurses, or Physiotherapists. We'll call these people "Primary Care Practitioners".
- We want to know what you think about how your Primary Care Practitioner talks to you during consultations.
- The study is being run by The Universities of Southampton, Bristol, Warwick, Oxford, and Keele University. It is funded by the National School for Primary Care Research (SPCR).
- The South Central-Hampshire B Research Ethics Committee has given a favourable opinion of the study. This means that a group of independent people have looked at our research and feel that it is ethically acceptable.

Why have I been asked to participate?

For peer review only - http://bmjopen.bmj.com/site/about/guidelines.xhtml

Because you are an adult and have an appointment with a Primary Care Practitioner who is already taking part in the TIP study

What will happen if I take part?

We will ask you to read some documents (like this one) and fill in some questionnaires.

Before your appointment
Read this information.<br>Answer a few questions to check you can take part in the study.<br>Answer a few questions to tell us if you want to take part in the study.

↓

During your appointment
Your consultation at your GP surgery will happen as normal.<br>**Please do not discuss this study with your Primary Care Practitioner.**<br>It is important that your Primary Care Practitioner does not know whether you are taking part.

↓

Within 1 week of your appointment
You will be sent a link to fill in some questionnaires. These questionnaires ask about your appointment, your health, and your quality of life. They take about 15 minutes to do.

↓

1 month, 3 months, and 6 months after your first appointment
You will be sent a link to fill in some questionnaires. These questionnaires ask about your health and quality of life. They take about 15 minutes to do.

We might also invite you to take part in two meetings (interviews) with a researcher. If you are asked to do an interview, this would be in the first week after your appointment and again in 6 months' time. In the interview, the researcher will ask about your experiences of primary care appointments and your experiences of doing this study..

What are the possible pros and cons of taking part?

Taking part will help us understand the best ways for primary care practitioners to talk to patients during consultations. We do not think that taking part in this study poses any risks for you To thank you for taking part we will give you two £10 vouchers. We will send the first voucher after you complete the 1 month questionnaire. We will send the second voucher after you complete the 6 month questionnaire. If you take part in an interview as well as doing the questionnaires, then we will send you an extra £10 voucher for each interview.

For peer review only - http://bmjopen.bmj.com/site/about/guidelines.xhtml

Do I have to take part?

No, it is up to you to decide .

If you decide to take part now, you can still change your mind later. You can pull out from the study at any time by contacting the researcher by email or phone. You won't have to give a reason. Your routine health care won't be affected at all. If you pull out of the study, we will keep the information that you've already given us.

What information will be collected?

You will probably fill in our questionnaires on the internet. Although, if you would rather have a paper questionnaire please ask us and we can give you one.

Our questionnaires are on a secure service called Qualtrics. Qualtrics meets the highest standards for privacy and data security. We will download all the completed questionnaires. We will store this data on a University of Southampton computer server behind the University of Southampton firewall. At the end of the study, we will destroy our records of your personal contact details.

Your name will not appear on any questionnaires you fill in. Your questionnaire answers will be combined with other patients' answers and put in a secure data archive. Only suitably qualified researchers are allowed to ask for access this archive.

One of our questions asks if it's OK to use your questionnaire answers to help other ethically approved research and education activities in the future. If you say "no" you can still take part in the study. Personal data will be collected and stored on a secure server at University of Southampton in compliance with the requirements of the General Data Protection Regulations and the Data Protection Act 2018. We will securely store your name, contact details, and any other personal data you have given us in a separate list, so we know who has taken part. We will only use your contact details to contact you about this study. You do not need to but if you would like to read the full Data Protection Privacy Notice, click here.

Will my participation be confidential?

Your participation and the information we collect about you during the course of the research will be kept strictly confidential.

Nothing you say on the questionnaires or in the interviews will be shared with your Primary Care Practitioner or anyone else in the medical practice.

But, if you say something in an interview which makes the interviewer worried that you might be being abused or neglected then they will raise this with the appropriate people.

The research team may have to give certain other people access to your data. The only other people who might be given access to your data are responsible members of the University of Southampton and regulatory authorities (for example, the Health Research Authority). They need access to make sure the research is being done correctly and in line with regulations. All of these people must keep your information, strictly confidential.

For peer review only - http://bmjopen.bmj.com/site/about/guidelines.xhtml

What will happen to the results of the research?

We hope to publish our results in scientific journals, blogs and conferences. We plan to share our findings with a wide audience including health care professionals, scientists, patients and members of the public. If you would like, we can also send you a summary what we found out. You can ask for this summary when you fill in the questionnaires.

What happens if there is a problem?

If you have a concern about any aspect of this study, you should speak to Nadia Cross who will do her best to answer your questions.

If you remain unhappy or have a complaint about any aspect of this study, please contact the University of Southampton Research Integrity and Governance Manager (023 8059 5058, rgoinfo@soton.ac.uk).

You may also contact your local Patient Advice and Liaison Service (PALS). PALS has been introduced to ensure that the NHS listens to patients, their relatives, carers, and friends, and answers their questions and resolves their concerns as quickly as possible. Your local PALS service can be found at <<INSERT LOCAL DETAILS>>

Where can I get more information?

PLEASE DO NOT DISCUSS YOUR PARTICIPATION IN THE STUDY WITH YOUR GP, NURSE, PHYSIOTHERAPIST, OR ANY OTHER PRIMARY CARE PRACTITIONER.

If you have any questions about the study, you can contact the researcher, <<INSERT NAME>>

Email: <<INSERT>>; or Telephone: TBC>>

You can also contact the study manager, Nadia Cross at tip@soton.ac.uk

Thank you for reading this information and considering taking part in our study.

For peer review only - http://bmjopen.bmj.com/site/about/guidelines.xhtml

Patient Consent Form

Version 2. Date 22.6.22.

Patient Consent Form

Chief Investigators: Dr Felicity Bishop and Professor Hazel Everitt, University of Southampton

**Please indicate if you agree with the statement.**	**Yes/No**
I have read and understood the information sheet *(insert date /version no. of participant information sheet)* and have had the opportunity to ask questions about the study.	Yes/No
I agree to take part in this research project and agree for my data to be used for the purpose of this study.	Yes/No
I understand my participation is voluntary and I may withdraw at any time without giving a reason and without my routine health care being affected.	Yes/No
I understand that personal details I provide will be held securely at The University of Southampton in line with General Data Protection Regulation and Data Protection Act 2018.	Yes/No
I agree to take part in this study	Yes/No
Optional: You do not have to agree to this to take part in this research<br><br>I agree that the information collected about me may be used to support other ethically approved research and education activities in the future, and may be stored in a secure data archive and shared anonymously with other suitably-qualified researchers.	Yes/No

For peer review only

Participant Information Sheet for Practitioners

Version 1. Date 23.3.22.

Practitioner Information Sheet (main study)

Chief Investigators: Dr Felicity Bishop and Professor Hazel Everitt, University of Southampton

We invite you to take part in a research study

It is up to you to decide if you want to take part or not. This leaflet tells you why the study is being done and what it will involve. Please discuss this information with others if you wish. Please contact the research team if anything is unclear or you would like to ask any questions.

A quick summary of the study

- In this cluster randomised trial, your practice will be randomised into one of two groups: intervention arm or control arm.
- Practitioners working in intervention practices will complete communication skills e-learning training and implement the skills in subsequent consultations. Practitioners working in control practices will continue consulting as usual.
- Patients will be recruited at the intervention and control practices, and complete pre-consultation and post-consultation questionnaires.
- Practitioners in both arms will be asked to complete online questionnaires (about communication within consultations) at 3 time points: baseline, 8 weeks, and 34 weeks post-randomisation. Practitioners in the control group will have access to the communication skills e-learning training at the end of the study.
- The study is being run by the Universities of Southampton, Bristol, Keele, Oxford and Warwick, and is funded by the National School for Primary Care Research (SPCR).

What is the research about?

We have developed communication skills e-learning training for GPs, physiotherapists, and nurses to help enhance consultations with osteoarthritis patients. It is also likely that this training will be relevant to other conditions. The TIP (Talking in Primary Care) study aims to test the effectiveness

For peer review only - http://bmjopen.bmj.com/site/about/guidelines.xhtml

and cost-effectiveness of communication skills e-learning training for primary care practitioners on patients' musculoskeletal pain and enablement.

Why have I been asked to participate?

You have been asked to take part because you are a GP, physiotherapist or nurse working in primary care, and have experience of treating patients with osteoarthritis. We hope to recruit a range of practitioners with different levels of experience and background.

Do I have to take part?

No, it is entirely up to you to decide whether or not to take part. If you decide that you would like to take part, we will ask you to complete an online consent form.

What will happen to me if I take part?

If you are interested in taking part:

- You will be provided with a link to a study website, provide online consent and complete an online questionnaire (approx. 10 minutes).
- Your practice will be randomised to one of two groups: an intervention arm and a control arm.
- In weeks 1-2, if you are in the intervention arm you will be asked to complete the training. This will take approximately 1-2 hours and can be done in short chunks. If you are in the control arm, you should continue to treat patients as usual and not undertake any training in communication skills.
- In weeks 3-8, we will be recruiting patients from your practice to take part in this study. You may be asked to help with this.
- You will be asked to complete a short online questionnaire about communication within consultations at 3 time points: baseline, 8 weeks, and 34 weeks post-randomisation.
- You will also be offered the opportunity to take part in a research interview to share your experiences of communication within consultations and the TIP study.
- If you are in the control arm, you will be offered access to the e-learning training at the end of the study.

What are the possible pros and cons of taking part?

Participating in the TIP study will give you the opportunity to learn and implement evidence-based communication skills within your consultations. This could improve patient outcomes and patient satisfaction with care and make best use of primary care appointments. There are no expected risks or disadvantages associated with taking part in this study.

For peer review only - http://bmjopen.bmj.com/site/about/guidelines.xhtml

GP practices will be paid service support costs/ excess treatment costs via their CRN for taking part in the TIP study. We will also provide research costs to reimburse practitioners for their time spent taking part in the study.

What happens to the data collected?

- Electronic questionnaires will be collected using a secure online data collection service which meets the highest industry standards for privacy and data security (Qualtrics).
- Data on patterns and amount of usage of the e-learning training will be collected by the LifeGuide platform on which the e-learning training is hosted.
- All data from Qualtrics and LifeGuide will be downloaded to University of Southampton servers, password-protected and stored securely behind the University of Southampton firewall.
- At the end of the study anonymous questionnaire data will be deposited in a secure data archive which will be made available on request to suitably qualified researchers for further data analysis on this topic.

We will securely store your name and contact details separately from your questionnaire data and will only use these details to contact you about this study. We will permanently delete this at the end of the project.

Will my participation be confidential?

Your participation and the information we collect about you during the course of the research will be kept strictly confidential.

Only members of the research team and responsible members of the University of Southampton may be given access to data about you for monitoring purposes and/or to carry out an audit of the study to ensure that the research is complying with applicable regulations. Individuals from regulatory authorities (people who check that we are carrying out the study correctly) may require access to your data. All of these people have a duty to keep your information, as a research participant, strictly confidential.

What happens if I change my mind?

You have the right to change your mind and withdraw at any time without giving a reason. If you wish to withdraw from the study, please contact Nadia Cross, Trial Manager (details below).

What will happen to the results of the research?

We hope to publish our results in scientific journals and other formats such as blogs and conferences. We plan to share our findings with a wide audience including health care professionals, scientists, patients, and members of the public. If you would like, we will also send you a summary of our findings.

Who is conducting the study?

For peer review only - http://bmjopen.bmj.com/site/about/guidelines.xhtml

Our research team includes GPs, health psychologists, academic researchers and patient representatives from the Universities of Southampton, Bristol, Keele, Oxford and Warwick. The research is funded by National School for Primary Care Research (SPCR) and has been approved by the Health Research Authority and the National Research Ethics Committee (reference number: <<xxxxxxxx>>). The research is being sponsored by University of Southampton.

What happens if there is a problem?

If you have a concern about any aspect of this study, you should speak to the researchers (contact details above) who will do their best to answer your questions.

If you remain unhappy or have a complaint about any aspect of this study, please contact the University of Southampton Research Integrity and Governance Manager (023 8059 5058, rgoinfo@soton.ac.uk).

Where can I get more information?

If you have any questions, please do not hesitate to get in touch with Nadia Cross, Trial Manager using the contact details below:

**Name**	**Nadia Cross**	
**Role:**	Trial Manager	
**Address:**	University of Southampton<br>Aldermoor Health Centre<br>Southampton, SO16 5ST	
**Contact:**	*[insert study team contact details@soton.ac.uk]*	

Thank you for taking the time to read the information sheet and considering taking part in the research

** Click out page in Qualtrics*

Data Protection Privacy Notice

The University of Southampton conducts research to the highest standards of research integrity. As a publicly-funded organisation, the University has to ensure that it is in the public interest when we use personally-identifiable information about people who have agreed to take part in research. This means that when you agree to take part in a research study, we will use information about you in the ways needed, and for the purposes specified, to conduct and complete the research project. Under data protection law, 'Personal data' means any information that relates to and is capable of identifying a living individual. The University's data protection policy governing the use of personal data by the University can be found on its website (https://www.southampton.ac.uk/legalservices/what-we-do/data-protection-and-foi.page).

For peer review only - http://bmjopen.bmj.com/site/about/guidelines.xhtml

This Participant Information Sheet tells you what data will be collected for this project and whether this includes any personal data. Please ask the research team if you have any questions or are unclear what data is being collected about you.

Our privacy notice for research participants provides more information on how the University of Southampton collects and uses your personal data when you take part in one of our research projects and can be found at http://www.southampton.ac.uk/assets/sharepoint/intranet/ls/Public/Research%20and%20Integrity%20Privacy%20Notice/Privacy%20Notice%20for%20Research%20Participants.pdf

Any personal data we collect in this study will be used only for the purposes of carrying out our research and will be handled according to the University's policies in line with data protection law. If any personal data is used from which you can be identified directly, it will not be disclosed to anyone else without your consent unless the University of Southampton is required by law to disclose it.

Data protection law requires us to have a valid legal reason ('lawful basis') to process and use your Personal data. The lawful basis for processing personal information in this research study is for the performance of a task carried out in the public interest. Personal data collected for research will not be used for any other purpose.

For the purposes of data protection law, the University of Southampton is the 'Data Controller' for this study, which means that we are responsible for looking after your information and using it properly. The University of Southampton will keep identifiable information about you for 10 years after the study has finished after which time any link between you and your information will be removed.

To safeguard your rights, we will use the minimum personal data necessary to achieve our research study objectives. Your data protection rights – such as to access, change, or transfer such information - may be limited, however, in order for the research output to be reliable and accurate. The University will not do anything with your personal data that you would not reasonably expect.

If you have any questions about how your personal data is used, or wish to exercise any of your rights, please consult the University's data protection webpage (https://www.southampton.ac.uk/legalservices/what-we-do/data-protection-and-foi.page) where you can make a request using our online form. If you need further assistance, please contact the University's Data Protection Officer (data.protection@soton.ac.uk).

For peer review only - http://bmjopen.bmj.com/site/about/guidelines.xhtml

Practitioner Consent Form

Version 2. Date 22.6.22.

Practitioner consent form (main study)

Chief Investigators: Dr Felicity Bishop and Professor Hazel Everitt, University of Southampton

ERGO number: 70489

For peer review only

Please indicate if you agree with the statements:

1. I have read and understood the practitioner information sheet (<<insert version and date>> and have had the opportunity to ask questions about the study.	Yes/No
2. I agree to take part in this research project and agree for my data to be used for the purpose of this study.	Yes/No
3. I understand my participation is voluntary and I may withdraw at any time for any reason.	Yes/No
4. I understand that personal details I provide will be held securely at The University of Southampton in line with General Data Protection Regulation and Data Protection Act 2018.	Yes/No
5. I agree to take part in the TIP study.	Yes/No
Optional: You do not have to agree to this item to take part in this research 6. I agree that my questionnaire data may be used to support other ethically approved research and education activities in the future and may be stored in a secure data archive and shared anonymously with other suitably qualified researchers.	Yes/No

Name of participant ……………………………………………………………………………

Date……………………………………

Online Supplementary File 3: Measures and Timings

Contents

Table 1. Patient-Reported Characteristics, Primary and Secondary Outcomes and Process Variables............2
Table 2. Practitioner-Reported Characteristics, Outcomes and Process Variables 3
References 4

For peer review only

Table 1. Patient-Reported Characteristics, Primary and Secondary Outcomes and Process Variables

Variable	Measure	Items	Measurement Timings				
			<-7d	<7 d	+1 m	+3 m	+6 m
Primary Outcomes							
Pain intensity (pain sample)	Pain intensity subscale from the BPI[1]	4	x	x	x	x	x
Patient enablement	Modified PEI[2]	6		x	x	x	x
Secondary Outcomes							
Patient global impression of symptom severity	Single item[3]	1	x	x	x	x	x
Patient global impression of symptom change	Single item[3]	1		x	x	x	x
Pain interference	Pain interference subscale from the BPI[1]	7			x		X
Patient satisfaction	MISS for UK general practice[4]	21		x			
Adverse events	Bespoke self-report item	1			x	x	x
Health Economics							
Health-related quality of life	EQ-5D-5L and EQ-VAS[5]	6	x		x		x
Capability wellbeing	ICECAP-A[6 7]	5	x		x		x
Healthcare utilization	ModRUM core module[8]	12		x		x	x
Prescribed medications	ModRUM depth questions[8]	1				x	x
Personal expenses	Bespoke self-report item	3				x	x
Productivity	WPAI:GH	6				x	x
Process Measures							
Perceptions of practitioner empathy	CARE[9]	10		X			
Perceptions of practitioner optimism	Bespoke item	1		X			
Treatment expectations	Treatment expectation questionnaire TEX-Q[10]	15		X			
Anxiety	HADS[11 12]	7		X			
Continuity of care	Patient-Doctor Depth of Relationship Scale[13]	9		X			
Depression	HADS[11 12]	7		X			
Sociodemographic Characteristics							
Age, gender, ethnicity		3	x				
Index of Multiple Deprivation	Postcode	1	x				
Health Characteristics							
Reasons for consulting		1		x			
Comorbidities		1		x			
Index consultation modality		1		x			

Table 2. Practitioner-Reported Characteristics, Outcomes and Process Variables

Practitioners	Variable	Measure	Items	Measurement Timings			
				Baseline	+2wk	+8wk	+34wk
All	Characteristics (age, gender, ethnicity, years qualified, profession)	Bespoke	5	x			
All	Practitioner self-efficacy for conveying clinical empathy	Bespoke, from feasibility study	7	X		X	x
All	Practitioner self-efficacy for conveying realistic optimism	Bespoke, from feasibility study	5	x		X	x
Intervention arm only	Practitioner outcome expectancy for implementing goals set during EMPathicO training	Bespoke, from feasibility study	16	X		X	x
Intervention arm only	Practitioner intentions to implement goals set during EMPathicO training	Bespoke, from feasibility study	3	X		X	x
Intervention arm only	Practitioner intervention usage	LifeGuide data	N/A			X	X
All	Practitioner-reported other training	Bespoke	1			x	x

For peer review only

References

1. Keller S, Bann CM, Dodd SL, et al. Validity of the Brief Pain Inventory for use in documenting the outcomes of patients with noncancer pain. *Clin J Pain* 2004;20(5):309-18.
2. Howie JG, Heaney DJ, Maxwell M, et al. A comparison of a Patient Enablement Instrument (PEI) against two established satisfaction scales as an outcome measure of primary care consultations. *Fam Pract* 1998;15(2):165-71.
3. Fischer D, Stewart AL, Bloch DA, et al. Capturing the Patient's View of Change as a Clinical Outcome Measure. *JAMA* 1999;282(12):1157-62. doi: 10.1001/jama.282.12.1157
4. Meakin R, Weinman J. The 'Medical Interview Satisfaction Scale' (MISS-21) adapted for British general practice. *Fam Pract* 2002;19(3):257-63. doi: 10.1093/fampra/19.3.257
5. Herdman M, Gudex C, Lloyd A, et al. Development and preliminary testing of the new five-level version of EQ-5D (EQ-5D-5L). *Qual Life Res* 2011;20(10):1727-36. doi: 10.1007/s11136-011-9903-x [published Online First: 2011/04/12]
6. Al-Janabi H, Flynn TN, Coast J. Development of a self-report measure of capability wellbeing for adults: the ICECAP-A. *Qual Life Res* 2012;21(1):167-76. doi: 10.1007/s11136-011-9927-2 [published Online First: 2011/05/21]
7. Keeley T, Coast J, Nicholls E, et al. An analysis of the complementarity of ICECAP-A and EQ-5D-3 L in an adult population of patients with knee pain. *Health and quality of life outcomes* 2016;14:36. doi: 10.1186/s12955-016-0430-x [published Online First: 2016/03/05]
8. Garfield K, Husbands S, Thorn JC, et al. Development of a brief, generic, modular resource-use measure (ModRUM): cognitive interviews with patients. *BMC Health Serv Res* 2021;21(1):371. doi: 10.1186/s12913-021-06364-w
9. Mercer SW, Maxwell M, Heaney D, et al. The development and preliminary validation of the Consultation and Relational Empathy (CARE) measure: an empathy-based consultation process measure. *Fam Pract* 2004;21 699-705.
10. Alberts J, Löwe B, Glahn MA, et al. Development of the generic, multidimensional Treatment Expectation Questionnaire (TEX-Q) through systematic literature review, expert surveys and qualitative interviews. *BMJ Open* 2020;10(8):e036169. doi: 10.1136/bmjopen-2019-036169
11. Bjelland I, Dahl AA, Haug TT, et al. The validity of the Hospital Anxiety and Depression Scale: An updated literature review. *J Psychosom Res* 2002;52(2):69-77.
12. Zigmond AS, Snaith RP. The Hospital Anxiety and Depression Scale. *Acta Psychiatr Scand* 1983;67(6):361-70.
13. Ridd MJ, Lewis G, Peters TJ, et al. Patient-Doctor Depth-of-Relationship Scale: Development and Validation. *The Annals of Family Medicine* 2011;9(6):538. doi: 10.1370/afm.1322

For peer review only

Talking in Primary Care: A cluster-randomized controlled trial in primary care to test the effectiveness and cost-effectiveness of communication skills e-learning for practitioners on patients' musculoskeletal pain and enablement

Trial Steering Committee Charter

Version 1 22 April 2022

Authorised by:

Name:	Professor Joanne Reeve	Role:	Chairperson
Signature:	Joanne Reeve	Date:	22 April 2022

Prepared by

Name:	Nadia Cross	Role:	Trial Manager
Signature:	[illegible]	Date:	22 April 2022

CONTENT	DETAILS OF TSC
**1. Introduction**	
Name (& Sponsor's ID) of trial	Talking in Primary Care: A cluster-randomized controlled trial in primary care to test the effectiveness and cost-effectiveness of communication skills e-learning for practitioners on patients' musculoskeletal pain and enablement.<br>**UoS ERGO:** 70489<br>**IRAS:** 312208
Objectives of trial, including interventions being investigated	The primary aim is to determine the clinical and cost-effectiveness of EMPathicO training in Clinical Empathy and conveying realistic Positive Messages for practitioners in patients presenting with MSK pain.<br><br>The secondary aim is to maximize EMPathicO's potential for wide-spread adoption, implementation, and maintenance of effects. We will do this by assessing effects of EMPathicO training on patients presenting with any symptoms other than MSK pain since the impact of EMPathicO will potentially be in all consultations not just MSK consultations; testing how and in what circumstances EMPathicO changes practitioner communication behaviours and patient outcomes for in-person, telephone, and video consultations; and analysing a diverse range of patients' and practitioners' experiences of adoption and longer-term implementation.
Outline of scope of Charter	The purpose of this document is to describe the membership, terms of reference, roles, responsibilities, authority, decision-making and relationships of the Trial Steering Committee (TSC) and the Data Monitoring Committee (DMC) for this trial, including the timing of meetings, methods of providing information to and from the TSC, frequency and format of meetings and relationships with other trial committees.
Facilitation	A member of the TIP team will be nominated as a Facilitator for the trial. The Facilitator will be responsible for the organisation of meetings and should be copied into all communications with and between the TSC.
**2. Roles and responsibilities**	
A broad statement of the aims of the TSC	TSC - To act as the oversight body for the TIP study on behalf of the Sponsor/Funder.<br><br>DMC - To monitor and review on a 6 monthly basis the main outcomes measures overall conduct in order to safeguard the interests of patients
Terms of reference	The role of the TSC is to provide oversight for the TIP study. It should also provide advice through its independent Chairperson to the Trial Management Group (TMG) and the funder (NIHR-SPCR) on all aspects of the trial.<br><br>The TSC will also assume responsibilities of the Data Monitoring Committee (DMC) and review information on the progress and accruing data of this trial and provide advice on the conduct of the trial.

For peer review only - http://bmjopen.bmj.com/site/about/guidelines.xhtml

CONTENT	DETAILS OF TSC
Specific roles of TSC	• provide expert oversight of the trial<br>• maintain confidentiality of all trial information that is not already in the public domain<br>• make decisions as to the future continuation (or otherwise) of the trial/s<br>• monitor recruitment rates and encourage the TMG to develop strategies to deal with any recruitment problems<br>• comment on the protocol<br>• assess the impact and relevance of any accumulating external evidence<br>• review completion of CRFs and comment on strategies from TMG to encourage satisfactory completion in the future<br>• monitor follow-up rates and review strategies from TMG to deal with problems<br>• censure sites that are deviating from the protocol<br>• comment on any amendments to the protocol, where appropriate<br>• approve any proposals by the TMG concerning any change to the design of the trial, including additional sub-studies<br>• oversee the timely reporting of trial results<br>• comment on the statistical analysis plan<br>• comment on the publication policy<br>• comment on the main trial manuscript<br>• comment on any abstracts and presentations of any results during the running of the trial
Specific roles of DMC delegated to the TSC	Interim review of the trial's progress including updated figures on recruitment, data quality, adherence to protocol treatment and follow-up, and main outcomes and safety data. Specifically, these roles include to:<br>• monitor evidence for treatment harm (e.g. SAEs and deaths)<br>• assess the impact and relevance of external evidence<br>• decide whether to recommend that the trial continues to recruit participants or whether recruitment should be terminated either for everyone or for some treatment groups and/or some participant subgroups<br>• decide whether trial follow-up should be stopped earlier<br>• assess data quality, including completeness (and by so doing encourage collection of high quality data)<br>• maintain confidentiality of all trial information that is not in the public domain<br>• monitor recruitment figures and losses to follow-up<br>• monitor compliance with the protocol by participants and investigators<br>• monitor planned sample size assumptions.<br>• suggest additional data analyses if necessary<br>• advise on protocol modifications proposed by investigators or sponsors (e.g. to inclusion criteria, trial endpoints, or sample size)<br>• monitor continuing appropriateness of patient information

CONTENT	DETAILS OF TSC
**3. Before or early in the trial**	
Whether the TSC will have input into the protocol	All potential TSC members should have sight of the protocol as early as possible. Before recruitment begins the trial will have undergone review by the Sponsor/Funder (e.g. peer review for public sector trials), scrutiny by other trial committees and a research ethics committee. Therefore, if a potential TSC member has major reservations about the trial (e.g. the protocol, the logistics, ethical concerns) they should report these to TMG. TSC members should be constructively critical of the ongoing trial, but also supportive of aims and methods of the trial.
Whether members of the TSC will have a contract	TSC members will not be asked to formally sign a contract but should formally register their agreement to join the group by confirming (1) that they agree to be a member and (2) that they agree with the contents of this Charter. Any potential competing interests should be declared at the same time. Members should complete and return the form in Annexes 1 or 2. Any observers (attendees who are not members) will sign a confidentiality agreement on the first occasion they attend a meeting (Annexe 3).
**4. Composition**	
Membership and size of the TSC	The majority of members of the TSC, including the Chair, should be independent [1]of the trial (see section 5). Non-independent members will also be part of the TSC.<br>The members of the TSC for this trial are:<br>Professor Joanne Reeve (chair) – Independent member<br>Dr Philip Pallmann – Independent member<br>Dr Ines Rombach – Independent member<br>Mr Ian Dickerson – PPI contributor<br>Dr Felicity Bishop – Co-Chief Investigator<br>Professor Hazel Everitt – Co-Chief Investigator
Tenure	Until 30/06/2024.
The Chair, how they are chosen and the Chair's role.	The Chair should have previous experience of serving on trial committees and experience of Chairing meetings, and should be able to facilitate and summarise discussions; knowledge of the disease area would be beneficial.
The responsibilities of the Facilitator	The Facilitator will be a member of staff in Southampton Primary Care Research Centre, University of Southampton. The Facilitator will be responsible for arranging meetings of the TSC, coordinating reports, producing and circulating minutes and action points. The Facilitator will be the central point for all TSC communications between the TSC and other bodies, will be copied into all correspondence between TSC members and will be kept aware of trial issues as they arise.
The responsibilities of the TIP team	The TIP team will produce a short report on the trial before each meeting of the TSC.

[1] Independence is defined in Table 1 of Annexe 1

For peer review only - http://bmjopen.bmj.com/site/about/guidelines.xhtml

CONTENT	DETAILS OF TSC
The responsibilities of the CI and other members of the TMG	The CI (and, if appropriate, other TMG members) is an important member of the TSC and no major decisions should be made without their involvement.
The responsibilities of the observers	Additional observers may be in attendance through (parts of) the TSC meetings in order to provide input on behalf of the TIP team, the trial's Sponsor/Funder or to provide specific relevant expertise.
**5. Relationships**	
Advisory and executive bodies	The TSC is the oversight body and is delegated the roles in Section 2 by the Sponsor. All substantial issues regarding the trial must go to the TSC for consideration.
Payments to TSC members	Members will be reimbursed for reasonable travel costs and other expenses incurred. No other payments or rewards would be given professional members. Honoraria will be paid to lay members according to the INVOLVE guidelines.
The need for TSC members to disclose information about any real or potential competing interests	Any competing interests, both real or potential, should be disclosed. These are not restricted to financial matters – involvement in other trials or intellectual investment could be relevant. Although members may well be able to act objectively despite such connections, complete disclosure enhances credibility. (See Annex 1)<br>TSC members should not use any trial data to inform trading in pharmaceutical shares, and careful consideration should be given to trading in stock of companies with competing products. Changes in declarations of real or potential competing interests should be minuted at the start of each meeting.
**6. Organisation of meetings**	
Expected frequency of TSC meetings	The TSC will meet in person at least yearly if possible. At the request of the TSC, interim meetings, in person or by teleconference, will be organised. Major trial issues may need to be dealt with between meetings, by phone or by email. TSC members should be prepared for such instances.
Attendance of TSC members at meetings	Effort will be made to ensure that all members can attend. The Facilitator will work for a date that enables this. The CI must try to attend all meetings, especially if major actions are expected. Members who cannot attend in person should be encouraged to participate by teleconference. If, at short notice, any TSC members cannot attend then the TSC may still meet if at least two independent members, including the Chair (unless otherwise agreed), will be present, plus also a member of the trial team. If the TSC is considering a major action after such a meeting the TSC Chair should communicate with the absent members, including the CI, as soon after the meeting as possible to check they agree. If they do not, a further teleconference should be arranged with the full TSC.
How TSC meetings will be organised, especially regarding open and closed sessions,	Presence will be usually limited to the TSC members, observers from the Sponsor/Funder, TIP team and the Facilitator. Other attendees may be invited for all or part of the meeting by the TSC including

For peer review only - http://bmjopen.bmj.com/site/about/guidelines.xhtml

CONTENT	DETAILS OF TSC
including who will be present in each session	the trial statistician and trial manager. The observers are not members of the TSC but may be invited to provide expert input or to represent the funding bodies involved; other observers will be at the discretion of the TSC and the Facilitator but may include members of the TMG other than the CI.
Can TSC members who cannot attend the meeting input	If the report is circulated before the meeting, TSC members who will not be able to attend the meeting may pass comments to the TSC Chair, Facilitator or TIP team for consideration during the discussions.
What happens to independent members who do not attend meetings	If an independent member does not attend a meeting or provide comments when requested between meetings, it should be ensured that the independent member is available for the next meeting. If an independent member does not attend the next meeting or provide comments when next requested, they should be asked if they wish to remain part of the TSC.
**7. Trial documentation and procedures to ensure confidentiality and proper communication**	
Intended content of material to be considered during meetings	A short report will be prepared by the TIP team. This will report on accrual and any matters affecting the trial. Additionally, the material may include requests *from* the TMG or draft publications. Where relevant, accrual, compliance with follow-up and adherence to treatment may be presented by centre.
Whether reports to the TSC be available before the meeting or only at/during the meeting	It is usually helpful for the TSC to receive the report at least 1 week and preferably at least 2 weeks before any meetings. Different procedures may apply to teleconference meetings.
Responsibility for identifying and circulating external evidence (e.g. from other trials/ systematic reviews)	Identification and circulation of external evidence (e.g. from other trials/ systematic reviews) is not the responsibility of the TSC members; it is a responsibility of the TMG. However, the TSC should continue to be made aware of other data that may impact on a trial.
What will happen to the papers after the meeting	TSC members would be expected to delete, destroy or store securely copies of the reports to and from the TSC, agenda and minutes, as well as copies of communications between meetings. All documentation should be considered confidential. The Facilitator will keep a central record of all minutes, reports and correspondence by the TSC.
**8. Decision making**	
What decisions will be open to the TSC	Possible decisions include:-<br>• No action needed, trial continues as planned<br>• Early stopping due, for example, to clear benefit or harm of a treatment, futility or external evidence.<br>• Stopping recruitment within a subgroup.<br>• Modifying target recruitment, or pre-analysis follow-up, based on any change to the assumptions underlying the original trial sample size calculation (but not on any emerging differences)<br>• Sanctioning and/or proposing protocol changes
How decisions or	Every effort should be made to achieve consensus. The role of the

For peer review only - http://bmjopen.bmj.com/site/about/guidelines.xhtml

CONTENT	DETAILS OF TSC
recommendations will be reached within the TSC	Chair is to summarise discussions and encourage consensus; therefore, it is usually best for the Chair to give their own opinion last.<br>It is important that the implications (e.g. ethical, statistical, practical, financial) for the trial be considered before any decision is made.
When the TSC is quorate for decision-making	At least two independent members of the TSC should be present including the Chair, plus the CI if a major action is to be considered.
**9. Reporting**	
To whom will the TSC report their recommendations/decisions, and in what form	The TSC will report their decisions (via the Facilitator) to the TMG who will be responsible for implementing any actions resulting. The TSC may also provide feedback to the Sponsor/Funder. Copies of communications will pass through the Facilitator.
Whether minutes of the meeting be made and, if so, by whom and where they will be kept	Notes of key points and actions will be made by the Facilitator. This will include details of whether potential competing interests have changed for any attendees since the previous meeting. The draft minutes will be initially circulated for comment to those TSC members who were present at the meeting. The TSC Chair will sign off the final version of minutes or notes.
**10. After the trial**	
Publication of results	The TSC will oversee the timely analysis, writing up and publication of the main trial results. The independent members of the TSC will have the opportunity to read and comment on the proposed main publications of trial data prior to submission and abstracts and presentations during the trial.
The information about the TSC that will be included in published trial reports	TSC members will be named and their affiliations listed in the main report, unless they explicitly request otherwise.

For peer review only - http://bmjopen.bmj.com/site/about/guidelines.xhtml

Abbreviations and glossary

AE	Adverse event
CF	Consent form
CI	Chief Investigator
CRF	Case Report Form
CTA	Clinical Trials Authorisation
CTU	Clinical Trials Unit
DMC	Data Monitoring Committee
HE	Health Economics
ISRCTN	International standard randomised controlled trial number
MRC	Medical Research Council
NHS	National Health Service
PI	Principal Investigator
PIS	Patient information Sheet
QL	Quality of life
SAE	Serious adverse event
SOP	Standard operating procedures
SSA	Site specific assessment
TMG	Trial Management Group
TSC	Trial Steering Committee

For peer review only - http://bmjopen.bmj.com/site/about/guidelines.xhtml

Annexe 1: Agreement and competing interests form for independent members

TIP Trial Steering Committee: Agreement to join the Trial Steering Committee as an independent member and disclosure of potential competing interests

Please complete the following document and return to the TSC Facilitator.

(please initial box to agree)

☐ I have read and understood the TSC Charter version 1.0, dated 22 April 2022

☐ I agree to join the Trial Steering Committee for this trial as an independent member

☐ I agree to treat all sensitive trial data and discussions confidentially

The avoidance of any perception that independent members of a TSC may be biased in some fashion is important for the credibility of the decisions made by the TSC and for the integrity of the trial.

Potential competing interests should be disclosed via the via the Primary Care Research Centre. In many cases simple disclosure up front should be sufficient. Otherwise, the (potential) independent TSC member should remove the conflict or stop participating in the TSC. **Table 1** lists potential competing interests.

☐ **No,** I have no potential competing interests to declare

☐ **Yes,** I have potential competing interests to declare (please detail below)

Please provide details of any potential competing interests:

____

____

____

Name: ________________________

Signed: ________________________ Date: ____________

Table 1: Potential competing interests for independent members

- Stock ownership in any commercial companies involved
- Stock transaction in any commercial company involved (if previously holding stock)
- Consulting arrangements with the Sponsor/Funder
- Ongoing advisory role to a company providing drugs to the trial
- Frequent speaking engagements on behalf of the intervention
- Career tied up in a product or technique assessed by trial
- Hands-on participation in the trial
- Involvement in the running of the trial
- Emotional involvement in the trial
- Intellectual conflict e.g. strong prior belief in the trial's experimental arm
- Involvement in regulatory issues relevant to the trial procedures
- Investment (financial or intellectual) or career tied up in competing products
- Involvement in the writing up of the main trial results in the form of authorship

Note: This TSC charter was developed using MRC CTU template TSC Charter version 1.02, 13-Mar-2006

For peer review only - http://bmjopen.bmj.com/site/about/guidelines.xhtml

Annexe 2: Agreement and competing interests form for non-independent members

<u>TIP Trial Steering Committee:</u> Agreement to join the Trial Steering Committee as an independent member and disclosure of potential competing interests

Please complete the following document and return to the Facilitator.

(please initial box to agree)

☐	I have read and understood the TSC Charter version 1.0, dated 22 April 2022
☐	I agree to join the Trial Steering Committee for this trial as an non-independent member
☐	I agree to treat all sensitive trial data and discussions confidentially

The avoidance of any perception that members of a TSC may be biased in some undisclosed fashion is important for the credibility of the decisions made by the TSC and for the integrity of the trial.

Possible competing interests should be disclosed via the Primary Care Research Centre. In many cases simple disclosure up front should be sufficient. Otherwise, the (potential) independent TSC member should remove the conflict or stop participating in the TSC. Table 1 lists potential competing interests.

☐ **No,** I have no competing interests to declare other than involvement in the trial
☐ **Yes,** I have competing interests to declare (please detail below)

Please provide details of any competing interests:

____
____
____

Name: ____________________________

Signed: ___________________________ Date: _______________

Table 1: Potential competing interests for non-independent members

- Stock ownership in any commercial companies involved
- Stock transaction in any commercial company involved (if previously holding stock)
- Consulting arrangements with the Sponsor/Funder
- Ongoing advisory role to a company providing drugs to the trial
- Frequent speaking engagements on behalf of the intervention
- Intellectual conflict e.g. strong prior belief in the trial's experimental arm
- Involvement in regulatory issues relevant to the trial procedures
- Investment (financial or intellectual) in competing products

Note: This TSC charter was developed using MRC CTU template TSC Charter version 1.02, 13-Mar-2006

For peer review only - http://bmjopen.bmj.com/site/about/guidelines.xhtml

Annexe 3: Agreement and confidentiality agreement for observers

TIP Trial Steering Committee: Agreement to attend the Trial Steering Committee and treat all information confidentially

Please complete the following document and return to the Facilitator.

(please initial box to agree)

☐	I have received a copy of the TSC Charter version 1.0 22 April 2022
☐	I agree to attend the Trial Steering Committee meeting on ___/____/______
☐	I agree to treat as confidential any sensitive information gained during this meeting unless explicitly permitted

Name: ____________________________

Signed: ___________________________ Date: _______________

Note: This TSC charter was developed using MRC CTU template TSC Charter version 1.02, 13-Mar-2006

For peer review only - http://bmjopen.bmj.com/site/about/guidelines.xhtml

Annexe 4: Summarise changes from previous version

Version 1.0

This is version 1.0 of the TSC charter for this trial. There are no changes to be reported.

For peer review only - http://bmjopen.bmj.com/site/about/guidelines.xhtml

Online Supplementary File 5

Stop-Go Progression Criteria

Progression criteria are based on recruitment rates 6 months after commencing patient recruitment:

• GREEN: Recruited 21 practices and 420 patients, with a good pipeline. Continue as planned.

• AMBER: Recruited 15-20 practices and at least 150 patients, with a good pipeline. Discuss with TSC and funder possible mitigating actions, e.g., increase staff time on recruitment activities, expand to other CRNs, shorten patient follow-up period.

• RED: Recruit <15 practices and <150 patients. Discuss with TSC and funder to explore all possible avenues to save the trial. If none deemed feasible, then stop.

For peer review only

Online Supplementary File 6

Plans to give access to the full protocol, participant level-data and statistical code

The protocol will be published in an open access journal. We will seek patient and practitioner consent to deposit data in a data archive e.g., for secondary analysis. For participants who consent for their data to be deposited in a data archive, we will take the necessary steps to pseudonymize the data prior to deposit. Data will be deposited in Pure, the University of Southampton's online data repository, where access will be restricted through gatekeepers (the chief investigators) to suitably qualified individuals with appropriate protocols in place. Statistical code will not be deposited as the pseudonymisation process alters the dataset in a way that impacts the applicability of the statistical code.

For peer review only

BMJ Open

Talking in Primary Care (TIP): Protocol for a cluster-randomised controlled trial in UK primary care to assess clinical and cost effectiveness of communication skills e-learning for practitioners on patients' musculoskeletal pain and enablement

Journal:	*BMJ Open*
Manuscript ID	bmjopen-2023-081932.R1
Article Type:	Protocol
Date Submitted by the Author:	23-Feb-2024

Complete List of Authors:	Bishop, Felicity; University of Southampton, Psychology<br>Cross, Nadia; University of Southampton, Primary Care Research Centre, School of Primary Care, Population Science, and Medical Education<br>Dewar-Haggart, Rachel; University of Southampton, School of Psychology and Primary Care Research Centre, School of Primary Care, Population Science, and Medical Education<br>Teasdale, Emma; University of Southampton, School of Psychology and Primary Care Research Centre, School of Primary Care, Population Science, and Medical Education<br>Herbert, Amy; University of Bristol, Centre of Academic Primary Care, Bristol Medical School<br>Robinson, Michelle; Keele University, School of Primary, Community and Social Care<br>Ridd, Matthew; University of Bristol Faculty of Health Sciences, Population Health Sciences<br>Mallen, Christian; Keele University, Keele School of Medicine<br>Clarson, Lorna; Keele University, Keele School of Medicine<br>Bostock, Jennifer; University of Southampton, Primary Care Research Centre, School of Primary Care, Population Science, and Medical Education<br>Becque, Taeko; University of Southampton, Primary Care Research Centre, School of Primary Care, Population Science, and Medical Education<br>Stuart, Beth; Queen Mary University of London, Wolfson Institute of Population Health<br>Garfield, Kirsty; University of Bristol, Bristol Randomised Trials Collaboration<br>Morrison, Leanne; University of Southampton School of Psychology, Primary Care Research Centre, School of Primary Care, Population Science, and Medical Education,<br>Pollet, Sebastien; University of Southampton, School of Psychology and Primary Care Research Centre, School of Primary Care, Population Science, and Medical Education<br>Vennik, Jane; University of Southampton, Primary Care Research Centre, School of Primary Care, Population Science, and Medical Education<br>Atherton, Helen; University of Warwick<br>Howick, Jeremy; University of Leicester, Leicester Medical School;

	University of Oxford, Faculty of Philosophy<br>Leydon, Geraldine; University of Southampton, Primary Care Research Centre, School of Primary Care, Population Science, and Medical Education<br>Nuttall, Jacqui; University of Southampton, Southampton Clinical Trials Unit<br>Islam, Nazrul; University of Southampton, Primary Care Research Centre, School of Primary Care, Population Science, and Medical Education<br>Lee, Paul; University of Southampton, Southampton Clinical Trials Unit; University Hospital Southampton NHS Foundation Trust, Southampton Clinical Trials Unit<br>Little, Paul; University of Southampton, Primary Care Research Centre, School of Primary Care, Population Science, and Medical Education<br>Everitt, Hazel; University of Southampton, Primary Care Research Centre, School of Primary Care, Population Science, and Medical Education

**Primary Subject Heading**:	Communication
Secondary Subject Heading:	General practice / Family practice, Health services research, Medical education and training, Patient-centred medicine, Rehabilitation medicine
Keywords:	Primary Health Care, eHealth, MEDICAL EDUCATION & TRAINING, Musculoskeletal disorders < ORTHOPAEDIC & TRAUMA SURGERY, Patient-Centered Care

SCHOLARONE™
Manuscripts

Talking in Primary Care (TIP): Protocol for a cluster-randomised controlled trial in UK primary care to assess clinical and cost effectiveness of communication skills e-learning for practitioners on patients' musculoskeletal pain and enablement

For peer review only

Authors

Hazel Everitt, Nadia Cross, Rachel Dewar-Haggart, Emma Teasdale, Amy Herbert, Michelle Robinson, Matthew J Ridd, Christian Mallen, Lorna Clarson, Jennifer Bostock, Taeko Becque, Beth Stuart, Kirsty Garfield, Leanne Morrison, Sebastien Pollet, Jane Vennik, Helen Atherton, Jeremy Howick, Geraldine M Leydon, Jacqui Nuttall, Nazrul Islam, Paul H Lee, Paul Little, Felicity L Bishop*.

*Corresponding Author

Professor Felicity L Bishop, School of Psychology, University of Southampton, Highfield Campus, Southampton, UK, SO17 1BJ. Email F.L.Bishop@southampton.ac.uk. Phone +44 (0)23 80599020. Fax N/A.

For peer review only - http://bmjopen.bmj.com/site/about/guidelines.xhtml

Author Details and Affiliations

Name	Institution Affiliation	City	Country
Felicity L Bishop	School of Psychology, University of Southampton	Southampton	UK
Hazel A Everitt	Primary Care Research Centre, School of Primary Care, Population Science, and Medical Education, University of Southampton	Southampton	UK
Paul Little	Primary Care Research Centre, School of Primary Care, Population Science, and Medical Education, University of Southampton	Southampton	UK
Geraldine M Leydon	Primary Care Research Centre, School of Primary Care, Population Science, and Medical Education, University of Southampton	Southampton	UK
Beth Stuart	Wolfson Institute of Population Health, Queen Mary University of London	London	UK
Leanne Morrison	School of Psychology and Primary Care Research Centre, School of Primary Care, Population Science, and Medical Education, University of Southampton	Southampton	UK
Jane Vennik	Primary Care Research Centre, School of Primary Care, Population Science, and Medical Education, University of Southampton	Southampton	UK
Christian Mallen	Keele School of Medicine, Keele University	Keele	UK
Lorna Clarson	Keele School of Medicine, Keele University	Keele	UK
Matthew Ridd	Centre of Academic Primary Care, Bristol Medical School, University of Bristol	Bristol	UK
Kirsty Garfield	Health Economics Bristol, Population Health Sciences, Bristol Medical School, University of Bristol	Bristol	UK
Jeremy Howick	Leicester Medical School, University of Leicester; Faculty of Philosophy, University of Oxford	Leicester	UK
Helen Atherton	Unit of Academic Primary Care, Warwick Medical School	Warwick	UK
Jennifer Bostock	N/A	London	UK
Nadia Cross	Primary Care Research Centre, School of Primary Care, Population Science, and Medical Education, University of Southampton	Southampton	UK
Emma Teasdale	School of Psychology and Primary Care Research Centre, School of Primary Care, Population Science, and Medical Education, University of Southampton	Southampton	UK
Sebastien Pollet	School of Psychology and Primary Care Research Centre, School of Primary Care, Population Science, and Medical Education, University of Southampton	Southampton	UK
Rachel Dewar-Haggart	School of Psychology and Primary Care Research Centre, School of Primary Care, Population Science, and Medical Education, University of Southampton	Southampton	UK
Jacqui Nuttall	Southampton Clinical Trials Unit, University of Southampton and University Hospital Southampton NHS Foundation Trust	Southampton	UK
Nazrul Islam	Primary Care Research Centre, School of Primary Care, Population Science, and Medical Education, University of Southampton	Southampton	UK
Paul H Lee	Southampton Clinical Trials Unit, University of Southampton and University Hospital Southampton NHS Foundation Trust	Southampton	UK

For peer review only - http://bmjopen.bmj.com/site/about/guidelines.xhtml

Taeko Becque	Primary Care Research Centre, School of Primary Care, Population Science, and Medical Education, University of Southampton	Southampton	UK
Amy Herbert	Centre of Academic Primary Care, Bristol Medical School, University of Bristol	Bristol	UK
Michelle Robinson	Keele School of Medicine, Keele University	Keele	UK

Keywords

Primary Health Care; Empathy; Optimism; Health Communication; Digital Technology.

Word Count

Word count = 4675

For peer review only

For peer review only - http://bmjopen.bmj.com/site/about/guidelines.xhtml

Abstract

Introduction

Effective communication can help to optimise healthcare interactions and patient outcomes. However, few interventions have been tested clinically or subjected to cost-effectiveness analysis or are sufficiently brief and well-described for implementation in primary care. This paper presents the protocol for determining the effectiveness and cost-effectiveness of a rigorously developed brief eLearning tool, EMPathicO, among patients with and without musculoskeletal pain.

Methods and Analysis

A cluster randomised controlled trial in GP surgeries in England and Wales serving patients from diverse geographic, socio-economic, and ethnic backgrounds. GP surgeries randomised (1:1) to receive EMPathicO e-learning immediately, or at trial end. Eligible practitioners (e.g., GPs, physiotherapists, nurse practitioners) are involved in managing primary care patients with musculoskeletal pain. Patient recruitment managed by practice staff and researchers. Target recruitment is 840 adults with and 840 without musculoskeletal pain consulting face-to-face, by telephone or video. Patients complete web-based questionnaires at pre-consultation baseline, 1-week and 1-, 3- and 6-months later. Two patient-reported primary outcomes – pain intensity and patient enablement. Cost-effectiveness considered from NHS and societal perspectives. Secondary and process measures include practitioner patterns of use of EMPathicO, practitioner-reported self-efficacy/intentions, and patient-reported: symptom severity, quality of life, satisfaction, perceptions of practitioner empathy and optimism, treatment expectancies, anxiety, depression, continuity of care. Purposive sub-samples of patients, practitioners, and practice staff take part in up to two qualitative semi-structured interviews.

Ethics Approval and Dissemination

Approved by South Central – Hampshire B Research Ethics Committee on 1.7.22 and Heath Research Authority and Health and Care Research Wales on 6.7.22 (REC reference 22/SC/0145; IRAS project ID 312208). Results will be disseminated via peer-reviewed academic publications, conference presentations and patient and practitioner outlets. If successful, EMPathicO could quickly be made available at low cost to primary care practices across the country.

Registration

ISRCTN18010240 registered 15 September 2022.

Strengths and Limitations of this Study

- Assessment of a brief online learning package which is evidence and theory-based and was rigorously developed with primary care clinicians.
- Practitioners (e.g., GPs, Physios, Nurses) consult as usual without needing to identify or consent patients within the consultation, as patient recruitment is done by administrative staff.
- Focussed on patients with musculoskeletal pain but including other patients as ‘all-comers’ enables efficient test of relevance to all primary care consultations.
- Feasibility work showed it is not practicable to record consultations in this trial, so there is no direct assessment of changes in practitioner communication behaviours after engaging with the e-learning package.

For peer review only - http://bmjopen.bmj.com/site/about/guidelines.xhtml

- ‘All-comers’ is a large and varied group of patients which enhances generalisability but is not suitably powered to plan sub-group analyses.

For peer review only

For peer review only - http://bmjopen.bmj.com/site/about/guidelines.xhtml

Introduction

Approximately 1.7 billion people worldwide have musculoskeletal conditions, which are typically painful, limit peoples' daily lives, and impair quality of life.[1] Musculoskeletal conditions including back, hip, knee and neck pain are commonly managed in primary care,[2-4] where patient-centred care, including excellent practitioner-patient communication, is an international priority.[5-7] In the UK, people with musculoskeletal conditions may be seen in primary care by GPs, practice nurses, physiotherapists, and other allied healthcare professionals.

Regardless of which treatment, therapy, or other intervention a patient receives, effective practitioner-patient communication can reduce symptoms and enhance quality of life, adherence to and satisfaction with care, producing benefits comparable to many pharmaceutical interventions.[8-10] Sub-optimal communication can lead to missed opportunities for benefit, worse quality of life and symptom management, unwanted prescriptions and non-adherence;[11,12] unnecessary economic costs;[12] deviation from guideline-recommended treatment;[13] and increased complaints and litigation.[14,15] Despite communication skills being taught in medical and allied health professional training, patients still report dissatisfaction with practitioner-patient communication,[16,17] the extent to which patients rate their practitioners as being empathic varies widely,[18] and medical students appear to exhibit broadly stable or declining levels of empathy during their degrees.[19,20] The need to enhance and expand communication skills is particularly pertinent since the COVID pandemic forced rapid introduction of remote consultations, bringing new opportunities and challenges for patients and staff not specifically trained to consult in this way.[21]

We focus on the communication of clinical empathy and positive messages within primary care consultations. Clinical empathy and positive messages are not routinely reliably optimised in clinical care but can have statistically and likely clinically significant effects on pain, patient satisfaction, and other outcomes with no evidence of adverse effects.[22] Our intervention planning determined that enhancing practitioners' communication of clinical empathy and realistic optimism was feasible, measurable, and likely to have significant impact.[23,24] Even brief interventions can improve communication skills, including interventions concentrating on empathy skills such as active listening and expressing warmth at appropriate times[25-27] which take no additional time in the consultation.[27,28] However, few interventions have been tested clinically for effects on patients' health,[29] have been subjected to formal cost-effectiveness evaluations,[30] or are sufficiently brief and well-described to facilitate implementation in the current primary care climate. Our work aims to address these limitations. We are evaluating the effects on patients' health of brief, evidence-based, online training to enhance practitioners' communication of clinical empathy and realistic optimism within everyday clinical consultations ("EMPathicO").

Aims and Objectives

The primary objective is to determine EMPathicO's effects on (a) patient-reported pain and (b) patient enablement via repeated measures over 6 months following the index consultation, in patients presenting with musculoskeletal pain, compared to usual care control.

This clinical focus on musculoskeletal pain was chosen to align with the EMPathicO training, which includes modules on clinical empathy, realistic optimism, and how to communicate these better in the context of consultations for osteoarthritis. Including a condition-specific module permitted clear demonstration of communication skills in a particular context, which made the training better targeted and potentially more effective.[31] A painful musculoskeletal condition was chosen because much (but not all) of the evidence

For peer review only - http://bmjopen.bmj.com/site/about/guidelines.xhtml

that underpins the importance of clinical empathy and realistic optimism for patient outcomes is derived from studies of pain and painful conditions; osteoarthritis was chosen because it is a prevalent painful musculoskeletal condition in primary care.

Secondary objectives are:

- To estimate EMPathicO's cost-effectiveness and effects on patient-reported quality of life and other secondary outcomes, over 6 months from index consultation, in patients with musculoskeletal pain.
- To test hypothesised mechanisms of action.
- To explore EMPathicO's potential for implementation, by:
    - Determining EMPathicO's effects on patient enablement, patient-reported quality of life and other secondary outcomes over 6 months from index consultation, in patients ineligible for the musculoskeletal pain group (i.e., presenting with other symptoms and/or very low levels of musculoskeletal pain, hereafter referred to as 'all-comers'). This group was included because clinical empathy and realistic optimism may be beneficial for many different symptoms seen in primary care, and when practitioners adopt new communication behaviours within consultations for one type of condition these skills may 'spill-over' and also be implemented in consultations for other conditions. We wanted to evaluate any such additional benefits.
    - Identifying opportunities, barriers, and solutions for widespread implementation and impact, using the RE-AIM framework to explore EMPathicO's Reach, Effectiveness, Adoption, Implementation, and Maintenance. [32,33]

Methods and Analysis

This protocol reported in accordance with the Standard Protocol Items: Recommendations for Interventional Trials (SPIRIT) checklist (Supplemental Material 1).[34] The first site was randomised on 31.10.22 and data collection is due to finish on 31.7.24.

Patient and Public Involvement and Engagement (PPIE)

To ensure our work engages and is relevant to patients, we have worked with patients and members of the public throughout developing EMPathicO and this protocol. We continue working closely with our Patient Advisory Group, led by our PPIE lead, JB, who sits on our trial management group. This group comprises six patient and public contributors of varying ages, ethnic backgrounds (three from Black and Minority Ethnic backgrounds, three from White backgrounds), gender (three female, three male), and geographical locations within England. One member is neurodivergent, and all have lived experience of MSK pain as patients or carers. Our panel meet virtually for one hour bimonthly and contribute to specific activities including refining patient-facing documents and procedures, training qualitative interviewers, and interpreting data.

Design

A cluster-randomised controlled parallel group superiority trial in primary care, with embedded qualitative and mixed methods process and implementation analyses.

For peer review only - http://bmjopen.bmj.com/site/about/guidelines.xhtml

Cluster randomisation was chosen because randomising individual practitioners risks cross-contamination within practices where practitioners share knowledge and patients; randomising individual patients risks contamination because practitioners cannot switch on/off communication skills in different consultations.

General practices constitute the clusters; practices are recruited and then randomised 1:1 EMPathicO: control. Randomisation is stratified (see below). All eligible practitioners within clusters are encouraged to undertake EMPathicO training (intervention) or consult patients as usual (control). The control was chosen to enable pragmatic assessment of benefits and costs of adding EMPathicO training to usual care.

Patient recruitment commences at least two weeks after the general practice is randomised (enabling time for intervention sites to complete the intervention training whilst maintaining consistent set up timelines across both arms). All adults (18+) verbally consulting a participating practitioner are invited to participate in the trial (see exclusions below).

Two groups of patients are recruited. The musculoskeletal group comprises patients consulting participating practitioners about musculoskeletal pain. The 'all-comer' group comprises patients consulting about symptoms other than musculoskeletal pain (or reporting very low levels of musculoskeletal pain). At pre-consultation baseline and repeatedly up to 6 months later patients complete questionnaires assessing pain, enablement, and secondary outcomes.

Setting

General practices in England and Wales, recruited and supported by three recruitment hubs – Southampton, Keele, and Bristol.

Target population

GP Practice Eligibility Criteria

Eligible: NHS general practices in England and Wales, where a general practice is "an organisation which offers Primary Care medical services by a qualified General Practitioner who can prescribe medicine and where patients can be registered and held on a list."[35]

Excluded: Practices involved in intervention development/feasibility work (18 from Wessex, 5 from West Midlands), practices where clinical members of the Trial Management Group/Trial Steering Committee see patients.

Practitioner Eligibility Criteria

Eligible: practitioners from any discipline who are working within participating GP surgeries and seeing patients with musculoskeletal pain (e.g., GPs, Practice Nurses, Physiotherapists, Pharmacists, Physician Associates).

Excluded: Practitioners unwilling to undertake the intervention/trial procedures.

Patients with Musculoskeletal Pain Eligibility Criteria

For the musculoskeletal pain group, eligible patients are adults (18+); verbally consulting a participating practitioner about new, recurrent, or ongoing musculoskeletal pain (e.g. back, hip, upper/lower extremity, neck pain - consistent with ICD-11's diseases of the musculoskeletal system[36]); reporting average pain in

For peer review only - http://bmjopen.bmj.com/site/about/guidelines.xhtml

the last week as 4 or more on numerical rating scale at baseline (0 = no pain; 10 = pain as bad as you can imagine); consulting face-to-face , telephone, or videoconference; able to give informed consent. The first consultation is the 'index' consultation, an initial triage interaction does not constitute an 'index' consultation. People without English as a first language are eligible, interpreters are available to support access to trial paperwork and patient-reported measures, and their use is recorded; informal interpreters (e.g., family) may also support.

Excluded: patients consulting solely in written forms (e.g., e-consult/email); pain caused by malignancy; unable to consent or to complete questionnaires (e.g., severe mental illness or distress, terminal illness); already enrolled in the trial (i.e., from a previous consultation); aged <18.

All-Comer Patients Eligibility Criteria

For the all-comers group, eligible patients are adults (18+); verbally consulting a participating practitioner about something other than musculoskeletal pain or consulting for musculoskeletal pain and rating average pain in last week as less than 4 at baseline; able to give informed consent.

Excluded: As for patients with musculoskeletal pain.

Interventions

EMPathicO e-Learning Package

EMPathicO is an evidence-based theoretically-grounded digital e-learning package for practitioners routinely seeing patients frontline in primary medical care, including GPs, nurse practitioners and first-contact physiotherapists.[24] EMPathicO helps practitioners enhance their communication of clinical empathy and realistic optimism, is consistent with major consultation models including 'ICE' (Ideas, Concerns and Expectations),[37] and incorporates behaviour change techniques. Using the Behaviour Change Wheel, EMPathicO was designed to target users' motivation (reflective, autonomic), capability (physical, psychological), and opportunity (environmental), through intervention functions of persuasion, incentivization, enablement, education, training, modelling, and environment restructuring. Multiple Behaviour Change Techniques were used to achieve these functions, including demonstration, information provision, goal-setting, action planning, and instruction. For a complete behavioural analysis of EMPathicO see supplementary material in our intervention development paper.[24]

The brief interactive e-learning modules are completed by practitioners and can be completed separately or together in less than 75 minutes and cover clinical empathy, realistic optimism, tailoring empathy and optimism for patients with osteoarthritis (a common cause of musculoskeletal pain), evaluating one's own consultations, and goal-setting. Figure 1 summarises the structure and contents of the modules. EMPathicO was developed using LifeGuide open-source software for creating online interventions for health care, health promotion and training.[38]

---Insert Figure 1 Here---

The systematic process of developing EMPathicO using the person-based approach[39] involved multiple literature reviews, behavioural analysis, and extensive iterative qualitative research.[40-46] This work all contributed to the underpinning logic model (Figure 2).[24]

For peer review only - http://bmjopen.bmj.com/site/about/guidelines.xhtml

---Insert Figure 2 Here---

Control: Usual Care

Practitioners in practices randomised to usual care control do not receive training and are asked to consult as usual. They are offered access to EMPathicO after all patient recruitment and follow-up is completed.

Concomitant Interventions

All practitioners are discouraged from undertaking additional communication skills training during the study and must self-report any that does occur.

For peer review only

Recruitment

GP Practice Recruitment

Practices are recruited with local Clinical Research Network (CRN) support, seeking practices of different sizes (small-large) and locations (urban, rural) and those serving populations in areas of higher deprivation and greater ethnic diversity.

Practitioner Recruitment

Practitioners within participating practices are recruited by that practice's lead for this study (the local PI) with support from the trial team and materials including an infographic and one-minute video explaining the study.

Patient Recruitment

Practices invite consecutive patients consulting participating practitioners within the recruitment period, after screening out any patients who do not have capacity for consent, or where there are medical grounds for excluding the patient (e.g., very unwell generally, severe mental distress). Patient recruitment methods are tailored to suit individual practices' appointment booking systems. For patients with prebooked or same-day appointments, practices text, email, or post a brief invitation and link to the patient-facing study website up to 1 week before their consultation. Practices screen potential invitees for initial eligibility before sending invitations. Practices may display a poster in practice and/or on their website. Reception staff may introduce the study to patients attending in-person. Patients email or phone the patient-facing research team with questions.

Practices follow their usual procedures for contacting non-English speakers to invite them to take part e.g., contacting a designated friend, relative or support worker, arranging an interpreter, or adding a sentence in the patient's own language on the initial study invitation.

The number of patient invitation emails/texts sent by each site is collected and recorded centrally. Qualtrics records instances of patients accessing the study website but declining consent and/or not meeting inclusion criteria.

The patient-facing study website is hosted on Qualtrics and shows the full study invitation and patient information sheet (PIS) (in languages requested by practices). After reading the PIS, patients complete a

For peer review only - http://bmjopen.bmj.com/site/about/guidelines.xhtml

brief screening questionnaire, online consent and baseline measures. Supplemental Material 2 contains PIS and consent forms.

Sample size

Patients with Musculoskeletal Pain Sample Size

The minimum clinically important difference in the pain primary outcome is approximately one point,[47] standard deviation 3.3, consistent with a standardised effect size of 0.3. For 90% power, alpha of 0.025 to allow for two primary outcomes, and a correlation between the 4 repeated measures of 0.7, a sample size of 214 per group is required. We assume a conservative ICC of 0.03, at the upper 75% of what has been observed in previous primary care trials.[48] Assuming 20 patients per practice gives a design effect of 1.57. Allowing for 20% loss to follow up gives a total sample size of (214*2*1.57)/0.8=840 participants to be recruited from 42 practices.

'All-Comer' Patients Sample Size

Recruiting 840 all-comers will give 90% power (based on alpha and ICC as per the musculoskeletal group above) to detect a standardised effect size of 0.3 in the enablement primary outcome, equivalent to a difference of 0.36 points (assuming SD=1.2[49]).

Updated sample size calculation

Participants are being recruited from 53 practices rather than 42 practices as originally planned, which reduces the average cluster size. Assuming 14 patients per practice gives a design effect of 1.39. Under the same assumptions as above, the total sample size is (214*2*1.39)/0.8=744 participants.

Outcomes

Questionnaires, Data Collection and Participant Retention

Supplemental Material 3 summarises outcome and process variables, measurement timings, and questionnaire measures. We considered core outcome sets, questionnaire properties (e.g., validity, reliability, length), and acceptability to participants when choosing specific measures.

Patient-reported measures are completed on web-based questionnaires hosted on Qualtrics (Qualtrics, Provo, UT); to support inclusive access patients may request an interpreter and/or paper versions. £10 vouchers are sent at 1-month and 6-month follow-ups to incentivize completion.

Practitioner-reported measures are completed on LifeGuide[38] (measures completed by intervention group only) and Qualtrics (measures completed by all practitioners).

For practitioners and patients, automated follow-up emails are sent to non-responders at all timepoints. Researchers personally contact persistent non-responders who haven't withdrawn and offer to resend questionnaires or complete primary outcomes by telephone.

Primary Outcomes

For the musculoskeletal pain group, the two primary outcomes are pain intensity and patient enablement, each analysed over 6 months using a repeated measures approach. Pain intensity is the severity of pain sensation and is included in core outcome sets for chronic pain,[50 51] OA,[52] and low back pain.[53,54]

For peer review only - http://bmjopen.bmj.com/site/about/guidelines.xhtml

Patient enablement refers to patients' feelings, after a consultation, of confidence and empowerment to cope with their symptoms, to keep healthy and to help themselves. Our PPIE work highlighted enablement as at least as important as pain. Two primary outcomes help capture more holistic effects on patients' health. The outcomes will be reported separately and our PPIE and embedded qualitative work will help explore, interpret and explain how they relate to each other.

For the all-comers group, patient enablement is the single primary outcome. Pain intensity is measured as a secondary outcome if pain is present.

Pain Intensity

Pain intensity is measured as average pain in the last week using the 4-item pain intensity subscale from the Brief Pain Inventory (BPI).[55]

Patient Enablement

The 6-item Patient Enablement Index (PEI) captures patients' feelings, after a consultation, of confidence and empowerment to cope with their symptoms, to keep healthy and to help themselves.[56] To increase sensitivity, versions with more response options than the original four (much better/never/same or less/not applicable) have been reported.[57-59] Following our feasibility study we use a modified 7-point agree-disagree Likert response scale with a Not Applicable option.

Secondary Outcomes

Symptom Severity and Global Impression of Change

Overall perceptions of symptom severity and change are important for musculoskeletal patients given the high prevalence of multi-morbid conditions and for all-comers because they apply to any condition and provide a symptom-focused pre-consultation baseline. Two single item 7-point[60] measures of Patient Global Impression of Symptom Severity and Patient Global Impression of Change are collected.[61]

Patient Satisfaction

The version of the 21-item Medical Interview Satisfaction Scale[62] (MISS) adapted and revalidated for UK primary care[63] is used to measure patient satisfaction with the consultation.

Pain Interference

Pain interference is measured with the 7-item pain interference scale from the BPI.[55]

Health-Related Quality of Life

Health status is measured using the 5-item EQ-5D-5L and the EQ-VAS.[64]

Health Economics Outcomes

Cost effectiveness will be assessed from NHS and societal perspectives including personal expenses and productivity over 6 months. Utility values will be estimated from EQ-5D-5L scores using the NICE-recommended approach at the time of analysis. Quality-adjusted life-years will be estimated by combining utility values, with length of time in each health state, using the area under the curve approach.[64-66] The

For peer review only - http://bmjopen.bmj.com/site/about/guidelines.xhtml
For peer review only

5-item ICECAP-A, which was designed to capture broader aspects of quality-of-life and has been found to complement the EQ-5D in economic evaluations, is also collected.[67,68]

Practitioner time spent on EMPathicO training is captured by LifeGuide. Resource-use data is collected using ModRUM[69] (patient self-reported healthcare utilization) and bespoke questions (costs outside the healthcare sector e.g., personal expenses). The Work Productivity and Activity Impairment Questionnaire: General Health is used to collect information on productivity, including time off work.[70] NHS resources include primary, community and secondary care, and prescribed medications; they will be valued using the national unit costs.[71-73] Personal expenses will be presented as reported. Sick leave from employment will be valued using Annual Survey of Hours and Earnings.[74]

Process Variables and Covariates

Potential mediators and moderators of intervention effects on pain, specified in the logic model, are included as process variables. Practitioner-reported self-efficacy, outcome expectancy, and intentions for conveying empathy and optimism in consultations are assessed using bespoke items developed in our feasibility work based on standard item stems, relevant guidelines and theory.[75-78] They demonstrated acceptable internal consistency (Cronbach's alphas ranged 0.69-0.98) and were fully completed by practitioners (n=11).

Intervention usage data captured on LifeGuide includes, for each practitioner-participant, time spent on (different sections of) the intervention and patterns of access.

Patient perceptions of practitioner clinical empathy are assessed using the 10-item CARE[79] used extensively in UK primary care settings to assess patient perceptions of clinical empathy. Patient perceptions of practitioner response expectancies are assessed using a bespoke single item tested in our feasibility study. Patient treatment outcome expectancies are measured using the 15-item 6-subscale, Treatment Expectation Questionnaire (TEX-Q).[80] Patient anxiety and depression are assessed using the 7-item subscales from the Hospital Anxiety and Depression Scale (HADS).[81,82] Continuity of care is assessed using the 9-item Patient-Doctor Depth of Relationship Scale,[83] modified for non-doctor practitioners.

Practitioner characteristics collected are age, gender, ethnicity, years qualified, profession. Practice-level data collected from the practice and supplemented with data from national general practice profiles (National General Practice Profiles - Data – OHID, phe.org.uk) are: list size, deprivation score, staffing.

Patient characteristics collected are age, gender, ethnicity, postcode (for calculating index of multiple deprivation, IMD), reason(s) for consulting (coded using the ICPC-2), comorbidities, and index consultation modality.

Qualitative Interviews

A subsample of patients (up to n=45 with musculoskeletal pain and n=45 all-comers) and practitioners (up to n=40) take part in qualitative semi-structured telephone interviews. Participants are purposively sampled to capture diversity in index-consultation mode (telephone/video/face-to-face), ethnicity, age, gender, baseline pain severity. Participants are interviewed twice each, to explore short-term and longer-term implementation of EMPathicO skills (practitioners) and experiences of the index and subsequent consultations (patients). Practitioners are interviewed after (1) patient recruitment and (2) follow-up is completed at their practice. Patients are interviewed within approximately 7-14 days of their index

For peer review only - http://bmjopen.bmj.com/site/about/guidelines.xhtml

consultation and again approximately 6 months later. Topic guides comprising open-ended questions and prompts are used flexibly and modified iteratively as necessary to explore emerging avenues of inquiry within scope of the trial. Field notes are taken, interviews are transcribed verbatim, identifying details are replaced (e.g., using pseudonyms), and transcripts are checked and imported to NVivo (Lumivero, Denver, CO) for analysis.

Timelines

Tables 1 and 2 show practitioner and patient timelines for enrolment, questionnaires, and interviews.

---Insert Tables 1 and 2 Here ---

Assignment of Interventions

Sequence Generation, Allocation Concealment and Implementation

A computer-generated allocation sequence is used with random block sizes of 4 and 6. Blocks are stratified by practice-level high/low deprivation (IMD 1-5 / IMD 6-10) and large/small practice size (list size>7900 / <7900; 7900 = median practice list size in England). The allocation sequence is implemented using the randomisation function in LifeGuide and is not visible to users. The trial manager (or their delegate) inputs each eligible practice to the randomisation function on LifeGuide which then displays the allocation. Practitioners and patients can withdraw from the study without giving a reason, but they cannot request modification to their allocated intervention.

Blinding

Patients and the trial statistician are masked to intervention allocation. Patients are not told in the PIS that as part of this study their general practice has been randomly allocated to intervention or control. This was approved by the ethics committee and is appropriate in this cluster-randomised trial where the communication-skills training intervention is very low risk and within the broad scope of usual practice. After all data collection is complete, patients will be debriefed in writing (email/mail) and told that “at the start of the TIP study some of the GP practices taking part had communication skills training (intervention practices) and some GP practices did not have any training (control practices).” They will also be told whether their practice did or did not receive the enhanced communication skills training. Efforts are made to mask researchers supporting patient data collection to intervention allocation; for example, the researchers collecting patient outcomes are not the same researchers who liaise with practices about the intervention. Efforts are made to mask practitioners to which patients are taking part; for example, the patient PIS includes the instruction to “please do not discuss your participation in the study with your GP, nurse, physiotherapist, or any other primary care practitioner”. In the unlikely event that patient unblinding is deemed necessary for patient care this will be done by the general practice and notified to the research team.

For peer review only - http://bmjopen.bmj.com/site/about/guidelines.xhtml

Data Analysis

Data Management

Web-based questionnaire data stored securely on Qualtrics servers (see https://www.qualtrics.com/security-statement/). Questionnaire data collected by telephone or paper entered into Qualtrics by one researcher and checked for accuracy by a second researcher.

Personal data stored on a secure server at University of Southampton in compliance with General Data Protection Regulations and the Data Protection Act 2018.

Statistical Methods

Musculoskeletal and all-comers groups will be analysed separately. For the two primary outcomes, a linear mixed model will use all the observed data, and implicitly assumes that missing outcome scores are missing at random given the observed data. The BPI and PEI will be reported and analysed using post-intervention scores, adjusting for baseline score. The primary analyses for the BPI and PEI scores will be performed using a generalized linear mixed model (GLMM) framework with observations at 3 days, 1-. 3-, and 6-months (level 1) nested in participants (level 2) and participants nested in practices (level 3). Unadjusted results will be reported as well as results adjusting for baseline values, stratification variables and other covariates as appropriate. As there may not be a constant treatment effect over time, a treatment/time interaction will be modelled and included if significant, with time treated as a random effect. An unstructured covariance matrix will be used. For secondary outcomes, the analyses will use a similar modelling approach, with mixed logistic/linear regression models as appropriate, a random effect for practice, controlling for baseline values, stratification variables and potential confounders. No formal pre-planned subgroup analyses.

Intention to treat analysis (as randomised) will be undertaken regardless of any practice-level non-adherence to the intervention. All available data will be used, with a sensitivity analysis using multiple imputation if appropriate. Linear mixed models and multiple imputation both assume the data are missing at random, therefore sensitivity analyses to data missing not at random will also be explored. A full and detailed statistical analysis plan will be developed prior to final trial analysis and approved by Trial Steering Committee.

Interim analyses of outcomes are deemed unnecessary in this low-risk trial.

Health Economic Analysis

An NHS perspective will be taken in the primary analysis; a wider perspective is taken in secondary analyses including impacts on patients and productivity. Analysis will be intention to treat. Relevant covariates, including baseline EQ-5D-5L, potentially skewed data and the cluster design will be accounted for using appropriate regression models.[66] Cost-consequences will tabulate costs from each perspective to a range of outcomes. Cost-effectiveness will be estimated in a cost-utility analysis combining QALYs and NHS costs. The incremental net monetary benefit statistic will be presented at standard NICE thresholds and if appropriate, incremental cost-effectiveness ratios will be estimated. Uncertainty will be addressed by bootstrapping, plotting cost-effectiveness acceptability curves and in sensitivity analyses.

Process Analysis

A process analysis will focus on mechanisms of impact and test hypotheses derived from the logic model about relationships among variables, including mediators and moderators. Intervention usage data,

For peer review only - http://bmjopen.bmj.com/site/about/guidelines.xhtml

captured by LifeGuide, will be incorporated using the AMUsED framework for Analyzing and Measuring Usage and Engagement Data.[84]

Qualitative and Mixed Methods Analysis

EMPathicO's potential impact post-trial will be evaluated by using the RE-AIM framework to explore Reach, Effectiveness, Adoption, Implementation, and Maintenance. [32,33] Drawing on data from the main trial, the all-comers group and the qualitative interviews we will assess EMPathicO against the RE-AIM components using the approaches described in Table 3.

---Insert Table 3 Here---

Ethics and Dissemination

Safety, Adverse Events, and Insurance

This trial is classed as low risk following a risk assessment and there are no provisions for post-trial care. The team do not expect any adverse events (untoward medical occurrence in a trial participant) or Serious Adverse Events (that results in death, is life-threatening, requires hospitalisation or prolongation of existing hospitalisation, results in persistent or significant disability or incapacity, consists of a congenital anomaly or birth defect, or other medically important condition). However, adverse events are being collected (primarily via self-report), recorded and reported where necessary in accordance with the principles of ICH Good Clinical Practice and the requirements of the research ethics committee, sponsor, and trial steering committee.

Individual practitioners are responsible for maintaining appropriate cover with a medical defence organisation. University of Southampton insurance may also apply where the cause of harm was not due to clinical negligence.

Approvals, Oversight and Monitoring

The sponsor is the University of Southampton (rgoinfo@soton.ac.uk). Approval was received from South Central – Hampshire B Research Ethics Committee on 1.7.22 and the Heath Research Authority and Health and Care Research Wales on 6.7.22 (REC reference 22/SC/0145; IRAS project ID 312208). Protocol amendments are submitted for approval as required to the study sponsor and ethics committee and notified where necessary to all those concerned.

The Trial Steering Committee (TSC) provides trial oversight and advice through its independent Chairperson to the Trial Management Group and the funder on all aspects of the trial. The TSC assumes responsibilities of the Data Monitoring Committee and reviews information on the progress and accruing data; online Supplemental Material 4 presents the TSC Charter; Supplemental Material 5 presents stopping criteria). Annual and interim progress reports submitted to the funder.

Dissemination

Patient recruitment commenced on 16.11.2022 and is ongoing at the time of manuscript submission. Results will be communicated to participants and disseminated to academic, practitioner, and public audiences via peer-review journal articles, conferences, and other appropriate formats e.g. blogs. Our public collaborators will co-lead dissemination activities. Results will be reported in accordance with

For peer review only - http://bmjopen.bmj.com/site/about/guidelines.xhtml

CONSORT guidelines extensions for cluster-randomised trials[85] and trials of non-pharmacological interventions,[86] and the American Psychological Association Journal Article Reporting Standards for qualitative (JARS-QUAL) and mixed methods (JARS MMARS) research.[87] We will adhere to the ICMJE (https://www.icmje.org/) criteria for authorship and use the CRediT taxonomy (https://credit.niso.org/). Supplemental Material 6 summarises data access plans.

For peer review only

For peer review only - http://bmjopen.bmj.com/site/about/guidelines.xhtml

Tables and Figures

Table 1. Practitioner Timelines

	Allocation		Post-allocation (wk)					On completing patient recruitment	On completing patient follow-up
**TIMEPOINT**	**0**	**+1d**	**1**	**2**	**3-8**	**8**	**34**		
**ENROLMENT:**									
Eligibility screen	X								
Informed consent	X								
Site initiation visit	X								
Allocation		X							
**INTERVENTIONS:**									
EMPathicO training									
No training (control)									
**ASSESSMENTS:**									
Demographic and professional characteristics	X								
Self-efficacy for empathy and optimism	X					X	X		
Expectations, intentions for EMPathicO skills [1]				X		X	X		
Practitioner-reported other training						X	X		
Qualitative interview								X	X[1]
**PATIENT RECRUITMENT**									
Prepare invitations									
Recruit patients									

[1] Intervention-arm practitioners only

For peer review only

For peer review only - http://bmjopen.bmj.com/site/about/guidelines.xhtml

Table 2. Patient Timelines

	Enrol	Consultation	Post-consultation			
**TIMEPOINT**	***<-7d***	**0**	***<7d***	***+1m***	***+3m***	***+6m***
**ENROLMENT:**						
Eligibility screen	X					
Informed consent	X					
**ASSESSMENTS:**						
Primary Outcomes						
Pain intensity	X		X	X	X	X
Patient enablement			X	X	X	X
Secondary Outcomes						
Global impression of symptom severity	X		X	X	X	X
Global impression of symptom change			X	X	X	X
Pain interference				X		X
Patient satisfaction			X			
Health economics: EQ-5D & ICECAP-A	X			X		X
Adverse events				X	X	X
Healthcare utilization	X				X	X
Prescribed medications, personal expenses, productivity					X	X
Process Measures						
Perceptions of empathy			X			
Perceptions of optimism			X			
Treatment expectations			X			
Anxiety			X			
Continuity of care			X			
Depression			X			
Sociodemographic characteristics	X					
Health characteristics			X			
Qualitative interview			X			X

For peer review only

For peer review only - http://bmjopen.bmj.com/site/about/guidelines.xhtml

Table 3: Qualitative and Mixed Methods Data Analysis to Evaluate Intervention

RE-AIM	Data source	Analysis
**Reach**	Management data	Proportion and characteristics of practitioners and patients taking part. Reasons for declining.
**Effectiveness**	All-comers group	Apply analysis plan from main trial to test intervention effectiveness in all-comers group.
	Qualitative data (patients and practitioners)	Compare experiences of EMPathicO across in-person, telephone and video consultations, and for musculoskeletal pain vs other conditions (framework analysis).
**Adoption**	Management data	Proportion and characteristics of invited practices taking part. Reasons for declining.
**Implementation**	LifeGuide usage & qualitative data	Assess patterns of usage and 'effective engagement' with EMPathicO. Explore barriers and facilitators to implementation in practice, drawing on Normalization Process Theory [88] (framework analysis).
**Maintenance**	Qualitative data (patients and practitioners)	Explore opportunities to embed EMPathicO in existing training structures. Examine longer term maintenance of practitioner behaviour change and effects on patients (reflexive thematic analysis).

For peer review only

Figure Captions

Figure 1. Schematic Summary of Empathico Structure and Contents

Figure 2. Logic model showing how EMPathicO is hypothesized to affect patient outcomes.

For peer review only - http://bmjopen.bmj.com/site/about/guidelines.xhtml

Authors' Contributions

Allocated using CRediT categories. Conceptualisation (study idea) and Funding Acquisition: HE, FB, JH, PL, BS, GL, LM, JV, JB, CM, LC, MRi, KG, HA. Methodology (designing, planning and developing study methods): FB, HE, PL, GL, BS, LM, JV, CM, LC, MR, KG, JH, HA, JB, NC, ET, SP, RDH, JN, NI, PHL, TB, AH, MR. Investigation (data collection): NC, RDH, ET, AH, MRo, SP. Data Curation (study management data and data cleaning): NC, RDH, ET, AH, MRo, SP. Project Administration (managing and co-ordinating research activity plans and execution): FB, HE, NC. Software (implementation and support for the e-learning intervention): SP. Supervision (oversight, leadership, mentorship): FB, HE. Visualisation (creation and presentation of figures): SP, LM, FB. Writing (original draft): HE, FB, JH, BS, TB, MRi, KG, HA, JB. Writing (review, revisions, and editing): FB, HE, PL, GL, BS, LM, JV, CM, LC, MR, KG, JH, HA, JB, NC, ET, SP, RDH, JN, NI, PHL, TB, AH, MR.

Funding

Funding: This project is funded by the National Institute for Health Research (NIHR) School for Primary Care Research grant (project reference 563). The Primary Care Research Centre, University of Southampton is a member of the NIHR School for Primary Care Research and supported by NIHR Research funds. Service support costs will be paid by the CRN. CDM is funded by the National Institute for Health Research (NIHR) Collaborations for Leadership in Applied Health Research and Care West Midlands (grant number N/A) and the NIHR School for Primary Care Research. The EMPathicO e-learning tool was developed using LifeGuide software, which was partly funded by the National Institute for Health Research Southampton Biomedical Research Centre (BRC) (grant number N/A). The views expressed are those of the authors and not necessarily those of the NIHR or the Department of Health and Social Care.

The study sponsor (University of Southampton) and funders (NIHR SPCR) have no role in study design; collection, management, analysis, and interpretation of data; writing of the report; or the decision to submit the report for publication.

Acknowledgements

NIHR Local Clinical Research Networks (CRNs) supported practice recruitment.

Conflicts of Interest

All authors have completed the ICMJE uniform disclosure form at http://www.icmje.org/disclosure-of-interest/ and declare: financial support for the submitted work from the NIHR; CDM is Director of the NIHR School for Primary Care Research; HA has received research grants from NIHR and Research Council of Norway, payment for delivering lecture to GPs in training about remote consultations, travel expenses to attend Scientific Foundation Board meeting; HA is chair of a steering committee at University of Leeds, member of advisory boards at Imperial College London and University of Manchester, and vice-chair of the Scientific Foundation Board Royal College of General Practitioners; HA is Officer at Prof Andrew Beggs Ltd. No other relationships or activities that could appear to have influenced the submitted work.

For peer review only - http://bmjopen.bmj.com/site/about/guidelines.xhtml

References

1. Cieza A, Causey K, Kamenov K, et al. Global estimates of the need for rehabilitation based on the Global Burden of Disease study 2019: a systematic analysis for the Global Burden of Disease Study 2019. *The Lancet* 2020;396(10267):2006-17. doi: 10.1016/S0140-6736(20)32340-0
2. Jordan KP, Kadam UT, Hayward R, et al. Annual consultation prevalence of regional musculoskeletal problems in primary care: an observational study. *BMC Musculoskeletal Disorders* 2010;11(144)
3. Ruairi K. The prevalence of musculoskeletal presentations in general practice: an epidemiological study. *Br J Gen Pract* 2020;70(suppl 1):bjgp20X711497. doi: 10.3399/bjgp20X711497
4. Yu D, Missen M, Jordan KP, et al. Trends in the Annual Consultation Incidence and Prevalence of Low Back Pain and Osteoarthritis in England from 2000 to 2019: Comparative Estimates from Two Clinical Practice Databases. *Clinical Epidemiology* 2022;14(null):179-89. doi: 10.2147/CLEP.S337323
5. Van Lerberghe W. The world health report 2008: primary health care: now more than ever: World Health Organization 2008.
6. NICE NIfHaCE. Musculoskeletal Conditions Overview [Available from: https://pathways.nice.org.uk/pathways/musculoskeletal-conditions accessed 02.06.2021.
7. Lin I, Wiles L, Waller R, et al. What does best practice care for musculoskeletal pain look like? Eleven consistent recommendations from high-quality clinical practice guidelines: systematic review. *Br J Sports Med* 2020;54(2):79. doi: 10.1136/bjsports-2018-099878
8. Suarez-Almazor ME, Looney C, Liu Y, et al. A randomized controlled trial of acupuncture for osteoarthritis of the knee: Effects of patient-provider communication. *Arthritis Care Res* 2010;62(9):1229-36.
9. Haskard Zolnierek KB, DiMatteo MR. Physician communication and patient adherence to treatment: A meta-analysis. *Med Care* 2009;47(8)
10. Dambha-Miller H, Cooper AJM, Kinmonth AL, et al. Effect on cardiovascular disease risk factors of interventions to alter consultations between practitioners and patients with type 2 diabetes: A systematic review and meta-analysis of trials in primary care. *Health Expect* 2017;20(6):1218-27. doi: 10.1111/hex.12546 [published Online First: 2017/02/28]
11. Barry CA, Bradley CP, Britten N, et al. Patients' unvoiced agendas in general practice consultations: qualitative study. *Br Med J* 2000;320:1246-50.
12. Thorne SE, Bultz BD, Baile WF. Is there a cost to poor communication in cancer care?: a critical review of the literature. *Psychooncology* 2005;14(10):875-84. doi: 10.1002/pon.947
13. Moffat M, Cleland J, van der Molen T, et al. Poor communication may impair optimal asthma care: a qualitative study. *Fam Pract* 2007;24(1):65-70. doi: 10.1093/fampra/cml062
14. Stelfox HT, Gandhi TK, Orav EJ, et al. The relation of patient satisfaction with complaints against physicians and malpractice lawsuits. *The American Journal of Medicine* 2005;118(10):1126-33. doi: http://dx.doi.org/10.1016/j.amjmed.2005.01.060
15. Pincock S. Poor communication lies at heart of NHS complaints, says ombudsman. *BMJ* 2004;328(7430):10.
16. Nielsen M, Foster M, Henman P, et al. ‘Talk to us like we’re people, not an X-ray’: the experience of receiving care for chronic pain. *Australian Journal of Primary Health* 2013;19(2):138-43. doi: http://dx.doi.org/10.1071/PY11154
17. Teh CF, Karp JF, Kleinman A, et al. Older People's Experiences of Patient-Centered Treatment for Chronic Pain: A Qualitative Study. *Pain Medicine* 2009;10(3):521-30.
18. Howick J, Steinkopf L, Ulyte A, et al. How empathic is your healthcare practitioner? A systematic review and meta-analysis of patient surveys. *BMC Med Educ* 2017;17(1):136. doi: 10.1186/s12909-017-0967-3 [published Online First: 2017/08/22]
19. Quince TA, Parker RA, Wood DF, et al. Stability of empathy among undergraduate medical students: a longitudinal study at one UK medical school. *BMC Med Educ* 2011;11:90. doi: 10.1186/1472-6920-11-90 [published Online First: 2011/10/27]

For peer review only

For peer review only - http://bmjopen.bmj.com/site/about/guidelines.xhtml

20. Costa-Drolon E, Verneuil L, Manolios E, et al. Medical Students' Perspectives on Empathy: A Systematic Review and Metasynthesis. *Acad Med* 2021;96(1):142-54. doi: 10.1097/acm.0000000000003655 [published Online First: 2020/08/10]
21. Murphy M, Scott LJ, Salisbury C, et al. Implementation of remote consulting in UK primary care following the COVID-19 pandemic: a mixed-methods longitudinal study. *Br J Gen Pract* 2021;71(704):e166. doi: 10.3399/BJGP.2020.0948
22. Howick J, Moscrop A, Mebius A, et al. Effects of empathic and positive communication in healthcare consultations: a systematic review and meta-analysis. *J R Soc Med* 2018;111(7):240-52. doi: 10.1177/0141076818769477 [published Online First: 2018/04/20]
23. Michie S, van Stralen MM, West R. The behaviour change wheel: A new method for characterising and designing behaviour change interventions. *Implementation Science* 2011;6(1):42. doi: 10.1186/1748-5908-6-42
24. Smith KA, Vennik J, Morrison L, et al. Harnessing placebo effects in primary care: Using the person-based approach to develop an online intervention to enhance practitioners' communication of clinical empathy and realistic optimism during consultations. *Frontiers in Pain Research* 2021;2 doi: 10.3389/fpain.2021.721222
25. Little P, Everitt H, Williamson I, et al. Observational study of effect of patient centredness and positive approach on outcomes of general practice consultations. *Br Med J* 2001;323 908-11.
26. Little P, White P, Kelly J, et al. Verbal and non-verbal behaviour and patient perception of communication in primary care: an observational study. *Br J Gen Pract* 2015;65(635):e357-e65. doi: 10.3399/bjgp15X685249
27. Little P, White P, Kelly J, et al. Randomised controlled trial of a brief intervention targeting predominantly non-verbal communication in general practice consultations. *Br J Gen Pract* 2015;65(635):e351-e56. doi: 10.3399/bjgp15X685237
28. Griffin SJ, Kinmonth AL, Veltman MWM, et al. Effect on Health-Related Outcomes of Interventions to Alter the Interaction Between Patients and Practitioners: A Systematic Review of Trials. *Annals of family medicine* 2004;2(6):595-608.
29. Dwamena F, Holmes-Rovner M, Gaulden CM, et al. Interventions for providers to promote a patient-centred approach in clinical consultations. *Cochrane Database of Systematic Reviews* 2012;12:Art. No.: CD003267. DOI: 10.1002/14651858.CD003267.pub2.
30. Howick J, Mittoo S, Abel L, et al. A price tag on clinical empathy? Factors influencing its cost-effectiveness. *J R Soc Med* 2020;113(10):389-93. doi: 10.1177/0141076820945272
31. Kreuter MW, Wray RJ. Tailored and targeted health communication: strategies for enhancing information relevance. *American Journal of Health Behavior* 2003;27(1):S227-S32.
32. Glasgow RE, Vogt TM, Boles SM. Evaluating the public health impact of health promotion interventions: the RE-AIM framework. *Am J Public Health* 1999;89(9):1322-7. doi: 10.2105/ajph.89.9.1322 [published Online First: 1999/09/04]
33. Glasgow RE, Harden SM, Gaglio B, et al. RE-AIM planning and evaluation framework: adapting to new science and practice with a 20-year review. *Frontiers in Public Health* 2019;7:64. doi: 10.3389/fpubh.2019.00064
34. Chan A-W, Tetzlaff JM, Altman DG, et al. SPIRIT 2013 Statement: Defining standard protocol items for clinical trials. *Ann Intern Med* 2013;158(3):200-07. doi: 10.7326/0003-4819-158-3-201302050-00583
35. Primary Care Workforce Team NHS England. General Practice Workforce, 31 December 2023. Official Statistics.: NHS England; 2024 [updated 25 Jan 2024 Available from: https://digital.nhs.uk/data-and-information/publications/statistical/general-and-personal-medical-services/31-december-2023 accessed 09 Feb 2024.
36. Organization WH. International Statistical Classification of Diseases and Related Health Problems (ICD). https://www.who.int/standards/classifications/classification-of-diseases, 2021.
37. Whitaker P. Ticking the ICE box: the future of doctor-patient communication in a post-covid world. *BMJ* 2021;373:n870. doi: 10.1136/bmj.n870

For peer review only - http://bmjopen.bmj.com/site/about/guidelines.xhtml

38. Yardley L, Osmond A, Hare J, et al. Introduction to the LifeGuide: software facilitating the development of interactive behaviour change internet interventions. . In Adaptive and Emergent Behaviour and Complex Systems-Proceedings of the 23rd Convention of the Society for the Study of Artificial Intelligence and Simulation of Behaviour AISB2009.
39. Yardley L, Morrison L, Bradbury K, et al. The person-based approach to intervention development: Application to digital health-related behavior change interventions. *J Med Internet Res* 2015;17(1)
40. Smith KA, Bishop FL, Dambha-Miller H, et al. Improving Empathy in Healthcare Consultations-a Secondary Analysis of Interventions. *J Gen Intern Med* 2020;35(10):3007-14. doi: 10.1007/s11606-020-05994-w [published Online First: 2020/07/14]
41. Howick J, Lyness E, Albury C, et al. Anatomy of positive messages in healthcare consultations: component analysis of messages within 22 randomised trials. *Eur J Pers Cent Healthc* 2019;17:656-64.
42. Budd G, Griffiths D, Howick J, et al. Empathy in patient-clinician interactions when using telecommunication: A rapid review of the evidence. *PEC Innovation* 2022;1:100065. doi: https://doi.org/10.1016/j.pecinn.2022.100065
43. Lyness E, Vennik JL, Bishop FL, et al. Exploring patient views of empathic optimistic communication for osteoarthritis in primary care: a qualitative interview study using vignettes. *BJGP Open* 2021:BJGPO.2021.0014. doi: 10.3399/BJGPO.2021.0014
44. Vennik J, Hughes S, Smith KA, et al. Patient and practitioner priorities and concerns about primary healthcare interactions for osteoarthritis: A meta-ethnography. *Patient Educ Couns* 2022 doi: https://doi.org/10.1016/j.pec.2022.01.009
45. Hughes S, Vennik JL, Smith KA, et al. Clinician views on optimism and empathy in primary care consultations: a qualitative interview study. *BJGP Open* 2022;6(3):BJGPO.2021.0221. doi: 10.3399/BJGPO.2021.0221
46. Vennik J, Hughes S, Lyness E, et al. Patient perceptions of empathy in primary care telephone consultations: A mixed methods study. *Patient Educ Couns* 2023;113:107748. doi: https://doi.org/10.1016/j.pec.2023.107748
47. Dworkin RH, Turk DC, Wyrwich KW, et al. Interpreting the clinical importance of treatment outcomes in chronic pain clinical trials: IMMPACT recommendations. *J Pain* 2008;9(2):105-21. doi: 10.1016/j.jpain.2007.09.005 [published Online First: 2007/12/07]
48. Adams G, Gulliford MC, Ukoumunne OC, et al. Patterns of intra-cluster correlation from primary care research to inform study design and analysis. *J Clin Epidemiol* 2004;57(8):785-94. doi: 10.1016/j.jclinepi.2003.12.013 [published Online First: 2004/10/16]
49. Bishop FL, Smith KA, Vennik J, et al. Feasibility Study of a Novel Online Intervention to Enhance Practitioners' Communication of Clinical Empathy and Realistic Optimism During Primary Care Consultations, 2024, in preparation.
50. Dworkin RH, Turk DC, Farrar JT, et al. Core outcome measures for chronic pain clinical trials: IMMPACT recommendations. *Pain* 2005;113(1-2):9-19.
51. Kroenke K, Krebs EE, Turk D, et al. Core outcome measures for chronic musculoskeletal pain research: recommendations from a veterans health administration work group. *Pain Med* 2019;20(8):1500-08. doi: 10.1093/pm/pny279 [published Online First: 2019/01/08]
52. Smith TO, Hawker GA, Hunter DJ, et al. The OMERACT-OARSI Core domain set for measurement in clinical trials of hip and/or knee osteoarthritis. *The Journal of Rheumatology* 2019:jrheum.181194. doi: 10.3899/jrheum.181194
53. Chiarotto A, Deyo RA, Terwee CB, et al. Core outcome domains for clinical trials in non-specific low back pain. *Eur Spine J* 2015;24(6):1127-42. doi: 10.1007/s00586-015-3892-3
54. Chiarotto A, Boers M, Deyo RA, et al. Core outcome measurement instruments for clinical trials in nonspecific low back pain. *Pain* 2018;159(3)
55. Keller S, Bann CM, Dodd SL, et al. Validity of the Brief Pain Inventory for use in documenting the outcomes of patients with noncancer pain. *Clin J Pain* 2004;20(5):309-18.

For peer review only - http://bmjopen.bmj.com/site/about/guidelines.xhtml
For peer review only

56. Howie JG, Heaney DJ, Maxwell M, et al. A comparison of a Patient Enablement Instrument (PEI) against two established satisfaction scales as an outcome measure of primary care consultations. *Fam Pract* 1998;15(2):165-71.
57. Morrison LG, Geraghty AWA, Lloyd S, et al. Comparing usage of a web and app stress management intervention: An observational study. *Internet Interventions* 2018;12:74-82. doi: https://doi.org/10.1016/j.invent.2018.03.006
58. Molgaard Nielsen A, Hartvigsen J, Kongsted A, et al. The patient enablement instrument for back pain: reliability, content validity, construct validity and responsiveness. *Health and Quality of Life Outcomes* 2021;19(1):116. doi: 10.1186/s12955-021-01758-0 [published Online First: 2021/04/11]
59. Bedford LE, Yeung MHY, Au CH, et al. The validity, reliability, sensitivity and responsiveness of a modified Patient Enablement Instrument (PEI-2) as a tool for serial measurements of health enablement. *Fam Pract* 2020 doi: 10.1093/fampra/cmaa102
60. Preston CC, Colman AM. Optimal number of response categories in rating scales: reliability, validity, discriminating power, and respondent preferences. *Acta Psychol (Amst)* 2000;104(1):1-15. doi: https://doi.org/10.1016/S0001-6918(99)00050-5
61. Fischer D, Stewart AL, Bloch DA, et al. Capturing the Patient's View of Change as a Clinical Outcome Measure. *JAMA* 1999;282(12):1157-62. doi: 10.1001/jama.282.12.1157
62. Wolf MH, Putnam SM, James SA, et al. The Medical Interview Satisfaction Scale: development of a scale to measure patient perceptions of physician behavior. *J Behav Med* 1978;1(4):391-401. [published Online First: 1978/12/01]
63. Meakin R, Weinman J. The 'Medical Interview Satisfaction Scale' (MISS-21) adapted for British general practice. *Fam Pract* 2002;19(3):257-63. doi: 10.1093/fampra/19.3.257
64. Herdman M, Gudex C, Lloyd A, et al. Development and preliminary testing of the new five-level version of EQ-5D (EQ-5D-5L). *Qual Life Res* 2011;20(10):1727-36. doi: 10.1007/s11136-011-9903-x [published Online First: 2011/04/12]
65. National Institute for Health and Care Excellence. Developing NICE guidelines: the manual. London, UK: National Institute for Health and Care Excellence (NICE) 2014.
66. Manca A, Hawkins N, Sculpher MJ. Estimating mean QALYs in trial-based cost-effectiveness analysis: the importance of controlling for baseline utility. *Health Econ* 2005;14(5):487-96. doi: 10.1002/hec.944 [published Online First: 2004/10/22]
67. Al-Janabi H, Flynn TN, Coast J. Development of a self-report measure of capability wellbeing for adults: the ICECAP-A. *Qual Life Res* 2012;21(1):167-76. doi: 10.1007/s11136-011-9927-2 [published Online First: 2011/05/21]
68. Keeley T, Coast J, Nicholls E, et al. An analysis of the complementarity of ICECAP-A and EQ-5D-3 L in an adult population of patients with knee pain. *Health and quality of life outcomes* 2016;14:36. doi: 10.1186/s12955-016-0430-x [published Online First: 2016/03/05]
69. Garfield K, Husbands S, Thorn JC, et al. Development of a brief, generic, modular resource-use measure (ModRUM): cognitive interviews with patients. *BMC Health Serv Res* 2021;21(1):371. doi: 10.1186/s12913-021-06364-w
70. Reilly MC, Zbrozek AS, Dukes EM. The validity and reproducibility of a work productivity and activity impairment instrument. *Pharmacoeconomics* 1993;4(5):353-65. doi: 10.2165/00019053-199304050-00006 [published Online First: 1993/10/05]
71. NHS England and NHS Improvement. National Schedule of NHS Costs, 2020.
72. Joint Formulary Committee. British National Formulary (online). London: BMJ Group and Pharmaceutical Press.
73. Curtis L, Burns A. Unit Costs of Health & Social Care 2020. University of Kent: PSSRU 2020.
74. Office for National Statistics. Annual Survey of Hours and Earnings 2020. UK: Office for National Statistics, 2020.
75. Bandura A. Guide for constructing self-efficacy scales. In: Urdan T, Pajares F, eds. Self-Efficacy Beliefs of Adolescents: Information Age Publishing 2006:307-37.

For peer review only

For peer review only - http://bmjopen.bmj.com/site/about/guidelines.xhtml

76. Ajzen I. Constructing a theory of planned behavior questionnaire. 2019. Available from http://people.umass.edu/aizen/pdf/tpb.measurement.pdf. Accessed 16 February 2024.
77. Renner B, Schwarzer R. Risk and Health Behaviors. Documentation of the Scales of the Research Project: "Risk Appraisal Consequences in Korea" (RACK). http://www.gesundheitsrisiko.de/docs/RACKEnglish.pdf: International University Bremen & Freie Universität Berlin, 2007.

78. Francis JJ, Eccles MP, Johnston M, et al. Constructing questionnaires based on the theory of planned behaviour. A manual for health services researchers. University of Newcastle: Centre for Health Services Research, 2004.
79. Mercer SW, Maxwell M, Heaney D, et al. The development and preliminary validation of the Consultation and Relational Empathy (CARE) measure: an empathy-based consultation process measure. *Fam Pract* 2004;21 699-705.
80. Alberts J, Löwe B, Glahn MA, et al. Development of the generic, multidimensional Treatment Expectation Questionnaire (TEX-Q) through systematic literature review, expert surveys and qualitative interviews. *BMJ Open* 2020;10(8):e036169. doi: 10.1136/bmjopen-2019-036169
81. Bjelland I, Dahl AA, Haug TT, et al. The validity of the Hospital Anxiety and Depression Scale: An updated literature review. *J Psychosom Res* 2002;52(2):69-77.
82. Zigmond AS, Snaith RP. The Hospital Anxiety and Depression Scale. *Acta Psychiatr Scand* 1983;67(6):361-70.
83. Ridd MJ, Lewis G, Peters TJ, et al. Patient-Doctor Depth-of-Relationship Scale: Development and Validation. *The Annals of Family Medicine* 2011;9(6):538. doi: 10.1370/afm.1322
84. Miller S, Ainsworth B, Yardley L, et al. A Framework for Analyzing and Measuring Usage and Engagement Data (AMUsED) in Digital Interventions: Viewpoint. J Med Internet Res 2019;21(2):e10966. doi: 10.2196/10966 [published Online First: 2019/02/16]
85. Campbell MK, Piaggio G, Elbourne DR, et al. Consort 2010 statement: extension to cluster randomised trials. *BMJ* 2012;345:e5661. doi: 10.1136/bmj.e5661 [published Online First: 2012/09/07]
86. Boutron I, Altman DG, Moher D, et al. CONSORT Statement for Randomized Trials of Nonpharmacologic Treatments: A 2017 Update and a CONSORT Extension for Nonpharmacologic Trial Abstracts. *Ann Intern Med* 2017;167(1):40-47. doi: 10.7326/m17-0046 [published Online First: 2017/06/21]
87. Levitt HM, Bamberg M, Creswell JW, et al. Journal article reporting standards for qualitative primary, qualitative meta-analytic, and mixed methods research in psychology: The APA Publications and Communications Board task force report. *Am Psychol* 2018;73(1):26-46. doi: 10.1037/amp0000151 [published Online First: 2018/01/19]

88. May CR, Finch T, Ballini L, et al. Evaluating complex interventions and health technologies using normalization process theory: development of a simplified approach and web-enabled toolkit. BMC Health Serv Res 2011;11(1):245. doi: 10.1186/1472-6963-11-245

For peer review only

For peer review only - http://bmjopen.bmj.com/site/about/guidelines.xhtml

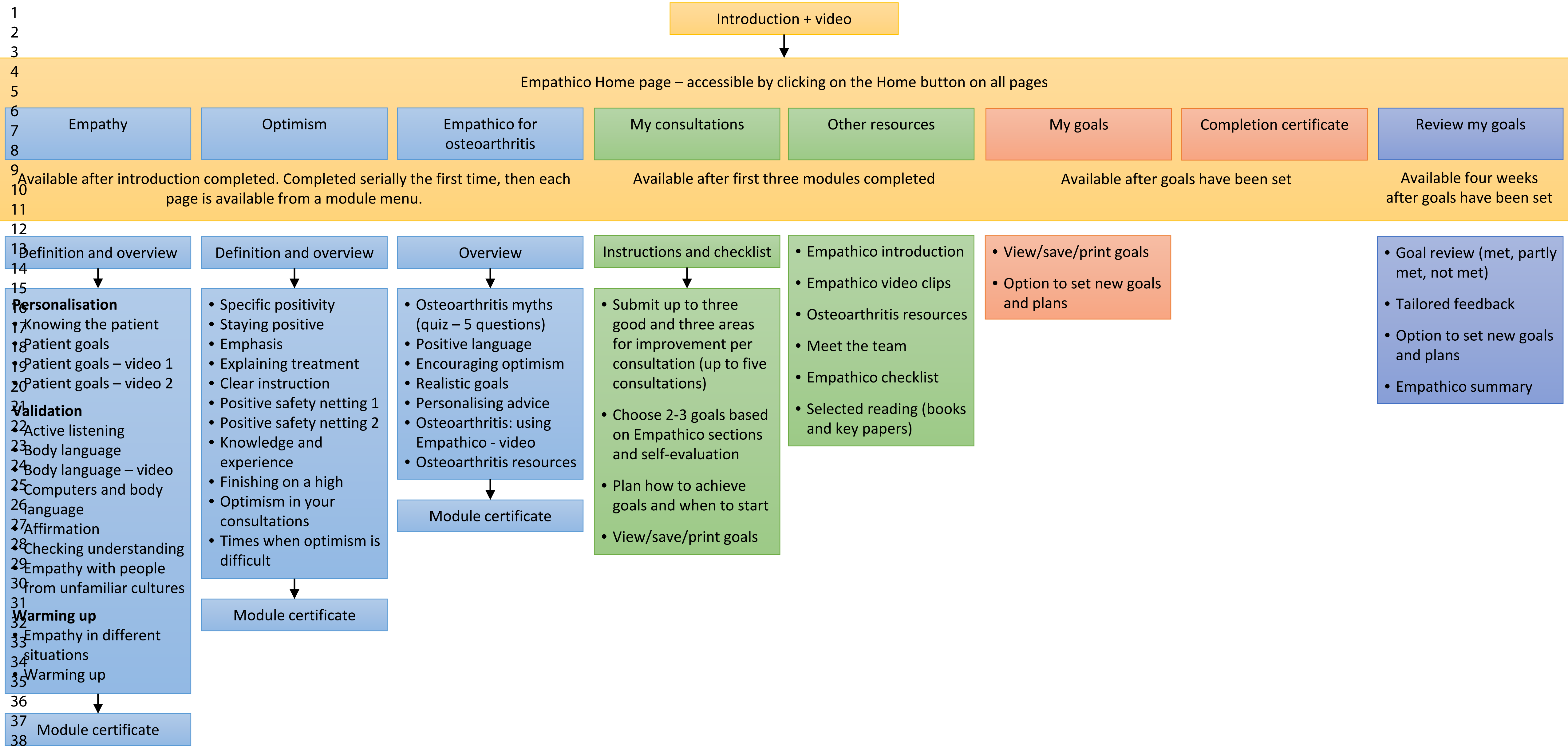
Introduction + video
Empathico Home page – accessible by clicking on the Home button on all pages
Empathy
Optimism
Empathico for osteoarthritis
My consultations
Other resources
My goals
Completion certificate
Review my goals
Available after introduction completed. Completed serially the first time, then each page is available from a module menu.
Available after first three modules completed
Available after goals have been set
Available four weeks after goals have been set
Definition and overview
Personalisation
• Knowing the patient
• Patient goals
• Patient goals – video 1
• Patient goals – video 2
Validation
• Active listening
• Body language
• Body language – video
• Computers and body language
• Affirmation
• Checking understanding
• Empathy with people from unfamiliar cultures
Warming up
• Empathy in different situations
• Warming up
Module certificate
Definition and overview
• Specific positivity
• Staying positive
• Emphasis
• Explaining treatment
• Clear instruction
• Positive safety netting 1
• Positive safety netting 2
• Knowledge and experience
• Finishing on a high
• Optimism in your consultations
• Times when optimism is difficult
Module certificate
Overview
• Osteoarthritis myths (quiz – 5 questions)
• Positive language
• Encouraging optimism
• Realistic goals
• Personalising advice
• Osteoarthritis: using Empathico - video
• Osteoarthritis resources
Module certificate
Instructions and checklist
• Submit up to three good and three areas for improvement per consultation (up to five consultations)
• Choose 2-3 goals based on Empathico sections and self-evaluation
• Plan how to achieve goals and when to start
• View/save/print goals
• Empathico introduction
• Empathico video clips
• Osteoarthritis resources
• Meet the team
• Empathico checklist
• Selected reading (books and key papers)
• View/save/print goals
• Option to set new goals and plans
• Goal review (met, partly met, not met)
• Tailored feedback
• Option to set new goals and plans
• Empathico summary

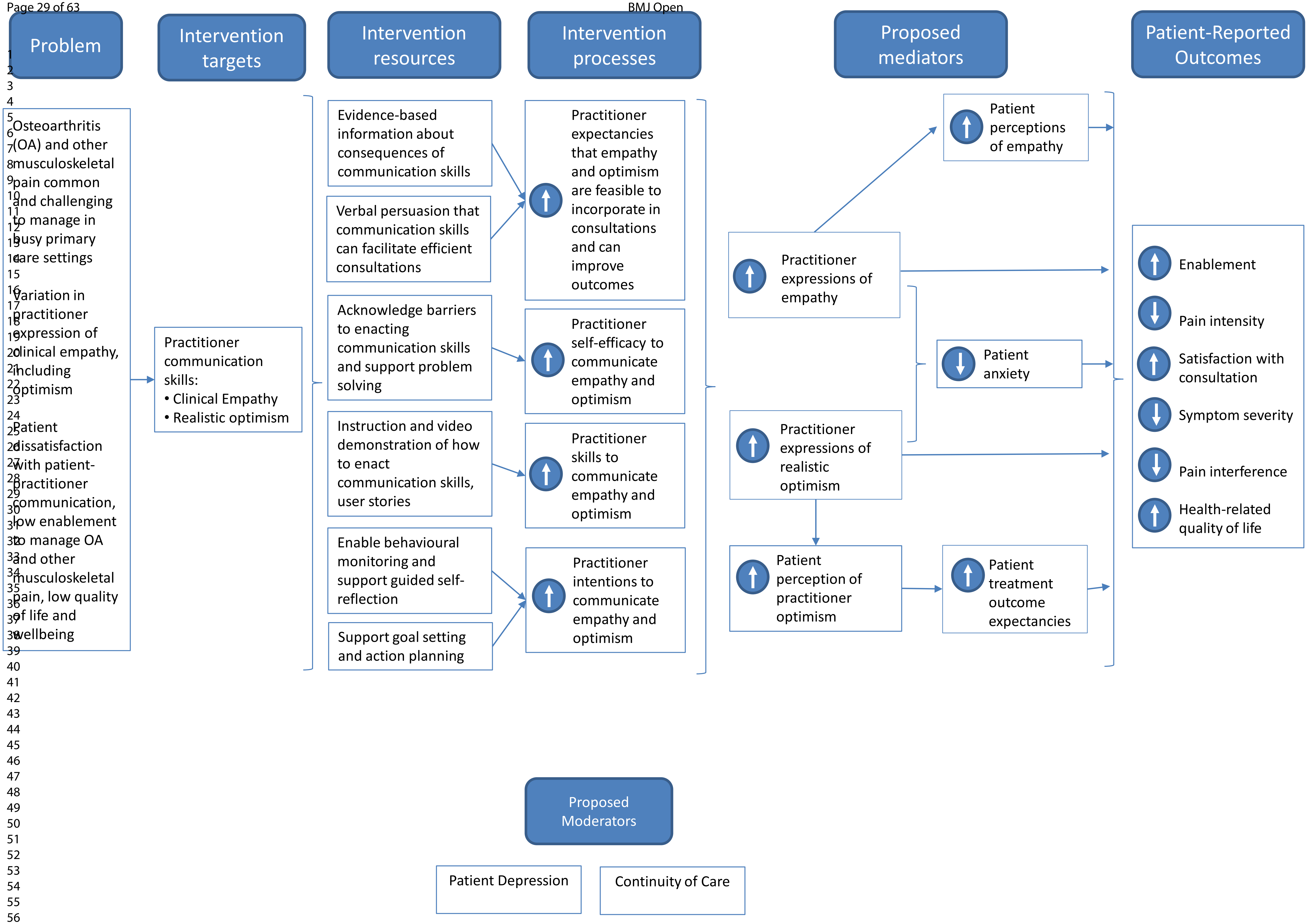
Problem
Intervention targets
Intervention resources
Intervention processes
Proposed mediators
Patient-Reported Outcomes
Osteoarthritis (OA) and other musculoskeletal pain common and challenging to manage in busy primary care settings
Variation in practitioner expression of clinical empathy, including optimism
Patient dissatisfaction with patient-practitioner communication, low enablement to manage OA and other musculoskeletal pain, low quality of life and wellbeing
Practitioner communication skills:
• Clinical Empathy
• Realistic optimism
Evidence-based information about consequences of communication skills
Verbal persuasion that communication skills can facilitate efficient consultations
Acknowledge barriers to enacting communication skills and support problem solving
Instruction and video demonstration of how to enact communication skills, user stories
Enable behavioural monitoring and support guided self-reflection
Support goal setting and action planning
Practitioner expectancies that empathy and optimism are feasible to incorporate in consultations and can improve outcomes
Practitioner self-efficacy to communicate empathy and optimism
Practitioner skills to communicate empathy and optimism
Practitioner intentions to communicate empathy and optimism
Patient perceptions of empathy
Practitioner expressions of empathy
Patient anxiety
Practitioner expressions of realistic optimism
Patient perception of practitioner optimism
Patient treatment outcome expectancies
Enablement
Pain intensity
Satisfaction with consultation
Symptom severity
Pain interference
Health-related quality of life
Proposed Moderators
Patient Depression
Continuity of Care

STANDARD PROTOCOL ITEMS: RECOMMENDATIONS FOR INTERVENTIONAL TRIALS

SPIRIT 2013 Checklist: Recommended items to address in a clinical trial protocol and related documents*

Section/item	Item No	Description	P#
**Administrative information**			
Title	1	Descriptive title identifying the study design, population, interventions, and, if applicable, trial acronym	1
Trial registration	2a	Trial identifier and registry name. If not yet registered, name of intended registry	1
	2b	All items from the World Health Organization Trial Registration Data Set	✓
Protocol version	3	Date and version identifier	All
Funding	4	Sources and types of financial, material, and other support	1
Roles and responsibilities	5a	Names, affiliations, and roles of protocol contributors	1-2
	5b	Name and contact information for the trial sponsor	16
	5c	Role of study sponsor and funders, if any, in study design; collection, management, analysis, and interpretation of data; writing of the report; and the decision to submit the report for publication, including whether they will have ultimate authority over any of these activities	3
	5d	Composition, roles, and responsibilities of the coordinating centre, steering committee, endpoint adjudication committee, data management team, and other individuals or groups overseeing the trial, if applicable (see Item 21a for data monitoring committee)	16
**Introduction**			
Background and rationale	6a	Description of research question and justification for undertaking the trial, including summary of relevant studies (published and unpublished) examining benefits and harms for each intervention	6
	6b	Explanation for choice of comparators	8
Objectives	7	Specific objectives or hypotheses	6-7
Trial design	8	Description of trial design including type of trial (eg, parallel group, crossover, factorial, single group), allocation ratio, and framework (eg, superiority, equivalence, noninferiority, exploratory)	7-8

For peer review only

Methods: Participants, interventions, and outcomes

Study setting	9	Description of study settings (eg, community clinic, academic hospital) and list of countries where data will be collected. Reference to where list of study sites can be obtained	8
Eligibility criteria	10	Inclusion and exclusion criteria for participants. If applicable, eligibility criteria for study centres and individuals who will perform the interventions (eg, surgeons, psychotherapists)	8-9
Interventions	11a	Interventions for each group with sufficient detail to allow replication, including how and when they will be administered	9-10
	11b	Criteria for discontinuing or modifying allocated interventions for a given trial participant (eg, drug dose change in response to harms, participant request, or improving/worsening disease)	14
	11c	Strategies to improve adherence to intervention protocols, and any procedures for monitoring adherence (eg, drug tablet return, laboratory tests)	13
	11d	Relevant concomitant care and interventions that are permitted or prohibited during the trial	10
Outcomes	12	Primary, secondary, and other outcomes, including the specific measurement variable (eg, systolic blood pressure), analysis metric (eg, change from baseline, final value, time to event), method of aggregation (eg, median, proportion), and time point for each outcome. Explanation of the clinical relevance of chosen efficacy and harm outcomes is strongly recommended	11-13
Participant timeline	13	Time schedule of enrolment, interventions (including any run-ins and washouts), assessments, and visits for participants. A schematic diagram is highly recommended (see Figure)	22-23
Sample size	14	Estimated number of participants needed to achieve study objectives and how it was determined, including clinical and statistical assumptions supporting any sample size calculations	10-11
Recruitment	15	Strategies for achieving adequate participant enrolment to reach target sample size	10

Methods: Assignment of interventions (for controlled trials)

Allocation:

Sequence generation	16a	Method of generating the allocation sequence (eg, computer-generated random numbers), and list of any factors for stratification. To reduce predictability of a random sequence, details of any planned restriction (eg, blocking) should be provided in a separate document that is unavailable to those who enrol participants or assign interventions	13-4

Allocation concealment mechanism	16b	Mechanism of implementing the allocation sequence (eg, central telephone; sequentially numbered, opaque, sealed envelopes), describing any steps to conceal the sequence until interventions are assigned	13-4
Implementation	16c	Who will generate the allocation sequence, who will enrol participants, and who will assign participants to interventions	14
Blinding (masking)	17a	Who will be blinded after assignment to interventions (eg, trial participants, care providers, outcome assessors, data analysts), and how	14
	17b	If blinded, circumstances under which unblinding is permissible, and procedure for revealing a participant's allocated intervention during the trial	14
**Methods: Data collection, management, and analysis**			
Data collection methods	18a	Plans for assessment and collection of outcome, baseline, and other trial data, including any related processes to promote data quality (eg, duplicate measurements, training of assessors) and a description of study instruments (eg, questionnaires, laboratory tests) along with their reliability and validity, if known. Reference to where data collection forms can be found, if not in the protocol	11-13
	18b	Plans to promote participant retention and complete follow-up, including list of any outcome data to be collected for participants who discontinue or deviate from intervention protocols	11
Data management	19	Plans for data entry, coding, security, and storage, including any related processes to promote data quality (eg, double data entry; range checks for data values). Reference to where details of data management procedures can be found, if not in the protocol	14
Statistical methods	20a	Statistical methods for analysing primary and secondary outcomes. Reference to where other details of the statistical analysis plan can be found, if not in the protocol	14
	20b	Methods for any additional analyses (eg, subgroup and adjusted analyses)	14
	20c	Definition of analysis population relating to protocol non-adherence (eg, as randomised analysis), and any statistical methods to handle missing data (eg, multiple imputation)	14
**Methods: Monitoring**			
Data monitoring	21a	Composition of data monitoring committee (DMC); summary of its role and reporting structure; statement of whether it is independent from the sponsor and competing interests; and reference to where further details about its charter can be found, if not in the protocol. Alternatively, an explanation of why a DMC is not needed	16

For peer review only - http://bmjopen.bmj.com/site/about/guidelines.xhtml

	21b	Description of any interim analyses and stopping guidelines, including who will have access to these interim results and make the final decision to terminate the trial	15
Harms	22	Plans for collecting, assessing, reporting, and managing solicited and spontaneously reported adverse events and other unintended effects of trial interventions or trial conduct	15
Auditing	23	Frequency and procedures for auditing trial conduct, if any, and whether the process will be independent from investigators and the sponsor	15
**Ethics and dissemination**			
Research ethics approval	24	Plans for seeking research ethics committee/institutional review board (REC/IRB) approval	4
Protocol amendments	25	Plans for communicating important protocol modifications (eg, changes to eligibility criteria, outcomes, analyses) to relevant parties (eg, investigators, REC/IRBs, trial participants, trial registries, journals, regulators)	16
Consent or assent	26a	Who will obtain informed consent or assent from potential trial participants or authorised surrogates, and how (see Item 32)	10
	26b	Additional consent provisions for collection and use of participant data and biological specimens in ancillary studies, if applicable	N/A
Confidentiality	27	How personal information about potential and enrolled participants will be collected, shared, and maintained in order to protect confidentiality before, during, and after the trial	14
Declaration of interests	28	Financial and other competing interests for principal investigators for the overall trial and each study site	3
Access to data	29	Statement of who will have access to the final trial dataset, and disclosure of contractual agreements that limit such access for investigators	16
Ancillary and post-trial care	30	Provisions, if any, for ancillary and post-trial care, and for compensation to those who suffer harm from trial participation	15
Dissemination policy	31a	Plans for investigators and sponsor to communicate trial results to participants, healthcare professionals, the public, and other relevant groups (eg, via publication, reporting in results databases, or other data sharing arrangements), including any publication restrictions	16
	31b	Authorship eligibility guidelines and any intended use of professional writers	16
	31c	Plans, if any, for granting public access to the full protocol, participant-level dataset, and statistical code	16

For peer review only - http://bmjopen.bmj.com/site/about/guidelines.xhtml

Appendices

Informed consent materials	32	Model consent form and other related documentation given to participants and authorised surrogates	10
Biological specimens	33	Plans for collection, laboratory evaluation, and storage of biological specimens for genetic or molecular analysis in the current trial and for future use in ancillary studies, if applicable	N/A

*It is strongly recommended that this checklist be read in conjunction with the SPIRIT 2013 Explanation & Elaboration for important clarification on the items. Amendments to the protocol should be tracked and dated. The SPIRIT checklist is copyrighted by the SPIRIT Group under the Creative Commons "Attribution-NonCommercial-NoDerivs 3.0 Unported" license.

For peer review only

Online Supplementary File 2: PIS and Consent Forms

Contents

Participant Information Sheet for Patients 2

Patient Consent Form 6

Participant Information Sheet for Practitioners 7

Practitioner Consent Form 12

For peer review only

Participant Information Sheet for Patients

Version 2. Date 22.6.22.

Participant Information Sheet

Chief Investigators: Dr Felicity Bishop and Professor Hazel Everitt, University of Southampton

We invite you to take part in the TIP study

Please read on to find out why we are doing this study and what it involves. To help you decide whether to take part you may like to talk to others. If you want to talk to us, the researchers, or ask us some questions, please email <insert local researcher email address> or phone < local researcher number>. If you want to take part, you can tell us by answering the questions at the bottom of this page.

What if I need some help to take part?

We want lots of different people to take part in this study. And we know that different people will need different kinds of help.

We can help with things like understanding the documents, or if you have problems using the internet or if you would prefer paper copies of things posted to you.

If you need an interpreter, you are welcome to ask a family member or friend to help you with this study. Or you can ask us and we will do our best to get an interpreter for you.

If you would like some help to understand or take part in this study, please get in touch with us. You can contact us by phone < local researcher number >or email <<insert local researcher email address>>

A quick summary of the study

- This study will help us understand patients' experiences of appointments with GPs, Nurses, or Physiotherapists. We'll call these people "Primary Care Practitioners".
- We want to know what you think about how your Primary Care Practitioner talks to you during consultations.
- The study is being run by The Universities of Southampton, Bristol, Warwick, Oxford, and Keele University. It is funded by the National School for Primary Care Research (SPCR).
- The South Central-Hampshire B Research Ethics Committee has given a favourable opinion of the study. This means that a group of independent people have looked at our research and feel that it is ethically acceptable.

Why have I been asked to participate?

Because you are an adult and have an appointment with a Primary Care Practitioner who is already taking part in the TIP study

For peer review only - http://bmjopen.bmj.com/site/about/guidelines.xhtml

What will happen if I take part?

We will ask you to read some documents (like this one) and fill in some questionnaires.

Before your appointment

Read this information.

Answer a few questions to check you can take part in the study.

Answer a few questions to tell us if you want to take part in the study.

During your appointment

Your consultation at your GP surgery will happen as normal.

Please do not discuss this study with your Primary Care Practitioner.

It is important that your Primary Care Practitioner does not know whether you are taking part.

Within 1 week of your appointment

You will be sent a link to fill in some questionnaires. These questionnaires ask about your appointment, your health, and your quality of life. They take about 15 minutes to do.

1 month, 3 months, and 6 months after your first appointment

You will be sent a link to fill in some questionnaires. These questionnaires ask about your health and quality of life. They take about 15 minutes to do.

We might also invite you to take part in two meetings (interviews) with a researcher. If you are asked to do an interview, this would be in the first week after your appointment and again in 6 months' time. In the interview, the researcher will ask about your experiences of primary care appointments and your experiences of doing this study..

What are the possible pros and cons of taking part?

Taking part will help us understand the best ways for primary care practitioners to talk to patients during consultations. We do not think that taking part in this study poses any risks for you To thank you for taking part we will give you two £10 vouchers. We will send the first voucher after you complete the 1 month questionnaire. We will send the second voucher after you complete the 6 month questionnaire. If you take part in an interview as well as doing the questionnaires, then we will send you an extra £10 voucher for each interview.

For peer review only - http://bmjopen.bmj.com/site/about/guidelines.xhtml

Do I have to take part?

No, it is up to you to decide .

If you decide to take part now, you can still change your mind later. You can pull out from the study at any time by contacting the researcher by email or phone. You won't have to give a reason. Your routine health care won't be affected at all. If you pull out of the study, we will keep the information that you've already given us.

What information will be collected?

You will probably fill in our questionnaires on the internet. Although, if you would rather have a paper questionnaire please ask us and we can give you one.

Our questionnaires are on a secure service called Qualtrics. Qualtrics meets the highest standards for privacy and data security. We will download all the completed questionnaires. We will store this data on a University of Southampton computer server behind the University of Southampton firewall. At the end of the study, we will destroy our records of your personal contact details.

Your name will not appear on any questionnaires you fill in. Your questionnaire answers will be combined with other patients' answers and put in a secure data archive. Only suitably qualified researchers are allowed to ask for access this archive.

One of our questions asks if it's OK to use your questionnaire answers to help other ethically approved research and education activities in the future. If you say "no" you can still take part in the study. Personal data will be collected and stored on a secure server at University of Southampton in compliance with the requirements of the General Data Protection Regulations and the Data Protection Act 2018. We will securely store your name, contact details, and any other personal data you have given us in a separate list, so we know who has taken part. We will only use your contact details to contact you about this study. You do not need to but if you would like to read the full Data Protection Privacy Notice, click here.

Will my participation be confidential?

Your participation and the information we collect about you during the course of the research will be kept strictly confidential.

Nothing you say on the questionnaires or in the interviews will be shared with your Primary Care Practitioner or anyone else in the medical practice.

But, if you say something in an interview which makes the interviewer worried that you might be being abused or neglected then they will raise this with the appropriate people.

The research team may have to give certain other people access to your data. The only other people who might be given access to your data are responsible members of the University of Southampton and regulatory authorities (for example, the Health Research Authority). They need access to make sure the research is being done correctly and in line with regulations. All of these people must keep your information, strictly confidential.

For peer review only - http://bmjopen.bmj.com/site/about/guidelines.xhtml

What will happen to the results of the research?

We hope to publish our results in scientific journals, blogs and conferences. We plan to share our findings with a wide audience including health care professionals, scientists, patients and members of the public. If you would like, we can also send you a summary what we found out. You can ask for this summary when you fill in the questionnaires.

What happens if there is a problem?

If you have a concern about any aspect of this study, you should speak to Nadia Cross who will do her best to answer your questions.

If you remain unhappy or have a complaint about any aspect of this study, please contact the University of Southampton Research Integrity and Governance Manager (023 8059 5058, rgoinfo@soton.ac.uk).

You may also contact your local Patient Advice and Liaison Service (PALS). PALS has been introduced to ensure that the NHS listens to patients, their relatives, carers, and friends, and answers their questions and resolves their concerns as quickly as possible. Your local PALS service can be found at <<INSERT LOCAL DETAILS>>

Where can I get more information?

PLEASE DO NOT DISCUSS YOUR PARTICIPATION IN THE STUDY WITH YOUR GP, NURSE, PHYSIOTHERAPIST, OR ANY OTHER PRIMARY CARE PRACTITIONER.

If you have any questions about the study, you can contact the researcher, <<INSERT NAME>>

Email: <<INSERT>>; or Telephone: TBC>>

You can also contact the study manager, Nadia Cross at tip@soton.ac.uk

Thank you for reading this information and considering taking part in our study.

For peer review only

Patient Consent Form

Version 2. Date 22.6.22.

Patient Consent Form

Chief Investigators: Dr Felicity Bishop and Professor Hazel Everitt, University of Southampton

**Please indicate if you agree with the statement.**	**Yes/No**
I have read and understood the information sheet *(insert date /version no. of participant information sheet)* and have had the opportunity to ask questions about the study.	Yes/No
I agree to take part in this research project and agree for my data to be used for the purpose of this study.	Yes/No
I understand my participation is voluntary and I may withdraw at any time without giving a reason and without my routine health care being affected.	Yes/No
I understand that personal details I provide will be held securely at The University of Southampton in line with General Data Protection Regulation and Data Protection Act 2018.	Yes/No
I agree to take part in this study	Yes/No
Optional: You do not have to agree to this to take part in this research<br><br>I agree that the information collected about me may be used to support other ethically approved research and education activities in the future, and may be stored in a secure data archive and shared anonymously with other suitably-qualified researchers.	Yes/No

For peer review only

For peer review only - http://bmjopen.bmj.com/site/about/guidelines.xhtml

Participant Information Sheet for Practitioners

Version 1. Date 23.3.22.

Practitioner Information Sheet (main study)

Chief Investigators: Dr Felicity Bishop and Professor Hazel Everitt, University of Southampton

We invite you to take part in a research study

It is up to you to decide if you want to take part or not. This leaflet tells you why the study is being done and what it will involve. Please discuss this information with others if you wish. Please contact the research team if anything is unclear or you would like to ask any questions.

A quick summary of the study

- In this cluster randomised trial, your practice will be randomised into one of two groups: intervention arm or control arm.
- Practitioners working in intervention practices will complete communication skills e-learning training and implement the skills in subsequent consultations. Practitioners working in control practices will continue consulting as usual.
- Patients will be recruited at the intervention and control practices, and complete pre-consultation and post-consultation questionnaires.
- Practitioners in both arms will be asked to complete online questionnaires (about communication within consultations) at 3 time points: baseline, 8 weeks, and 34 weeks post-randomisation. Practitioners in the control group will have access to the communication skills e-learning training at the end of the study.
- The study is being run by the Universities of Southampton, Bristol, Keele, Oxford and Warwick, and is funded by the National School for Primary Care Research (SPCR).

What is the research about?

We have developed communication skills e-learning training for GPs, physiotherapists, and nurses to help enhance consultations with osteoarthritis patients. It is also likely that this training will be relevant to other conditions. The TIP (Talking in Primary Care) study aims to test the effectiveness and cost-effectiveness of communication skills e-learning training for primary care practitioners on patients' musculoskeletal pain and enablement.

For peer review only - http://bmjopen.bmj.com/site/about/guidelines.xhtml

Why have I been asked to participate?

You have been asked to take part because you are a GP, physiotherapist or nurse working in primary care, and have experience of treating patients with osteoarthritis. We hope to recruit a range of practitioners with different levels of experience and background.

Do I have to take part?

No, it is entirely up to you to decide whether or not to take part. If you decide that you would like to take part, we will ask you to complete an online consent form.

What will happen to me if I take part?

If you are interested in taking part:

- You will be provided with a link to a study website, provide online consent and complete an online questionnaire (approx. 10 minutes).
- Your practice will be randomised to one of two groups: an intervention arm and a control arm.
- In weeks 1-2, if you are in the intervention arm you will be asked to complete the training. This will take approximately 1-2 hours and can be done in short chunks. If you are in the control arm, you should continue to treat patients as usual and not undertake any training in communication skills.
- In weeks 3-8, we will be recruiting patients from your practice to take part in this study. You may be asked to help with this.
- You will be asked to complete a short online questionnaire about communication within consultations at 3 time points: baseline, 8 weeks, and 34 weeks post-randomisation.
- You will also be offered the opportunity to take part in a research interview to share your experiences of communication within consultations and the TIP study.
- If you are in the control arm, you will be offered access to the e-learning training at the end of the study.

What are the possible pros and cons of taking part?

Participating in the TIP study will give you the opportunity to learn and implement evidence-based communication skills within your consultations. This could improve patient outcomes and patient satisfaction with care and make best use of primary care appointments. There are no expected risks or disadvantages associated with taking part in this study.

GP practices will be paid service support costs/ excess treatment costs via their CRN for taking part in the TIP study. We will also provide research costs to reimburse practitioners for their time spent taking part in the study.

What happens to the data collected?

For peer review only - http://bmjopen.bmj.com/site/about/guidelines.xhtml

- Electronic questionnaires will be collected using a secure online data collection service which meets the highest industry standards for privacy and data security (Qualtrics).
- Data on patterns and amount of usage of the e-learning training will be collected by the LifeGuide platform on which the e-learning training is hosted.
- All data from Qualtrics and LifeGuide will be downloaded to University of Southampton servers, password-protected and stored securely behind the University of Southampton firewall.
- At the end of the study anonymous questionnaire data will be deposited in a secure data archive which will be made available on request to suitably qualified researchers for further data analysis on this topic.

We will securely store your name and contact details separately from your questionnaire data and will only use these details to contact you about this study. We will permanently delete this at the end of the project.

Will my participation be confidential?

Your participation and the information we collect about you during the course of the research will be kept strictly confidential.

Only members of the research team and responsible members of the University of Southampton may be given access to data about you for monitoring purposes and/or to carry out an audit of the study to ensure that the research is complying with applicable regulations. Individuals from regulatory authorities (people who check that we are carrying out the study correctly) may require access to your data. All of these people have a duty to keep your information, as a research participant, strictly confidential.

What happens if I change my mind?

You have the right to change your mind and withdraw at any time without giving a reason. If you wish to withdraw from the study, please contact Nadia Cross, Trial Manager (details below).

What will happen to the results of the research?

We hope to publish our results in scientific journals and other formats such as blogs and conferences. We plan to share our findings with a wide audience including health care professionals, scientists, patients, and members of the public. If you would like, we will also send you a summary of our findings.

Who is conducting the study?

Our research team includes GPs, health psychologists, academic researchers and patient representatives from the Universities of Southampton, Bristol, Keele, Oxford and Warwick. The research is funded by National School for Primary Care Research (SPCR) and has been approved by the Health Research Authority and the National Research Ethics Committee (reference number: <<xxxxxxxxx>>). The research is being sponsored by University of Southampton.

For peer review only - http://bmjopen.bmj.com/site/about/guidelines.xhtml

What happens if there is a problem?

If you have a concern about any aspect of this study, you should speak to the researchers (contact details above) who will do their best to answer your questions.

If you remain unhappy or have a complaint about any aspect of this study, please contact the University of Southampton Research Integrity and Governance Manager (023 8059 5058, rgoinfo@soton.ac.uk).

Where can I get more information?

If you have any questions, please do not hesitate to get in touch with Nadia Cross, Trial Manager using the contact details below:

**Name**<br>**Role:**<br>**Address:**<br><br><br>**Contact:**	**Nadia Cross**<br>Trial Manager<br>University of Southampton<br>Aldermoor Health Centre<br>Southampton, SO16 5ST<br>*[insert study team contact details@soton.ac.uk]*	

Thank you for taking the time to read the information sheet and considering taking part in the research

** Click out page in Qualtrics*

Data Protection Privacy Notice

The University of Southampton conducts research to the highest standards of research integrity. As a publicly-funded organisation, the University has to ensure that it is in the public interest when we use personally-identifiable information about people who have agreed to take part in research. This means that when you agree to take part in a research study, we will use information about you in the ways needed, and for the purposes specified, to conduct and complete the research project. Under data protection law, 'Personal data' means any information that relates to and is capable of identifying a living individual. The University's data protection policy governing the use of personal data by the University can be found on its website (https://www.southampton.ac.uk/legalservices/what-we-do/data-protection-and-foi.page).

This Participant Information Sheet tells you what data will be collected for this project and whether this includes any personal data. Please ask the research team if you have any questions or are unclear what data is being collected about you.

For peer review only - http://bmjopen.bmj.com/site/about/guidelines.xhtml

Our privacy notice for research participants provides more information on how the University of Southampton collects and uses your personal data when you take part in one of our research projects and can be found at http://www.southampton.ac.uk/assets/sharepoint/intranet/ls/Public/Research%20and%20Integrity%20Privacy%20Notice/Privacy%20Notice%20for%20Research%20Participants.pdf

Any personal data we collect in this study will be used only for the purposes of carrying out our research and will be handled according to the University's policies in line with data protection law. If any personal data is used from which you can be identified directly, it will not be disclosed to anyone else without your consent unless the University of Southampton is required by law to disclose it.

Data protection law requires us to have a valid legal reason ('lawful basis') to process and use your Personal data. The lawful basis for processing personal information in this research study is for the performance of a task carried out in the public interest. Personal data collected for research will not be used for any other purpose.

For the purposes of data protection law, the University of Southampton is the 'Data Controller' for this study, which means that we are responsible for looking after your information and using it properly. The University of Southampton will keep identifiable information about you for 10 years after the study has finished after which time any link between you and your information will be removed.

To safeguard your rights, we will use the minimum personal data necessary to achieve our research study objectives. Your data protection rights – such as to access, change, or transfer such information - may be limited, however, in order for the research output to be reliable and accurate. The University will not do anything with your personal data that you would not reasonably expect.

If you have any questions about how your personal data is used, or wish to exercise any of your rights, please consult the University's data protection webpage (https://www.southampton.ac.uk/legalservices/what-we-do/data-protection-and-foi.page) where you can make a request using our online form. If you need further assistance, please contact the University's Data Protection Officer (data.protection@soton.ac.uk).

For peer review only - http://bmjopen.bmj.com/site/about/guidelines.xhtml

For peer review only

Practitioner Consent Form

Version 2. Date 22.6.22.

Practitioner consent form (main study)

Chief Investigators: Dr Felicity Bishop and Professor Hazel Everitt, University of Southampton

ERGO number: 70489

For peer review only

Please indicate if you agree with the statements:

1. I have read and understood the practitioner information sheet (<<insert version and date>> and have had the opportunity to ask questions about the study.	Yes/No
2. I agree to take part in this research project and agree for my data to be used for the purpose of this study.	Yes/No
3. I understand my participation is voluntary and I may withdraw at any time for any reason.	Yes/No
4. I understand that personal details I provide will be held securely at The University of Southampton in line with General Data Protection Regulation and Data Protection Act 2018.	Yes/No
5. I agree to take part in the TIP study.	Yes/No
Optional: You do not have to agree to this item to take part in this research <br>6. I agree that my questionnaire data may be used to support other ethically approved research and education activities in the future and may be stored in a secure data archive and shared anonymously with other suitably qualified researchers.	Yes/No

Name of participant ……………………………………………………………………………

Date……………………………………

For peer review only - http://bmjopen.bmj.com/site/about/guidelines.xhtml

Online Supplementary File 3: Measures and Timings

Contents

Table 1. Patient-Reported Characteristics, Primary and Secondary Outcomes and Process Variables 2

Table 2. Practitioner-Reported Characteristics, Outcomes and Process Variables 3

References 4

For peer review only

For peer review only - http://bmjopen.bmj.com/site/about/guidelines.xhtml

Table 1. Patient-Reported Characteristics, Primary and Secondary Outcomes and Process Variables

Variable	Measure	Items	Measurement Timings				
			<-7d	<7 d	+1 m	+3 m	+6 m
Primary Outcomes							
Pain intensity (pain sample)	Pain intensity subscale from the BPI[1]	4	x	x	x	x	x
Patient enablement	Modified PEI[2]	6		x	x	x	x
Secondary Outcomes							
Patient global impression of symptom severity	Single item[3]	1	x	x	x	x	x
Patient global impression of symptom change	Single item[3]	1		x	x	x	x
Pain interference	Pain interference subscale from the BPI[1]	7			x		X
Patient satisfaction	MISS for UK general practice[4]	21		x			
Adverse events	Bespoke self-report item	1			x	x	x
Health Economics							
Health-related quality of life	EQ-5D-5L and EQ-VAS[5]	6	x		x		x
Capability wellbeing	ICECAP-A[6,7]	5	x		x		x
Healthcare utilization	ModRUM core module[8]	12		x		x	x
Prescribed medications	ModRUM depth questions[8]	1				x	x
Personal expenses	Bespoke self-report item	3				x	x
Productivity	WPAI:GH	6				x	x
Process Measures							
Perceptions of practitioner empathy	CARE[9]	10		X			
Perceptions of practitioner optimism	Bespoke item	1		X			
Treatment expectations	Treatment expectation questionnaire TEX-Q[10]	15		X			
Anxiety	HADS[11,12]	7		X			
Continuity of care	Patient-Doctor Depth of Relationship Scale[13]	9		X			
Depression	HADS[11,12]	7		X			
Sociodemographic Characteristics							
Age, gender, ethnicity		3	x				
Index of Multiple Deprivation	Postcode	1	x				
Health Characteristics							
Reasons for consulting		1		x			
Comorbidities		1		x			
Index consultation modality		1		x			

For peer review only - http://bmjopen.bmj.com/site/about/guidelines.xhtml

Table 2. Practitioner-Reported Characteristics, Outcomes and Process Variables

Practitioners	Variable	Measure	Items	Measurement Timings			
				Baseline	+2wk	+8wk	+34wk
All	Characteristics (age, gender, ethnicity, years qualified, profession)	Bespoke	5	x			
All	Practitioner self-efficacy for conveying clinical empathy	Bespoke, from feasibility study	7	X		X	x
All	Practitioner self-efficacy for conveying realistic optimism	Bespoke, from feasibility study	5	x		X	x
Intervention arm only	Practitioner outcome expectancy for implementing goals set during EMPathicO training	Bespoke, from feasibility study	16	X		X	x
Intervention arm only	Practitioner intentions to implement goals set during EMPathicO training	Bespoke, from feasibility study	3	X		X	x
Intervention arm only	Practitioner intervention usage	LifeGuide data	N/A			X	X
All	Practitioner-reported other training	Bespoke	1			x	x

For peer review only - http://bmjopen.bmj.com/site/about/guidelines.xhtml

References

1. Keller S, Bann CM, Dodd SL, et al. Validity of the Brief Pain Inventory for use in documenting the outcomes of patients with noncancer pain. *Clin J Pain* 2004;20(5):309-18.
2. Howie JG, Heaney DJ, Maxwell M, et al. A comparison of a Patient Enablement Instrument (PEI) against two established satisfaction scales as an outcome measure of primary care consultations. *Fam Pract* 1998;15(2):165-71.
3. Fischer D, Stewart AL, Bloch DA, et al. Capturing the Patient's View of Change as a Clinical Outcome Measure. *JAMA* 1999;282(12):1157-62. doi: 10.1001/jama.282.12.1157
4. Meakin R, Weinman J. The 'Medical Interview Satisfaction Scale' (MISS-21) adapted for British general practice. *Fam Pract* 2002;19(3):257-63. doi: 10.1093/fampra/19.3.257
5. Herdman M, Gudex C, Lloyd A, et al. Development and preliminary testing of the new five-level version of EQ-5D (EQ-5D-5L). *Qual Life Res* 2011;20(10):1727-36. doi: 10.1007/s11136-011-9903-x [published Online First: 2011/04/12]
6. Al-Janabi H, Flynn TN, Coast J. Development of a self-report measure of capability wellbeing for adults: the ICECAP-A. *Qual Life Res* 2012;21(1):167-76. doi: 10.1007/s11136-011-9927-2 [published Online First: 2011/05/21]
7. Keeley T, Coast J, Nicholls E, et al. An analysis of the complementarity of ICECAP-A and EQ-5D-3 L in an adult population of patients with knee pain. *Health and quality of life outcomes* 2016;14:36. doi: 10.1186/s12955-016-0430-x [published Online First: 2016/03/05]
8. Garfield K, Husbands S, Thorn JC, et al. Development of a brief, generic, modular resource-use measure (ModRUM): cognitive interviews with patients. *BMC Health Serv Res* 2021;21(1):371. doi: 10.1186/s12913-021-06364-w
9. Mercer SW, Maxwell M, Heaney D, et al. The development and preliminary validation of the Consultation and Relational Empathy (CARE) measure: an empathy-based consultation process measure. *Fam Pract* 2004;21 699-705.
10. Alberts J, Löwe B, Glahn MA, et al. Development of the generic, multidimensional Treatment Expectation Questionnaire (TEX-Q) through systematic literature review, expert surveys and qualitative interviews. *BMJ Open* 2020;10(8):e036169. doi: 10.1136/bmjopen-2019-036169
11. Bjelland I, Dahl AA, Haug TT, et al. The validity of the Hospital Anxiety and Depression Scale: An updated literature review. *J Psychosom Res* 2002;52(2):69-77.
12. Zigmond AS, Snaith RP. The Hospital Anxiety and Depression Scale. *Acta Psychiatr Scand* 1983;67(6):361-70.
13. Ridd MJ, Lewis G, Peters TJ, et al. Patient-Doctor Depth-of-Relationship Scale: Development and Validation. *The Annals of Family Medicine* 2011;9(6):538. doi: 10.1370/afm.1322

For peer review only

Talking in Primary Care: A cluster-randomized controlled trial in primary care to test the effectiveness and cost-effectiveness of communication skills e-learning for practitioners on patients' musculoskeletal pain and enablement

Trial Steering Committee Charter

Version 1 22 April 2022

Authorised by:

Name:	Professor Joanne Reeve	Role:	Chairperson
Signature:	Joanne Reeve	Date:	22 April 2022

Prepared by

Name:	Nadia Cross	Role:	Trial Manager
Signature:	Nadia Cross	Date:	22 April 2022

**CONTENT**	**DETAILS OF TSC**
**1. Introduction**	
Name (& Sponsor's ID) of trial	Talking in Primary Care: A cluster-randomized controlled trial in primary care to test the effectiveness and cost-effectiveness of communication skills e-learning for practitioners on patients' musculoskeletal pain and enablement.<br>**UoS ERGO:** 70489<br>**IRAS:** 312208
Objectives of trial, including interventions being investigated	The primary aim is to determine the clinical and cost-effectiveness of EMPathicO training in Clinical Empathy and conveying realistic Positive Messages for practitioners in patients presenting with MSK pain.<br>The secondary aim is to maximize EMPathicO's potential for wide-spread adoption, implementation, and maintenance of effects. We will do this by assessing effects of EMPathicO training on patients presenting with any symptoms other than MSK pain since the impact of EMPathicO will potentially be in all consultations not just MSK consultations; testing how and in what circumstances EMPathicO changes practitioner communication behaviours and patient outcomes for in-person, telephone, and video consultations; and analysing a diverse range of patients' and practitioners' experiences of adoption and longer-term implementation.
Outline of scope of Charter	The purpose of this document is to describe the membership, terms of reference, roles, responsibilities, authority, decision-making and relationships of the Trial Steering Committee (TSC) and the Data Monitoring Committee (DMC) for this trial, including the timing of meetings, methods of providing information to and from the TSC, frequency and format of meetings and relationships with other trial committees.
Facilitation	A member of the TIP team will be nominated as a Facilitator for the trial. The Facilitator will be responsible for the organisation of meetings and should be copied into all communications with and between the TSC.
**2. Roles and responsibilities**	
A broad statement of the aims of the TSC	TSC - To act as the oversight body for the TIP study on behalf of the Sponsor/Funder.<br>DMC - To monitor and review on a 6 monthly basis the main outcomes measures overall conduct in order to safeguard the interests of patients
Terms of reference	The role of the TSC is to provide oversight for the TIP study. It should also provide advice through its independent Chairperson to the Trial Management Group (TMG) and the funder (NIHR-SPCR) on all aspects of the trial.<br>The TSC will also assume responsibilities of the Data Monitoring Committee (DMC) and review information on the progress and accruing data of this trial and provide advice on the conduct of the trial.

CONTENT	DETAILS OF TSC
Specific roles of TSC	• provide expert oversight of the trial<br>• maintain confidentiality of all trial information that is not already in the public domain<br>• make decisions as to the future continuation (or otherwise) of the trial/s<br>• monitor recruitment rates and encourage the TMG to develop strategies to deal with any recruitment problems<br>• comment on the protocol<br>• assess the impact and relevance of any accumulating external evidence<br>• review completion of CRFs and comment on strategies from TMG to encourage satisfactory completion in the future<br>• monitor follow-up rates and review strategies from TMG to deal with problems<br>• censure sites that are deviating from the protocol<br>• comment on any amendments to the protocol, where appropriate<br>• approve any proposals by the TMG concerning any change to the design of the trial, including additional sub-studies<br>• oversee the timely reporting of trial results<br>• comment on the statistical analysis plan<br>• comment on the publication policy<br>• comment on the main trial manuscript<br>• comment on any abstracts and presentations of any results during the running of the trial
Specific roles of DMC delegated to the TSC	Interim review of the trial's progress including updated figures on recruitment, data quality, adherence to protocol treatment and follow-up, and main outcomes and safety data. Specifically, these roles include to:<br>• monitor evidence for treatment harm (e.g. SAEs and deaths)<br>• assess the impact and relevance of external evidence<br>• decide whether to recommend that the trial continues to recruit participants or whether recruitment should be terminated either for everyone or for some treatment groups and/or some participant subgroups<br>• decide whether trial follow-up should be stopped earlier<br>• assess data quality, including completeness (and by so doing encourage collection of high quality data)<br>• maintain confidentiality of all trial information that is not in the public domain<br>• monitor recruitment figures and losses to follow-up<br>• monitor compliance with the protocol by participants and investigators<br>• monitor planned sample size assumptions.<br>• suggest additional data analyses if necessary<br>• advise on protocol modifications proposed by investigators or sponsors (e.g. to inclusion criteria, trial endpoints, or sample size)<br>• monitor continuing appropriateness of patient information

CONTENT	DETAILS OF TSC
**3. Before or early in the trial**	
Whether the TSC will have input into the protocol	All potential TSC members should have sight of the protocol as early as possible. Before recruitment begins the trial will have undergone review by the Sponsor/Funder (e.g. peer review for public sector trials), scrutiny by other trial committees and a research ethics committee. Therefore, if a potential TSC member has major reservations about the trial (e.g. the protocol, the logistics, ethical concerns) they should report these to TMG. TSC members should be constructively critical of the ongoing trial, but also supportive of aims and methods of the trial.
Whether members of the TSC will have a contract	TSC members will not be asked to formally sign a contract but should formally register their agreement to join the group by confirming (1) that they agree to be a member and (2) that they agree with the contents of this Charter. Any potential competing interests should be declared at the same time. Members should complete and return the form in Annexes 1 or 2. Any observers (attendees who are not members) will sign a confidentiality agreement on the first occasion they attend a meeting (Annexe 3).
**4. Composition**	
Membership and size of the TSC	The majority of members of the TSC, including the Chair, should be independent [1]of the trial (see section 5). Non-independent members will also be part of the TSC.<br>The members of the TSC for this trial are:<br>Professor Joanne Reeve (chair) – Independent member<br>Dr Philip Pallmann – Independent member<br>Dr Ines Rombach – Independent member<br>Mr Ian Dickerson – PPI contributor<br>Dr Felicity Bishop – Co-Chief Investigator<br>Professor Hazel Everitt – Co-Chief Investigator
Tenure	Until 30/06/2024.
The Chair, how they are chosen and the Chair's role.	The Chair should have previous experience of serving on trial committees and experience of Chairing meetings, and should be able to facilitate and summarise discussions; knowledge of the disease area would be beneficial.
The responsibilities of the Facilitator	The Facilitator will be a member of staff in Southampton Primary Care Research Centre, University of Southampton. The Facilitator will be responsible for arranging meetings of the TSC, coordinating reports, producing and circulating minutes and action points. The Facilitator will be the central point for all TSC communications between the TSC and other bodies, will be copied into all correspondence between TSC members and will be kept aware of trial issues as they arise.
The responsibilities of the TIP team	The TIP team will produce a short report on the trial before each meeting of the TSC.

[1] Independence is defined in Table 1 of Annexe 1

For peer review only - http://bmjopen.bmj.com/site/about/guidelines.xhtml

CONTENT	DETAILS OF TSC
The responsibilities of the CI and other members of the TMG	The CI (and, if appropriate, other TMG members) is an important member of the TSC and no major decisions should be made without their involvement.
The responsibilities of the observers	Additional observers may be in attendance through (parts of) the TSC meetings in order to provide input on behalf of the TIP team, the trial's Sponsor/Funder or to provide specific relevant expertise.
**5. Relationships**	
Advisory and executive bodies	The TSC is the oversight body and is delegated the roles in Section 2 by the Sponsor. All substantial issues regarding the trial must go to the TSC for consideration.
Payments to TSC members	Members will be reimbursed for reasonable travel costs and other expenses incurred. No other payments or rewards would be given professional members. Honoraria will be paid to lay members according to the INVOLVE guidelines.
The need for TSC members to disclose information about any real or potential competing interests	Any competing interests, both real or potential, should be disclosed. These are not restricted to financial matters – involvement in other trials or intellectual investment could be relevant. Although members may well be able to act objectively despite such connections, complete disclosure enhances credibility. (See Annex 1)<br>TSC members should not use any trial data to inform trading in pharmaceutical shares, and careful consideration should be given to trading in stock of companies with competing products. Changes in declarations of real or potential competing interests should be minuted at the start of each meeting.
**6. Organisation of meetings**	
Expected frequency of TSC meetings	The TSC will meet in person at least yearly if possible. At the request of the TSC, interim meetings, in person or by teleconference, will be organised. Major trial issues may need to be dealt with between meetings, by phone or by email. TSC members should be prepared for such instances.
Attendance of TSC members at meetings	Effort will be made to ensure that all members can attend. The Facilitator will work for a date that enables this. The CI must try to attend all meetings, especially if major actions are expected. Members who cannot attend in person should be encouraged to participate by teleconference. If, at short notice, any TSC members cannot attend then the TSC may still meet if at least two independent members, including the Chair (unless otherwise agreed), will be present, plus also a member of the trial team. If the TSC is considering a major action after such a meeting the TSC Chair should communicate with the absent members, including the CI, as soon after the meeting as possible to check they agree. If they do not, a further teleconference should be arranged with the full TSC.
How TSC meetings will be organised, especially regarding open and closed sessions,	Presence will be usually limited to the TSC members, observers from the Sponsor/Funder, TIP team and the Facilitator. Other attendees may be invited for all or part of the meeting by the TSC including

CONTENT	DETAILS OF TSC
including who will be present in each session	the trial statistician and trial manager. The observers are not members of the TSC but may be invited to provide expert input or to represent the funding bodies involved; other observers will be at the discretion of the TSC and the Facilitator but may include members of the TMG other than the CI.
Can TSC members who cannot attend the meeting input	If the report is circulated before the meeting, TSC members who will not be able to attend the meeting may pass comments to the TSC Chair, Facilitator or TIP team for consideration during the discussions.
What happens to independent members who do not attend meetings	If an independent member does not attend a meeting or provide comments when requested between meetings, it should be ensured that the independent member is available for the next meeting. If an independent member does not attend the next meeting or provide comments when next requested, they should be asked if they wish to remain part of the TSC.
**7. Trial documentation and procedures to ensure confidentiality and proper communication**	
Intended content of material to be considered during meetings	A short report will be prepared by the TIP team. This will report on accrual and any matters affecting the trial. Additionally, the material may include requests *from* the TMG or draft publications. Where relevant, accrual, compliance with follow-up and adherence to treatment may be presented by centre.
Whether reports to the TSC be available before the meeting or only at/during the meeting	It is usually helpful for the TSC to receive the report at least 1 week and preferably at least 2 weeks before any meetings. Different procedures may apply to teleconference meetings.
Responsibility for identifying and circulating external evidence (e.g. from other trials/ systematic reviews)	Identification and circulation of external evidence (e.g. from other trials/ systematic reviews) is not the responsibility of the TSC members; it is a responsibility of the TMG. However, the TSC should continue to be made aware of other data that may impact on a trial.
What will happen to the papers after the meeting	TSC members would be expected to delete, destroy or store securely copies of the reports to and from the TSC, agenda and minutes, as well as copies of communications between meetings. All documentation should be considered confidential. The Facilitator will keep a central record of all minutes, reports and correspondence by the TSC.
**8. Decision making**	
What decisions will be open to the TSC	Possible decisions include:-<br>• No action needed, trial continues as planned<br>• Early stopping due, for example, to clear benefit or harm of a treatment, futility or external evidence.<br>• Stopping recruitment within a subgroup.<br>• Modifying target recruitment, or pre-analysis follow-up, based on any change to the assumptions underlying the original trial sample size calculation (but not on any emerging differences)<br>• Sanctioning and/or proposing protocol changes
How decisions or	Every effort should be made to achieve consensus. The role of the

CONTENT	DETAILS OF TSC
recommendations will be reached within the TSC	Chair is to summarise discussions and encourage consensus; therefore, it is usually best for the Chair to give their own opinion last.<br>It is important that the implications (e.g. ethical, statistical, practical, financial) for the trial be considered before any decision is made.
When the TSC is quorate for decision-making	At least two independent members of the TSC should be present including the Chair, plus the CI if a major action is to be considered.
**9. Reporting**	
To whom will the TSC report their recommendations/decisions, and in what form	The TSC will report their decisions (via the Facilitator) to the TMG who will be responsible for implementing any actions resulting. The TSC may also provide feedback to the Sponsor/Funder. Copies of communications will pass through the Facilitator.
Whether minutes of the meeting be made and, if so, by whom and where they will be kept	Notes of key points and actions will be made by the Facilitator. This will include details of whether potential competing interests have changed for any attendees since the previous meeting. The draft minutes will be initially circulated for comment to those TSC members who were present at the meeting. The TSC Chair will sign off the final version of minutes or notes.
**10. After the trial**	
Publication of results	The TSC will oversee the timely analysis, writing up and publication of the main trial results. The independent members of the TSC will have the opportunity to read and comment on the proposed main publications of trial data prior to submission and abstracts and presentations during the trial.
The information about the TSC that will be included in published trial reports	TSC members will be named and their affiliations listed in the main report, unless they explicitly request otherwise.

For peer review only - http://bmjopen.bmj.com/site/about/guidelines.xhtml

Abbreviations and glossary

AE	Adverse event
CF	Consent form
CI	Chief Investigator
CRF	Case Report Form
CTA	Clinical Trials Authorisation
CTU	Clinical Trials Unit
DMC	Data Monitoring Committee
HE	Health Economics
ISRCTN	International standard randomised controlled trial number
MRC	Medical Research Council
NHS	National Health Service
PI	Principal Investigator
PIS	Patient information Sheet
QL	Quality of life
SAE	Serious adverse event
SOP	Standard operating procedures
SSA	Site specific assessment
TMG	Trial Management Group
TSC	Trial Steering Committee

For peer review only - http://bmjopen.bmj.com/site/about/guidelines.xhtml

Annexe 1: Agreement and competing interests form for independent members

TIP Trial Steering Committee: Agreement to join the Trial Steering Committee as an independent member and disclosure of potential competing interests

Please complete the following document and return to the TSC Facilitator.

(please initial box to agree)

☐ I have read and understood the TSC Charter version 1.0, dated 22 April 2022

☐ I agree to join the Trial Steering Committee for this trial as an independent member

☐ I agree to treat all sensitive trial data and discussions confidentially

The avoidance of any perception that independent members of a TSC may be biased in some fashion is important for the credibility of the decisions made by the TSC and for the integrity of the trial.

Potential competing interests should be disclosed via the via the Primary Care Research Centre. In many cases simple disclosure up front should be sufficient. Otherwise, the (potential) independent TSC member should remove the conflict or stop participating in the TSC. **Table 1** lists potential competing interests.

☐ **No,** I have no potential competing interests to declare
☐ **Yes,** I have potential competing interests to declare (please detail below)

Please provide details of any potential competing interests:

____

____

____

Name: ____________________________

Signed: ___________________________ Date: ______________

Table 1: Potential competing interests for independent members

- Stock ownership in any commercial companies involved
- Stock transaction in any commercial company involved (if previously holding stock)
- Consulting arrangements with the Sponsor/Funder
- Ongoing advisory role to a company providing drugs to the trial
- Frequent speaking engagements on behalf of the intervention
- Career tied up in a product or technique assessed by trial
- Hands-on participation in the trial
- Involvement in the running of the trial
- Emotional involvement in the trial
- Intellectual conflict e.g. strong prior belief in the trial's experimental arm
- Involvement in regulatory issues relevant to the trial procedures
- Investment (financial or intellectual) or career tied up in competing products
- Involvement in the writing up of the main trial results in the form of authorship

Note: This TSC charter was developed using MRC CTU template TSC Charter version 1.02, 13-Mar-2006

For peer review only - http://bmjopen.bmj.com/site/about/guidelines.xhtml

Annexe 2: Agreement and competing interests form for non-independent members

TIP Trial Steering Committee: Agreement to join the Trial Steering Committee as an independent member and disclosure of potential competing interests

Please complete the following document and return to the Facilitator.

(please initial box to agree)

☐	I have read and understood the TSC Charter version 1.0, dated 22 April 2022
☐	I agree to join the Trial Steering Committee for this trial as an non-independent member
☐	I agree to treat all sensitive trial data and discussions confidentially

The avoidance of any perception that members of a TSC may be biased in some undisclosed fashion is important for the credibility of the decisions made by the TSC and for the integrity of the trial.

Possible competing interests should be disclosed via the Primary Care Research Centre. In many cases simple disclosure up front should be sufficient. Otherwise, the (potential) independent TSC member should remove the conflict or stop participating in the TSC. Table 1 lists potential competing interests.

☐ **No,** I have no competing interests to declare other than involvement in the trial
☐ **Yes,** I have competing interests to declare (please detail below)

Please provide details of any competing interests:

____

____

____

Name: ______________________________

Signed: ____________________________ Date: ________________

Table 1: Potential competing interests for non-independent members

- Stock ownership in any commercial companies involved
- Stock transaction in any commercial company involved (if previously holding stock)
- Consulting arrangements with the Sponsor/Funder
- Ongoing advisory role to a company providing drugs to the trial
- Frequent speaking engagements on behalf of the intervention
- Intellectual conflict e.g. strong prior belief in the trial's experimental arm
- Involvement in regulatory issues relevant to the trial procedures
- Investment (financial or intellectual) in competing products

Note: This TSC charter was developed using MRC CTU template TSC Charter version 1.02, 13-Mar-2006

For peer review only - http://bmjopen.bmj.com/site/about/guidelines.xhtml

Annexe 3: Agreement and confidentiality agreement for observers

TIP Trial Steering Committee: Agreement to attend the Trial Steering Committee and treat all information confidentially

Please complete the following document and return to the Facilitator.

(please initial box to agree)

☐	I have received a copy of the TSC Charter version 1.0 22 April 2022
☐	I agree to attend the Trial Steering Committee meeting on ___/____/______
☐	I agree to treat as confidential any sensitive information gained during this meeting unless explicitly permitted

Name: ___________________________

Signed: __________________________ Date: ______________

Note: This TSC charter was developed using MRC CTU template TSC Charter version 1.02, 13-Mar-2006

For peer review only - http://bmjopen.bmj.com/site/about/guidelines.xhtml

Annexe 4: Summarise changes from previous version

Version 1.0

This is version 1.0 of the TSC charter for this trial. There are no changes to be reported.

For peer review only - http://bmjopen.bmj.com/site/about/guidelines.xhtml

Online Supplementary File 5

Stop-Go Progression Criteria

Progression criteria are based on recruitment rates 6 months after commencing patient recruitment:

• GREEN: Recruited 21 practices and 420 patients, with a good pipeline. Continue as planned.

• AMBER: Recruited 15-20 practices and at least 150 patients, with a good pipeline. Discuss with TSC and funder possible mitigating actions, e.g., increase staff time on recruitment activities, expand to other CRNs, shorten patient follow-up period.

• RED: Recruit <15 practices and <150 patients. Discuss with TSC and funder to explore all possible avenues to save the trial. If none deemed feasible, then stop.

For peer review only

Online Supplementary File 6

Plans to give access to the full protocol, participant level-data and statistical code

The protocol will be published in an open access journal. We will seek patient and practitioner consent to deposit data in a data archive e.g., for secondary analysis. For participants who consent for their data to be deposited in a data archive, we will take the necessary steps to pseudonymize the data prior to deposit. Data will be deposited in Pure, the University of Southampton's online data repository, where access will be restricted through gatekeepers (the chief investigators) to suitably qualified individuals with appropriate protocols in place. Statistical code will not be deposited as the pseudonymisation process alters the dataset in a way that impacts the applicability of the statistical code.

For peer review only
